# Dioscin Inhibits HSC-T6 Cell Migration via Adjusting SDC-4 Expression: Insights from iTRAQ-Based Quantitative Proteomics

**DOI:** 10.3389/fphar.2017.00665

**Published:** 2017-09-20

**Authors:** Lianhong Yin, Yan Qi, Youwei Xu, Lina Xu, Xu Han, Xufeng Tao, Shasha Song, Jinyong Peng

**Affiliations:** College of Pharmacy, Dalian Medical University Dalian, China

**Keywords:** cell migration, dioscin, hepatic stellate cells, iTRAQ, liver fibrosis, syndecan-4

## Abstract

Hepatic stellate cells (HSCs) migration, an important bioprocess, contributes to the development of liver fibrosis. Our previous studies have found the potent activity of dioscin against liver fibrosis by inhibiting HSCs proliferation, triggering the senescence and inducing apoptosis of activated HSCs, but the molecular mechanisms associated with cell migration were not clarified. In this work, iTRAQ (isobaric tags for relative and absolution quantitation)-based quantitative proteomics study was carried out, and a total of 1566 differentially expressed proteins with fold change ≥2.0 and *p* < 0.05 were identified in HSC-T6 cells treated by dioscin (5.0 μg/mL). Based on Gene Ontology classification, String and KEGG pathway assays, the effects of dioscin to inhibit cell migration via regulating SDC-4 were carried out. The results of wound-healing, cell migration and western blotting assays indicated that dioscin significantly inhibit HSC-T6 cell migration through SDC-4-dependent signal pathway by affecting the expression levels of Fn, PKCα, Src, FAK, and ERK1/2. Specific SDC-4 knockdown by shRNA also blocked HSC-T6 cell migration, and dioscin slightly enhanced the inhibiting effect. Taken together, the present work showed that SDC-4 played a crucial role on HSC-T6 cell adhesion and migration of dioscin against liver fibrosis, which may be one potent therapeutic target for fibrotic diseases.

## Introduction

Hepatic stellate cells (HSCs) are a major cell type involved in liver tissue, which make up approximately 5–15% of the total liver cell population. In healthy liver, HSCs with a quiescent exhibit lipid droplets storing large amounts of retinoids (Bansal, 2016). When liver is damaged, HSCs can transfer to an activated, myofibroblast-like cells, lose their retinol stores, become proliferative and contractile, and migrate to the site of injury (Eng et al., 2000). The activated HSCs can produce extracellular matrix, cytokines and growth factors to protect further damage and produce the regeneration of damaged hepatocytes (Rockey, 2001). However, chronic activation of HSCs following persistent liver damage can result in promotion of fibrosis and alter the structure and functionality of the liver (Trautwein et al., 2015; Guo et al., 2017). Liver fibrosis is a reversible process, and senescence of HSCs is a potential mechanism of fibrosis reversal (Tsukada et al., 2006) for the anti-fibrotic therapies. The experimental and pharmacological approaches on inhibiting cell proliferation and migration of HSCs against liver fibrosis have been tested (Yang et al., 2017). HSCs migration is an important bioprocess for the development liver fibrosis (Zhao X. et al., 2017). Thus, looking for potent drug to inhibit HSCs migration against liver fibrosis is important.

Some natural products including berberine, silymarin, and artemisinin extracted from medicinal plants have protective effects against liver fibrosis (Wang et al., 2016; Sokar et al., 2017; Zhao X.K. et al., 2017). Dioscin, a steroid saponin, is widely prevalent in some herbs (Xu et al., 2015). Our previous works revealed that dioscin has potent effects against liver fibrosis by attenuating HSCs activation, triggering the senescence of activated HSCs, and adjusting various signals (Liu et al., 2015; Zhang et al., 2015). However, the effect and mechanisms of dioscin on HSCs cell migration were not clarified in our best knowledge.

The methods of quantitative proteomics have been widely used in the mechanistic study of traditional Chinese medicines to find disease-specific targets and biomarkers (Li et al., 2014). Among them, isobaric tags for relative and absolution quantitation (iTRAQ) combining with multidimensional liquid chromatography and tandem MS analysis is one of the most powerful methodologies for investigating drug targets and molecular mechanisms (Wang T. et al., 2017). Thus, iTRAQ may be a plausible way for the discovery of drug-target of dioscin on HSCs cell migration.

Therefore, the aim of the study was to investigate the effect and potent drug targets of dioscin on cell migration of HSCs against liver fibrosis by using iTRAQ-based quantitative proteomics study.

## Materials and Methods

### Chemicals

Dioscin was isolated from *Dioscorea nipponica* Makino in our laboratory (Yin et al., 2010). Bicinchoninic acid (BCA) protein assay kit, 4′,6-diamidino-2-phenylindole (DAPI), tris(hydroxymethyl)aminomethane (Tris), and sodium dodecyl sulfate (SDS) were purchased from Sigma Chemical Co. (St. Louis, MO, United States). shRNA plasmid vector was provided by Shanghai GenePharma Co., Ltd., (Shanghai, China).

### Cell Culture and Treatment

HSC-T6 cells with the characteristics of activated HSCs phenotype were purchased from the Shanghai Institutes for Biological Sciences (Shanghai, China). It was cultured in Dulbecco’s modified Eagle’s medium (DMEM) supplemented with 10% FBS at 37°C in a humidified atmosphere of 5% CO_2_ and 95% air. The HSC-T6 cells were plated into 96-well plates at the density of 5 × 10^4^ cell/mL and treated with dioscin at the concentrations of 1.25, 2.5, and 5.0 μg/mL for 24 h. Cell viability was measured using MTT assay.

### Protein Preparation and iTRAQ Labeling

The iTRAQ reagent multiplex kit was bought commercially (Applied Biosystems, Foster City, CA, United States). The cells of control and dioscin (5.0 μg/mL) groups with two cell culture replicates were harvested and washed with PBS. Then, the cells were treated with radioimmunoprecipitation assay lysis buffer (50 mM Tris–HCl, 1% SDC, 150 mM NaCl, 0.1% Triton X-100, pH 8.0) supplemented with 1 mM PMSF (phenylmethane sulfonyl fluoride). Next, protein solubilization was achieved by ultrasonic, and cellular debris was removed by centrifugation (12,000 × *g*, 20 min, 4°C). Then, protein concentration of the middle layer was quantified by BCA Protein Assay Kit. Further, two independent biological replicates (200 μg) of each group were prepared by FASP (filter aided sample preparation), and iTRAQ labeling was performed according to the manufacturer’s introduction (Applied Biosystems, Foster City, CA, United States). The protein samples of dioscin-treated group (5.0 μg/mL) were labeled with iTRAQ 117 and iTRAQ 118, and the protein samples of control group were labeled with iTRAQ 119 and iTRAQ 121. After that, the labeled digests were pooled and dried by vacuum centrifuge.

### High-pH Reversed-Phase Chromatography

These peptide samples labeled by iTRAQ were re-dissolved in 200 μL of buffer A solution (20 mM HCOONH_4_, pH 10). Then, 100 μL of dissolved sample was injected to HPLC and through a Phenomenex column (Gemini-NX 3u C18 110 A; 150 × 2.00 mm). The ultraviolet detector was set at 214 nm/280 nm, and a linear gradient elution model from buffer A and buffer B [20 mM HCOONH_4_, 80% acrylonitrile (ACN), pH 10] was applied at a flow rate of 0.2 mL/min. From 5 to 50% of buffer B, a total of 24 fractions were collected. Then, the fractions with 50% TFA acidification were dried by vacuum centrifuge for RPLC-MS analysis.

### RPLC-MS Analysis

Peptide fractions were dissolved in sample dissolution solution composed of 0.1% FA (formic acid), 2% ACN. After vortex, the mixture solution was centrifuged at 13,200 × *g* for 10 min at 4°C. The upper layer of the liquid was analyzed by HPLC. The peptides were separated by a ZORBAX 300SB-C18 column (75 μm × 150 mm, 3 μm, 100 Å, Microm, Auburn, CA, United States), and a linear gradient elution model was carried out of mobile phase A (0.1% FA, 5% ACN) and mobile phase B (0.1% FA, 95% ACN), from 5 to 40% of mobile phase B for 70 min, at a flow rate of 300 nL/min.

In MS analysis, a Triple TOF 5600 system (AB SCIEX, Foster City, CA, United States) set at Information Dependent Mode was applied. The MS spectra were acquired across the range of 400–1250 *m/z* in high resolution mode (>30,000), using 250 ms accumulation time per spectrum. A maximum of 20 precursors per cycle were chosen for fragmentation from each MS spectrum with 100 ms minimum accumulation time for each precursor and dynamic exclusion for 20 s. Tandem mass spectra were recorded in high sensitivity mode (resolution >15,000), with turned on rolling collision and iTRAQ reagent collision energy adjustment.

### Data Analysis

The peptide data were analyzed by Protein Pilot Software 4.5, and the Paragon protein database search algorithm (Applied Biosystems Sciex) was used. Based on this software, the peptide data from iTRAQ were converted into the differential analysis data on protein level. The parameters for the analysis were set as follows: Database: UniProtKB/Swiss-Prot FASTA (it was released November 15, 2013 and consists of 28,854 rat sequences), Cys alkylation: MMTC, ID focus: biological modifications, Digestion: trypsin, Search effort: thorough ID. The protein data were used for quantification from the four groups including 119/117, 121/117, 119/118, and 121/118. The data between dioscin group/control group with the relative expression of >2.0 or <0.5 and *p*-value <0.05 was chosen to ensure up- and down-regulation authenticity. For biological pathway analysis, the GenInfo numbers of these differently expressed proteins were imported into KEGG database^1^ and each protein sequence and functional information was obtained from UniProt databases^2^. Protein network analysis of the differentially expressed proteins was carried out using the online tool String 9.1.

### Immunofluorescence Assay of SDC-4

The HSC-T6 cells were fixed with 2% paraformaldehyde for 15 min and incubated with 0.5% Triton X-100 for 15 min. Next, the cells were blocked with 4% BSA at room temperature for 2 h and incubated with rabbit anti-SDC-4 antibody in a humidified chamber overnight at 4°C. After washing with PBS for three times, the cells were incubated with a fluorescein-labeled secondary antibody for 1.5 h. The cell nuclei were then stained with DAPI (5.0 μg/mL) for 10 min. Finally, all samples were examined by fluorescence microscopy (OLYMPUS, Japan).

### Wound-Healing Assay

The HSC-T6 cells were cultured in six-well plates for 24 h at 37°C. Wounds were created in the cell monolayer, and the cell debris were removed with PBS. Next, the cells were treated with different concentrations of dioscin (0.375, 0.625, and 1.25 μg/mL) for 24 h. Then, the plates were washed with PBS to remove the dead cells. After that, the images were taken by the fluorescence microscope (OLYMPUS, Japan).

### Cell Migration Assay

Boyden chambers containing a transwell membrane filter with an 8 μm size pore (Corning Costa Corp., Cambridge, MA, United States) was used in cell migration assay. The HSC-T6 cells were seeded to the apical side of the chamber with 200 μL of DMEM with different concentrations of dioscin (0.375, 0.625, and 1.25 μg/mL), and the basolateral side of the chamber was filled with 500 μL of medium containing 10% FBS. After 24 h culture at 37°C, the cells adherent to the upper surface of the filter were swept by cotton swabs, then the cells passed through to the lower surface of the filter were fixed with methanol for 10 min. After that, the cells were stained with crystal violet and counted under a microscope in five random fields, irrespective of staining intensity. The images were taken by the fluorescence microscope (OLYMPUS, Japan).

### Real-Time PCR Assay

Total RNA from HSC-T6 cells was extracted using RNAiso Plus (Transgen Biotech, Beijing, China) following the manufacturer’s protocol, and the purity of the extracted RNA was determined. The primers of COL3A1 [collagen type III alpha 1 chain], COL1A1 (collagen type I alpha 1 chain), α-SMA (α-smooth muscle actin), SDC-4 (syndecan-4), and GAPDH are listed in **Table 1**. cDNA was synthesized using a PrimeScript^®^ RT reagent kit according to the manufacturer’s instructions (Transgen Biotech, Beijing, China). For real-time PCR assay, SYBR^®^ Premix Ex Taq^TM^ II (Transgen Biotech, Beijing, China) was used and subjected to quantitative PCR in an ABI 7500 Real Time PCR System, and the data was analyzed using System SDS software (Applied Biosystems, United States).

**Table 1:** Primers information used for real-time PCR assay.

Primers	Forward primer (5′–3′)	Reverse primer (5′–3′)
COL3A1	TTTGGCACAGCAGTCCAATGTA	GACAGATCCCGAGTCGCAGA
COL1A1	GACATGTTCAGCTTTGTGGACCC	AGGGACCCTTAGGCCATTGTGTA
α-SMA	AGCCAGTCGCCATCAGGAAC	GGGAGCATCATCACCAGCAA
SDC-4	GGGCAAGAAACCCATCTACA	TGAAGTCCAAGCAGCACTCA
GAPDH	GGCACAGTCAAGGCTGAGAATG	ATGGTGGTGAAGACGCCAGTA

### Western Blotting Assay

The HSC-T6 cells (2 × 10^5^ cells/mL) were plated in six-well plates and treated with dioscin (1.25, 2.5, and 5.0 μg/mL). Total proteins from different groups were extracted by cell lysis buffer containing PMSF. Next, the lysates were centrifuged at 12,000 × *g* for 10 min at 4°C. Then, the total proteins were loaded onto the SDS-PAGE gel (10–15%), separated electrophoretically and transferred to the PVDF membrane (Millipore, United States). The PVDF membranes were put into 5% dried skim milk for 3 h at room temperature, incubated overnight at 4°C with the primary antibodies (**Table 2**), then incubated with horseradish peroxidase-conjugated antibodies for 2 h at room temperature. Proteins were detected by enhanced chemiluminescence method and photographed using the Bio-Spectrum Gel Imaging System (UVP, United States), which were normalized with GAPDH as an international control. Three blots of each protein were performed, and five lanes were quantified. Where applicable, the image intensities of specific bands were quantified by Image-Pro Plus software (Media Cybernetics, United States). IOD value of target protein versus IOD value of GAPDH was used to eliminate the variation of protein expression.

**Table 2:** Antibody information used in the present study.

Antibody	Source	Dilutions	Company	Reference
SDC-4	Rabbit	1:100	Proteintech Group, Chicago, IL, United States	Song et al., 2012
Fibronectin	Rabbit	1:1000	Abcam, United States	Na et al., 2012
FAK	Rabbit	1:500	Proteintech Group, Chicago, IL, United States	Sun et al., 2017
PY387-FAK	Rabbit	1:500	Proteintech Group, Chicago, IL, United States	Sun et al., 2017
Src	Rabbit	1:500	Proteintech Group, Chicago, IL, United States	Wang Y. et al., 2017
PKCα	Rabbit	1:500	Proteintech Group, Chicago, IL, United States	Lim et al., 2003
p-PKCα	Rabbit	1:5000	Abcam, United States	Lim et al., 2003
ERK1/2	Rabbit	1:500	Proteintech Group, Chicago, IL, United States	Sun et al., 2017
p-ERK1/2	Rabbit	1:500	Proteintech Group, Chicago, IL, United States	Sun et al., 2017

### SDC-4 shRNA Transfection Experiment

The method of shRNA transfection was used to down-regulate SDC-4 expression level. shRNA [pGPU6/GFP/Neo, SDC4-Rat-462 (5′–3′): GGTCTTGGCAGCTCTG ATTGT] was transfected into HSC-T6 cells using Lipofectamine 2000 reagent (DNA: Lipofectamine^TM^ 2000, 1 μg: 2.5 μL). After 24 h of transfection, the expression level of SDC-4 was detected. Wound-healing and cell migration assays were also carried out.

### Statistical Analysis

Statistical analysis data were presented as mean ± standard deviation (SD). Student’s *t*-test was used for statistical analysis to evaluate the significant difference between different groups. Statistical software SPSS 18.0 was applied. The significance change was evaluated and considered at *p* < 0.05 using ANOVA and Tukey’s *post hoc* test.

## Results

### Dioscin Inhibits HSC-T6 Cell Viability

The effect of dioscin on inhibiting the viability of HSC-T6 cells was investigated, and the results showed that the cell viability was significantly inhibited by dioscin with a dose-dependent manner (Supplementary Figure 1A). In addition, the inhibitory effect of dioscin on fibrogenesis was evaluated, and the results showed that the mRNA levels COL3A1, COL1A1, and α-SMA were greatly reduced by the compound (Supplementary Figures 1B–D). Dioscin at the concentration of 5.0 μg/mL significantly inhibited HSCs cell proliferation and activation.

### iTRAQ Quantification

After iTRAQ analysis, a total of 252,030 spectra were identified in HSC-T6 cells, including 99,649 identified peptides, 9255 proteins before grouping (total prot score > unused cutoff prot score) and 5240 protein detected (unused prot score < cutoff prot score) (Supplementary Figure 2A). The protein mass distribution, isoelectric point distribution, peptide length distribution, peptide number distribution, and sequences coverage of detected protein were assayed, and more than 2000 proteins have the MW around 20–60 kDa, 1415 proteins have the pI between 5 and 6, length of peptides were 6∼16, 709 proteins only have one peptide, and 493 proteins have the sequences coverage 40∼100% (Supplementary Figures 2B–F). Then, the protein abundance distribution showed the distribution of the differentially expressed protein (Supplementary Figure 2G). Red for the up-regulated proteins and green for the down-regulated proteins). Furthermore, the most commonly used statistical model for iTRAQ labeling reproducibility assay is the coefficient of variation (CV), and the data with CV in the range of ±50% can be considered within the acceptable range. In the study, the CV assay of the identified proteins indicated that the cumulative percentage was up to 96% (Supplementary Figure 2H), and the data out of the bounds were deleted from further assay. Then, a fold change cutoff at 2.0 was set to identify molecules whose expression was significantly differently regulated. The fold change was the ratio of protein differently produced in dioscin-induced cells relative to those of in control group. The values of Unused (ProtScore) > 1.3 and *p*-value < 0.05 were taken as significant screening. Unused > 1.3 means protein confidence > 95%. Total of 1566 differentially expressed proteins including 702 up-regulated and 864 down-regulated proteins with the fold change ≥ 2.0 were identified, which are listed in Supplementary Table 1. When the fold change was more than 4.0, total of 132 up-regulated and 262 down-regulated differentially expressed proteins were identified.

### Gene Ontology Classification

**FIGURE 1:**
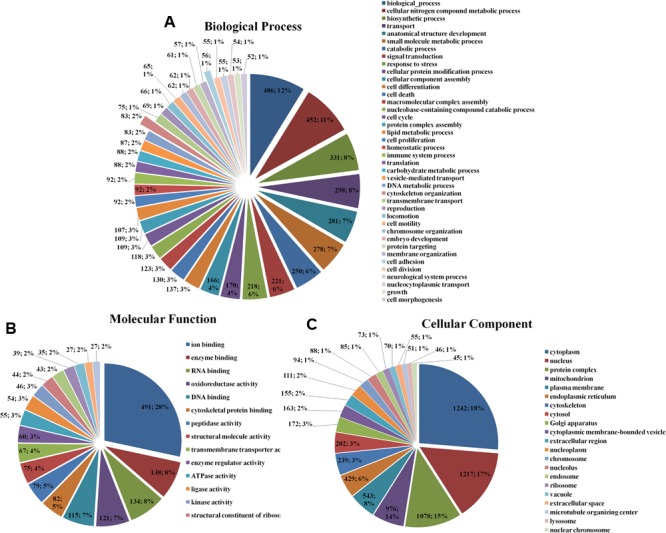
Gene Ontology classification of the differentially expressed proteins in HSC-T6 cells treated by dioscin. **(A)** Biological process; **(B)** molecular function; **(C)** cellular component. The data are expressed as the numbers of proteins and the corresponding percentages.

The enrichment assay of the differently expressed proteins with the fold changes ≥ 2.0 was carried out using Gene Ontology (GO) method. The biological processes of cellular nitrogen compound metabolic process (486; 12%), biosynthetic process (452; 11%), cytoskeleton organization (83; 12%), cell adhesion (56; 1%) (**Figure 1A**), the molecular functions of ion binding (491; 28%), enzyme binding (138; 8%), RNA binding (134; 8%) (**Figure 1B**), and the cellular components of cell (1242; 18%), intracellular (1217; 17%), and organelle (1078; 15%) (**Figure 1C**) of the differently expressed proteins were found.

**FIGURE 2:**
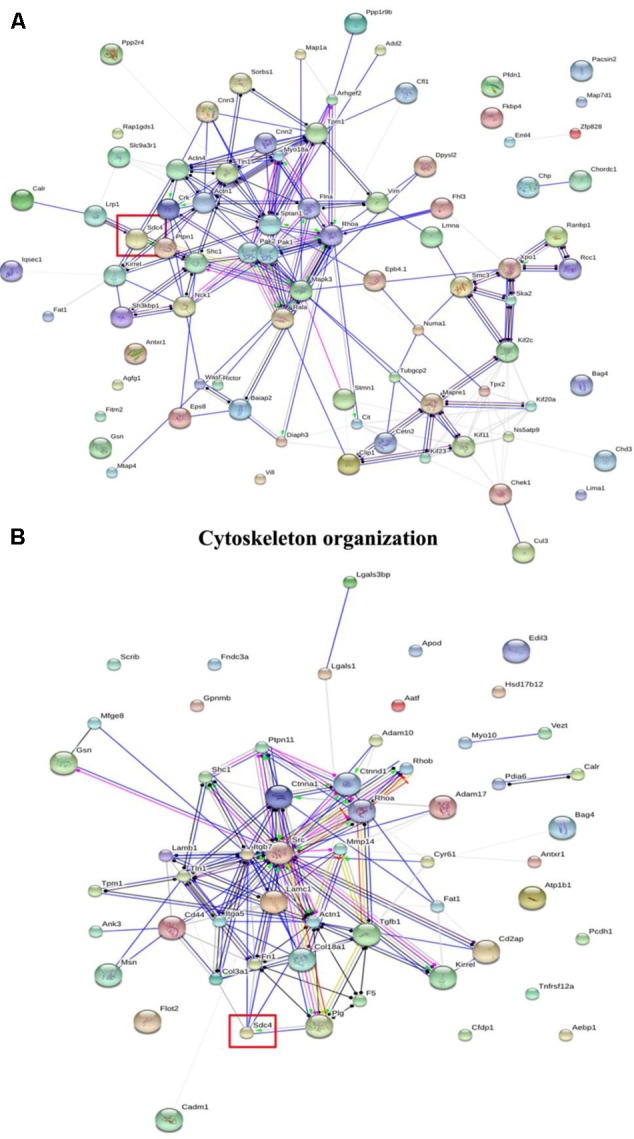
The interaction of the differentially expressed proteins in cytoskeleton organization and cell adhesion of GO classification analyzed by String analysis. **(A)** Cytoskeleton organization. **(B)** Cell adhesion. The color of the connective lines indicates the type of evidence for the connection: A red line indicates the presence of fusion evidence; a green line, neighborhood evidence; a blue line, co-occurrence evidence; a purple line, experimental evidence; a yellow line, text mining evidence; a light blue line, database evidence; a black line, co-expression evidence.

In GO classifications, two biological processes including cytoskeleton organization and cell adhesion associated with cell migration were screened (Sun et al., 2017). Furthermore, the networks of the differentially expressed proteins were further analyzed using String 9.1, and the results of cytoskeleton organization and cell adhesion are shown in **Figure 2**. Among the differentially expressed proteins, SDC-4 showed the highest fold change (dioscin group/control group = 0.0348). In cytoskeleton organization (**Figure 2A**), SDC-4 was closely related to Shc1 (sodium/potassium-transporting ATPase subunit beta-1), Actn4 (alpha-actinin-4), Actn1 (alpha-actinin-1), and Lrp1 (low density lipoprotein receptor-related protein 1). In cell adhesion (**Figure 2B**), SDC-4 was closely related to COL3A1, Fn1 (fibronectin 1), actn1, plg (plasminogen), Lamc1 (laminin, gamma 1 precursor), and Itgb7 (integrin, beta 7 precursor).

### KEGG Ontology Assignments

KEGG ontology assignments were used to classify functional annotations of the identified proteins to further understand their biological functions, which were mapped to 230 pathways in the KEGG database (Supplementary Table 2).

**FIGURE 3:**
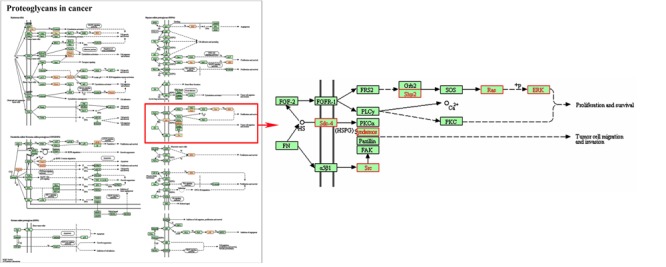
Pathway analysis using the KEGG database. KEGG ID: proteoglycans in cancer (rno05205). Proteins with red shading were the differentially expressed genes.

In addition, three signal pathways associated with SDC-4 including proteoglycans in cancer (rno05205), cell adhesion molecules (rno04514, Supplementary Figure 3A), ECM-receptor interaction (rno04512, Supplementary Figure 3B) were found. In the map of proteoglycans in cancer (**Figure 3**), the proteins of Fn, SDC-4, PKCα, Src, FAK, and ERK were connected with cell migration and invasion. All the results indicated that SDC-4 plays important role in dioscin against liver fibrosis through regulating cell adhesion and cell migration.

### Dioscin Reduces SDC-4 Level in HSCs

**FIGURE 4:**
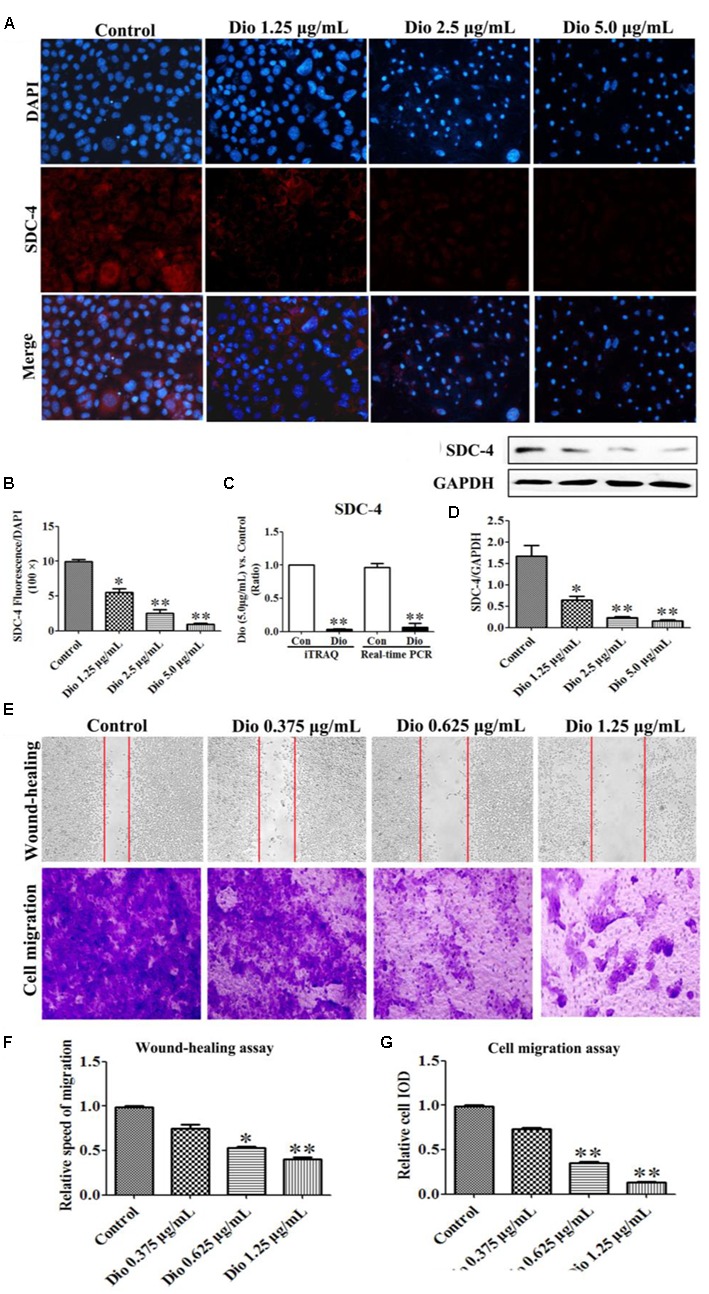
Effects of dioscin on the expression levels of SDC-4 in HSC-T6 cells based on immunofluorescence (×400 original magnification) assay **(A,B)**, iTRAQ and real-time PCR assays **(C)**, and western blotting assay **(D)**. Effect of dioscin on the migration of HSC-T6 cells **(E–G)**. Data are presented as mean ± SD (*n* = 3). ^∗^*p* < 0.05, ^∗∗^*p* < 0.01 compared with control group.

As expected, dioscin significantly suppressed the expression level of SDC-4 based on an immunofluorescence assay in HSC-T6 cells (**Figures 4A,B**). In addition, the expression level of SDC-4 was also down-regulated by dioscin based on western blotting, iTRAQ assay and real-time PCR assays (**Figures 4C,D**). Therefore, we speculated that dioscin can effectively inhibit HSC-T6 cell adhesion and migration via SDC-4 signal against liver fibrosis.

### Dioscin Inhibits HSC-T6 Migration

The wound healing and cell migration assays were carried out to evaluate the effect of dioscin on inhibiting HSC-T6 cells migration. The results shown in **Figures 4E–G** indicated that the migration distance and relative speed of HSC-T6 cells treated by dioscin (0.625 and 1.25 μg/mL) were significantly lower than control group. In addition, the cell migration with crystal violet staining and relative speed IOD showed that dioscin significantly suppressed the migratory capability of HSC-T6 cells.

### Dioscin Inhibits SDC-4 Signaling

**FIGURE 5:**
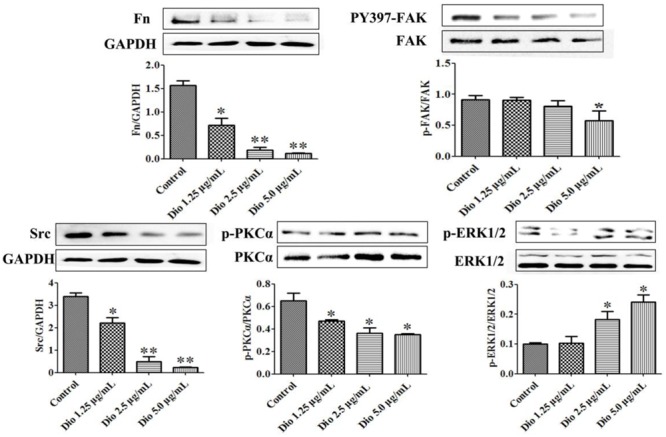
Dioscin inhibited HSC-T6 adhesion and migration via regulating SDC-4 signal pathway. Data are presented as mean ± SD (*n* = 3).^∗^*p* < 0.05 and ^∗∗^*p* < 0.01 compared with control group. The cropped gels are used and the original blots were provided in the Supplementary Figure 4.

After 24 h treatment, the expression levels of the proteins in SDC-4 signaling were detected by western blotting assay. Further, statistical software SPSS 18.0 was applied in the study, three blots of each protein were performed, and five lanes were quantified. The results showed that SDC-4-mediated activation of PKCα (p-PKCα) was increased by dioscin, and the expression levels of Fn, Src (proto-oncogene tyrosine-protein kinase) were down-regulated by dioscin. In addition, the expression level of PY397-FAK (FAK activation) was reduced by dioscin, and inhibition of FAK activation increased the expression levels of p-ERK1/2 in HSC-T6 cells treated by dioscin (**Figure 5**). Thus, dioscin significantly inhibit HSC-T6 cell migration through a SDC-4-dependent signal pathway via affecting the expression levels of Fn, PKCα, Src, PY397-FAK, and p-ERK1/2.

### Dioscin Slightly Enhances the Inhibiting Effect of SDC-4 shRNA on HSC-T6 Migration

**FIGURE 6:**
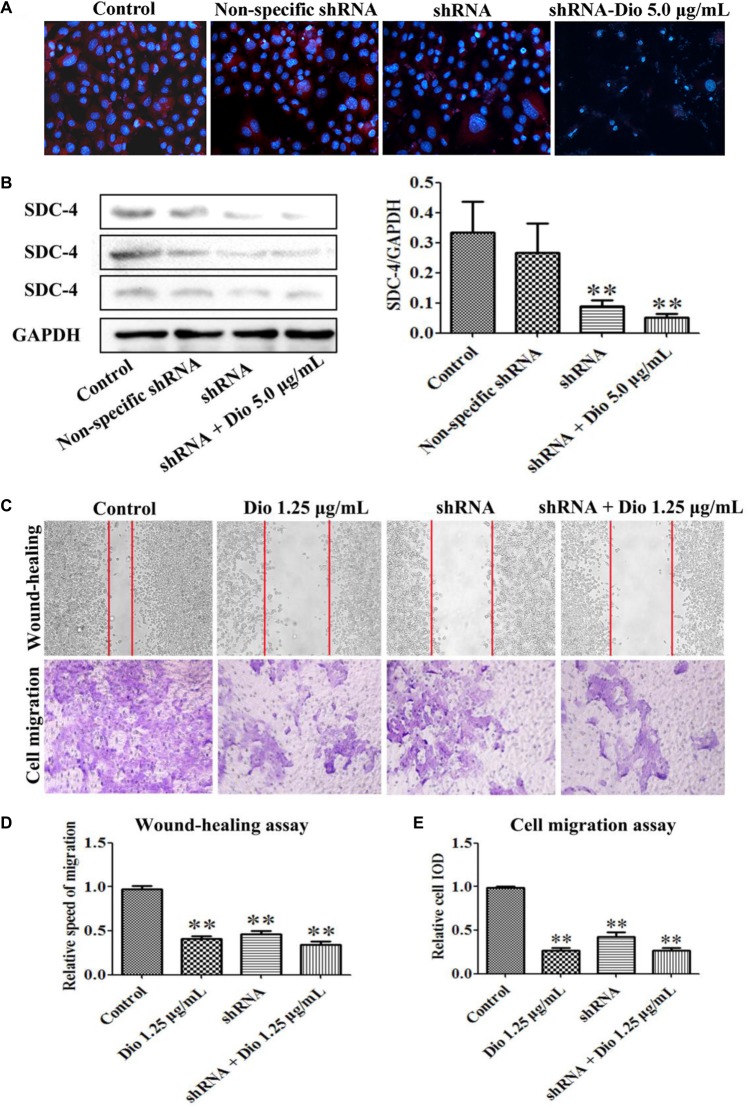
Effects of SDC-4 shRNA and dioscin on SDC-4 expression. Effects of dioscin on SDC-4 level based on immunofluorescence assay (×400 original magnification) **(A)** and western blotting assay **(B)** in HSC-T6 cells. **(C–E)** Wound-healing and cell migration assays of HSC-T6 cells treated by dioscin or SDC-4 shRNA. Data are presented as mean ± SD (*n* = 3). ^∗^*p* < 0.05 and ^∗∗^*p* < 0.01 compared with control group.

We found that SDC-4 shRNA-462 down-regulated SDC-4 expression in HSC-T6 cells, and the inhibition effect of shRNA + dioscin (5.0 μg/mL) was better than shRNA (**Figures 6A,B**). The results of wound-healing and cell migration assays indicated that blockade of SDC-4 expression significantly inhibited HSC-T6 cells migration compared with control group (**Figures 6C,D**). However, the migration inhibition effect of SDC-4 shRNA-462 transfection was altered compared with dioscin (1.25 μg/mL) group, but statistics showed that they did not reach significant levels. Furthermore, the relative speed and relative cell IOD of migration assays indicated that dioscin at the dose of 1.25 μg/mL slightly enhanced the inhibition effect of SDC-4 shRNA-462 on HSC-T6 cell migration (**Figure 6E**). All the results indicated that dioscin inhibited HSC-T6 cell migration against liver fibrosis via adjusting SDC-4.

## Discussion

HSCs play an important role in liver fibrosis (Gressner, 1996; Huang et al., 2017), and activated HSCs are considered as a major target for drug therapy of liver fibrosis. In the early stage of liver injury, lipid droplets of HSCs decrease in size and number, while cell proliferation, migration rate, and extracellular matrix production are increased (Gu et al., 2016; Kim and Jung, 2016). Our previous studies showed that dioscin can alleviate liver fibrosis by attenuating HSCs activation, inhibiting proliferation and reducing extracellular matrix production (Liu et al., 2015; Zhang et al., 2015). Importantly, inhibition of HSC migration can inhibit the development of liver fibrosis and ameliorate liver fibrosis. Therefore, finding new molecules involved in HSC migration can further clarify this process and point new therapeutic targets for the treatment of liver fibrosis. On this account, the mechanisms associated with migration of dioscin on HSC-T6 cells were tested. In this study, we used iTRAQ to obtain valuable evidence on proteomic changes to understand the mechanisms of dioscin against liver fibrosis. A total of 702 up-regulated and 864 down-regulated differentially expressed proteins were founded in HSC-T6 treated by dioscin, which should be useful for us to understand the effect of dioscin on HSCs migration against liver fibrosis.

Cell migration requires integrins to link cytoskeletal actin at focal adhesion of the migrating cells to the substratum (Sun et al., 2017), and the focal adhesion is dynamic during wound healing and cell migration (Wu et al., 2017), which is the most important one. In the present paper, the protein SDC-4 with the highest fold change was selected as the possible target of dioscin against liver fibrosis through inhibiting HSCs migration based on GO classifications. SDC-4 has been described as a component of focal adhesion because of its ability to bind several different matrix molecular including Fn and affect cell migration (Wilcox-Adelman et al., 2002; Chalkiadaki et al., 2009; Na et al., 2012; Lin et al., 2015; Li et al., 2016). Furthermore, the central region of SDC-4 cytoplasmic domain (4V; LGKKPIYKK) binds with phosphatidylinositol 4,5-bisphosphate, and then to regulate PKCα activity (Lim et al., 2003). FAK is activated during integrin activation, and SDC-4 appears to play a vital role in regulating FAK phosphorylation (Song et al., 2012). ERK represents a mitogen-activated protein kinase involved in the activation of cell adhesion and migration, and ERK phosphorylation can be regulated by FAK (Zou et al., 2013). It has been demonstrated that SDC-4/PKCα/FAK/ERK1/2 pathway is crucial in cell adhesion (Lin et al., 2015). In this work, our data indicated that dioscin inhibited the expression level of SDC-4 to regulate the downstream proteins including Fn, FAK, p-PKCα, Src, and p-ERK1/2. To further investigate the role of SDC-4 on HSC-T6 cells migration by dioscin, SDC-4 knockdown using shRNA transfection experiment in HSC-T6 cells was performed. The results demonstrated that SDC-4 was an important regulator in controlling HSC-T6 cells migration, and dioscin inhibited the migration via adjusting SDC-4. These results provided a novel insight of dioscin against liver fibrosis and indicated that SDC-4 mediating focal adhesions was the mechanism of dioscin to inhibit HSC-T6 cells migration.

## Conclusion

iTRAQ-based quantitative proteomics study was used to study the mechanisms of dioscin against liver fibrosis on HSC-T6 cells. The data provided new insights and some candidate biomarkers associated with liver fibrosis. In addition, this study clearly indicated that dioscin inhibited the migration of HSC-T6 cells against liver fibrosis via regulating SDC-4 signal pathway.

## Author Contributions

LY was responsible for the planning, execution of all experiments, and preparation of the manuscript. YQ, YX, and LX were responsible for the preparation, isolation, and bioavailability study of dioscin. XH, XT, and SS were responsible for the western blotting and real-time PCR assays. JP was responsible for the conceptualization, planning, execution, and troubleshooting of the experiments, preparation of the manuscript, and the financial support.

## Conflict of Interest Statement

The authors declare that the research was conducted in the absence of any commercial or financial relationships that could be construed as a potential conflict of interest.

## Footnotes

1 http://www.genome.jp/kegg/

2 http://www.uniprot.org/

## Abbreviations

ERK1/2: extracellular regulated protein kinases
FAK: focal adhesion kinase
Fn: fibronectin
HSCs: hepatic stellate cells
iTRAQ: isobaric tags for relative and absolution quantitation
PKCα: protein kinase Cα
SDC-4: syndecan-4

## References

[B1] Bansal M. B. (2016). Hepatic stellate cells: fibrogenic, regenerative or both? Heterogeneity and context are keys. *Hepatol. Int.* 10 902–908. 10.1007/s12072-016-9758-x 27578210

[B2] Chalkiadaki G. Nikitovic D. Berdiaki A. Sifaki M. Krasagakis K. Pavlos K. (2009). Fibroblast growth factor-2 modulates melanoma adhesion and migration through a syndecan-4-dependent mechanism. *Int. J. Biochem. Cell Biol.* 41 1323–1331. 10.1016/j.biocel.2008.11.008 19110070

[B3] Eng F. J. Friedman S. L. Fibrogenesis I. (2000). New insights into hepatic stellate cell activation: the simple becomes complex. *Am. J. Physiol. Gastrointest. Liver Physiol.* 279 G7–G11. 10898741 10.1152/ajpgi.2000.279.1.G7

[B4] Gressner A. M. (1996). Transdifferentiation of hepatic stellate cells (Ito cells) to myofibroblasts: a key event in hepatic fibrogenesis. *Kidney Int. Suppl.* 54 S39–S45. 8731193

[B5] Gu Y. J. Sun W. Y. Zhang S. Li X. R. Wei W. (2016). Targeted blockade of JAK/STAT3 signaling inhibits proliferation, migration and collagen production as well as inducing the apoptosis of hepatic stellate cells. *Int. J. Mol. Med.* 38 903–911. 10.3892/ijmm.2016.2692 27460897

[B6] Guo Z. R. Li D. Peng H. Y. Kang J. W. Jiang X. Y. Xie X. Y. (2017). Specific hepatic stellate cell-penetrating peptide targeted delivery of a KLA peptide reduces collagen accumulation by inducing apoptosis. *J. Drug Target.* 25 715–723. 10.1080/1061186X.2017.1322598 28447897

[B7] Huang Y. Deng X. Liang J. (2017). Modulation of hepatic stellate cells and reversibility of hepatic fibrosis. *Exp. Cell Res.* 352 420–426. 10.1016/j.yexcr.2017.02.038 28238836

[B8] Kim J. Jung Y. (2016). Thymosin beta 4 is a potential regulator of hepatic stellate cells. *Vitam. Horm.* 102 121–149. 10.1016/bs.vh.2016.04.011 27450733

[B9] Li R. Wu H. Xie J. Li G. Gu R. Kang L. (2016). Syndecan-4 regulates the bFGF-induced chemotactic migration of endothelial cells. *J. Mol. Histol.* 47 503–509. 10.1007/s10735-016-9693-0 27541034

[B10] Li X. Z. Zhang S. N. Wang K. X. Liu S. M. Lu F. (2014). iTRAQ-based quantitative proteomics study on the neuroprotective effects of extract of *Acanthopanax senticosus* harm on SH-SY5Y cells overexpressing A53T mutant α-synuclein. *Neurochem. Int.* 72 37–47. 10.1016/j.neuint.2014.04.012 24795107

[B11] Lim S. T. Longley R. L. Couchman J. R. Woods A. (2003). Direct binding of syndecan-4 cytoplasmic domain to the catalytic domain of protein kinase C alpha (PKC alpha) increases focal adhesion localization of PKC alpha. *J. Biol. Chem.* 278 13795–13802. 10.1074/jbc.M208300200 12571249

[B12] Lin T. J. Lu K. W. Chen W. H. Cheng C. M. Lin Y. W. (2015). Roles of syndecan-4 and relative kinases in dorsal root ganglion neuron adhesion and mechanotrans- duction. *Neurosci. Lett.* 592 88–93. 10.1016/j.neulet.2015.02.058 25757361

[B13] Liu M. Xu Y. W. Han X. Yin L. H. Xu L. N. Qi Y. (2015). Dioscin alleviates alcoholic liver fibrosis by attenuating hepatic stellate cell activation via the TLR4/MyD88/NF-κB signaling pathway. *Sci. Rep.* 5:18038 10.1038/srep18038 PMC4674875 26655640

[B14] Na K. Y. Bacchini P. Bertoni F. Kim Y. W. Park Y. K. (2012). Syndecan-4 and fibronectin in osteosarcoma. *Pathology* 44 325–330. 10.1097/PAT.0b013e328353447b 22531343

[B15] Rockey D. C. (2001). Cellular pathophysiology of portal hypertension and prospects for management with gene therapy. *Clin. Liver Dis.* 5 851–865. 10.1016/S1089-3261(05)70195-1 11565144

[B16] Sokar S. S. El-Sayad M. E. Ghoneim M. E. Shebl A. M. (2017). Combination of Sitagliptin and Silymarin ameliorates liver fibrosis induced by carbon tetrachloride in rats. *Biomed. Pharmacother.* 89 98–107. 10.1016/j.biopha.2017.02.010 28222401

[B17] Song Y. McFarland D. C. Velleman S. G. (2012). Syndecan-4 cytoplasmic domain regulation of turkey satellite cell focal adhesions and apoptosis. *Mol. Biol. Rep.* 39 8251–8264. 10.1007/s11033-012-1673-1 22660841

[B18] Sun J. Luo Q. Liu L. Yang X. Zhu S. Song G. (2017). Salinomycin attenuates liver cancer stem cell motility by enhancing cell stiffness and increasing F-actin formation via the FAK-ERK1/2 signalling pathway. *Toxicology* 384 1–10. 10.1016/j.tox.2017.04.006 28395993

[B19] Trautwein C. Friedman S. L. Schuppan D. Pinzani M. (2015). Hepatic fibrosis: concept to treatment. *J. Hepatol.* 62 S15–S24. 10.1016/j.jhep.2015.02.039 25920084

[B20] Tsukada S. Parsons C. J. Rippe R. A. (2006). Mechanisms of liver fibrosis. *Clin. Chim. Acta* 364 33–60. 10.1016/j.cca.2005.06.014 16139830

[B21] Wang N. Xu Q. Tan H. Y. Hong M. Li S. Yuen M. F. (2016). Berberine inhibition of fibrogenesis in a rat model of liver fibrosis and in hepatic stellate cells. *Evid. Based Complement. Alternat. Med.* 2016:8762345 10.1155/2016/8762345 PMC4867075 27239214

[B22] Wang T. Chen H. Lv K. Ji G. Zhang Y. Wang Y. (2017). iTRAQ-based proteomics analysis of hippocampus in spatial memory deficiency rats induced by simulated microgravity. *J. Proteomics* 160 64–73. 10.1016/j.jprot.2017.03.013 28341594

[B23] Wang Y. Wu N. Pang B. Tong D. Sun D. Sun H. (2017). TRIB1 promotes colorectal cancer cell migration and invasion through activation MMP-2 via FAK/Src and ERK pathways. *Oncotarget* 8 47931–47942. 10.18632/oncotarget.18201 28624785 PMC5564616

[B24] Wilcox-Adelman S. A. Denhez F. Goetinck P. F. (2002). Syndecan-4 modulates focal adhesion kinase phosphorylation. *J. Biol. Chem.* 277 32970–32977. 10.1074/jbc.M201283200 12087088

[B25] Wu Y. C. Jhao Y. T. Cheng Y. C. Chen Y. (2017). 15-Deoxy-Δ^12,14^-prostaglandin J_2_ inhibits migration of human thyroid carcinoma cells by disrupting focal adhesion complex and adherens junction. *Oncol. Lett.* 13 2569–2576. 28454435 10.3892/ol.2017.5773 PMC5403263

[B26] Xu L. N. Wei Y. L. Peng J. Y. (2015). Advances in study of dioscin–a natural product. *Zhongguo Zhong Yao Za Zhi* 40 36–41. 25993784

[B27] Yang L. Miura K. Zhang B. Matsushita H. Yang Y. M. Liang S. (2017). TRIF differentially regulates hepatic steatosis and inflammation/fibrosis in mice. *Cell Mol. Gastroenterol. Hepatol.* 3 469–483. 10.1016/j.jcmgh.2016.12.004 28462384 PMC5403956

[B28] Yin L. H. Xu L. N. Wang X. N. Lu B. N. Liu Y. T. Peng J. Y. (2010). An economical method for isolation of dioscin from *Dioscorea nipponica* Makino by HSCCC coupled with ELSD, and a computer-aided UNIFAC mathematical model. *Chromatographia* 71 15–23. 10.1365/s10337-009-1407-2

[B29] Zhang X. L. Han X. Yin L. H. Xu L. N. Qi Y. Xu Y. W. (2015). Potent effects of dioscin against liver fibrosis. *Sci. Rep.* 5:9713 10.1038/srep09713 PMC4389718 25853178

[B30] Zhao X. Wang L. Zhang H. Zhang D. Zhang Z. Zhang J. (2017). Protective effect of artemisinin on chronic alcohol induced-liver damage in mice. *Environ. Toxicol. Pharmacol.* 52 221–226. 10.1016/j.etap.2017.04.008 28448816

[B31] Zhao X. K. Yu L. Cheng M. L. Che P. L. Lu Y. Y. Zhang Q. (2017). Focal adhesion kinase regulates hepatic stellate cell activation and liver fibrosis. *Sci. Rep.* 7:4032 10.1038/s41598-017-04317-0 PMC5481439 28642549

[B32] Zou C. Luo Q. Qin J. Shi Y. Yang L. Ju B. (2013). Osteopontin promotes mesenchymal stem cell migration and lessens cell stiffness via integrin beta1, FAK, and ERK pathways. *Cell Biochem. Biophys.* 65 455–462. 10.1007/s12013-012-9449-8 23086356

