# Corrigendum: Dioscin Inhibits HSC-T6 Cell Migration *via* Adjusting SDC-4 Expression: Insights From iTRAQ-Based Quantitative Proteomics

**DOI:** 10.3389/fphar.2019.01036

**Published:** 2019-09-18

**Authors:** Lianhong Yin, Yan Qi, Youwei Xu, Lina Xu, Xu Han, Xufeng Tao, Shasha Song, Jinyong Peng

**Affiliations:** College of Pharmacy, Dalian Medical University, Dalian, China

**Keywords:** cell migration, dioscin, hepatic stellate cells, iTRAQ, liver fibrosis, syndecan-4

In the original article, there was a mistake in **Figure 6A** as published. The authors inserted the wrong image in this figure part. In this corrigendum, we have furthermore provided the correct result of the shRNA+ dio 5.0 μg/mL group, which showed no influence on the results. The corrected **Figure 6** appears below. The authors apologize for this error and state that this does not change the scientific conclusions of the article in any way. The original article has been updated.

**Figure 6:**
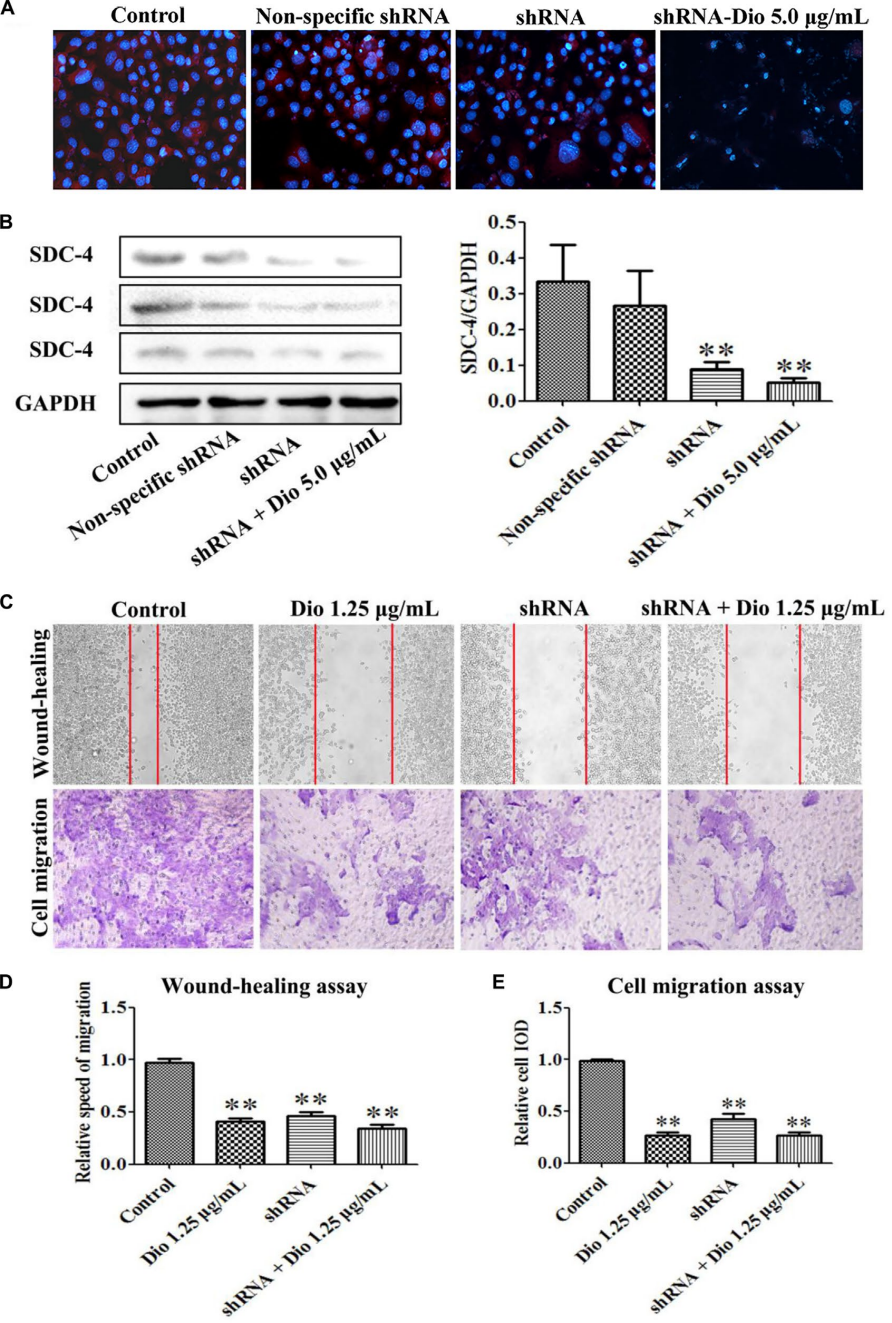
Effects of SDC-4 shRNA and dioscin on SDC-4 expression. Effects of dioscin on SDC-4 level based on immunofluorescence assay (× 400 original magnification) **(A)** and western blotting assay **(B)** in HSC-T6 cells. **(C–E)** Wound-healing and cell migration assays of HSC-T6 cells treated by dioscin or SDC-4 shRNA. Data are presented as mean ± SD (*n* = 3). *p < 0.05 and **p < 0.01 compared with control group.

