# Supplementary material for: Dioscin Inhibits HSC-T6 Cell Migration via Adjusting SDC-4 Expression: Insights from iTRAQ-Based Quantitative Proteomics
Source: Front Pharmacol. 2017 Sep 20;8:665. doi: 10.3389/fphar.2017.00665 (PMC5627034; doi:10.3389/fphar.2017.00665)
Supplement: Supplementary file 1 [file Presentation_1.PDF]

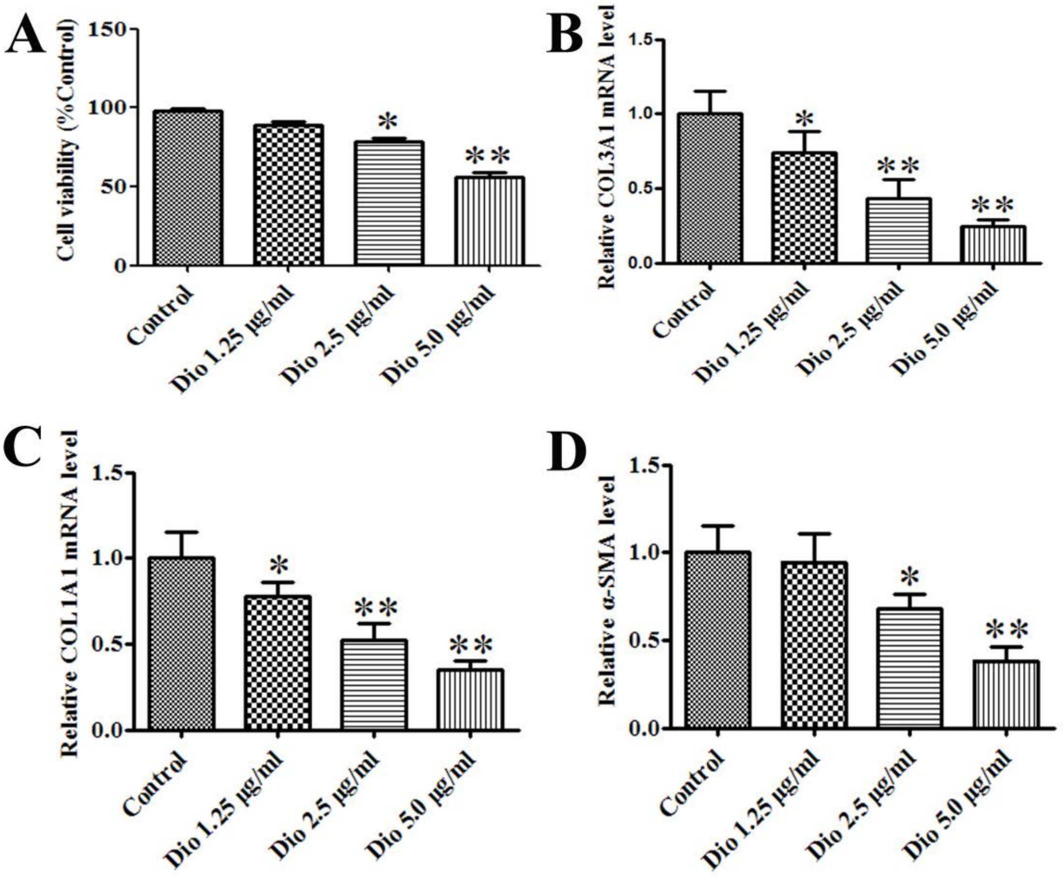

Supplementary Figure 1. Effect of dioscin on the activated HSC-T6 cells. (A) Effect of dioscin on cell viability. (B) Effect of dioscin on COL3A1 in HSC-T6 cells. (C) Effect of dioscin on COL1A1 in HSC-T6 cells. (D) Effect of dioscin on COL3A1 in HSC-T6 cells. * p < 0.05 and ** p < 0.01 compared with control group.

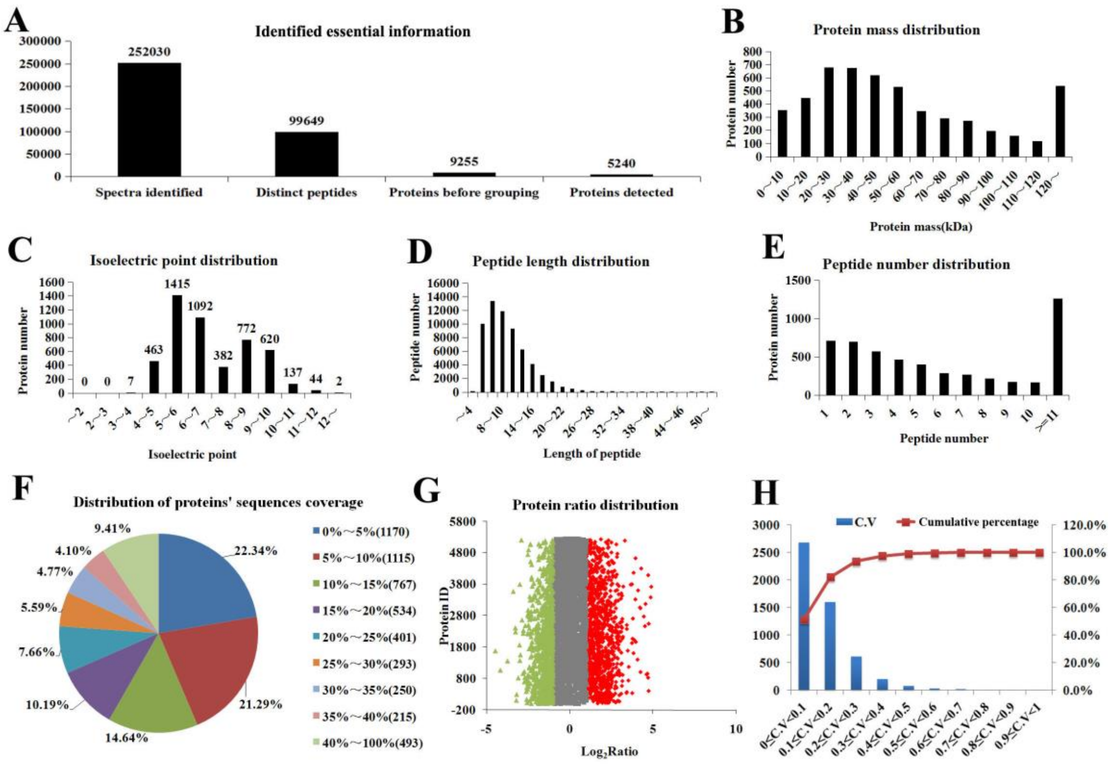

Supplementary Figure 2. Overall analysis of iTRAQ quantification. (**A**) Identified essential information. (**B**) Protein mass distribution, X-axis is the molecular mass of the identified proteins (Units: 1000 Dalton, kDa), Y-axis is the number of proteins identified. (**C**) Isoelectric point distribution, X-axis is the isoelectric point of the identified proteins (Units: 1000 Dalton, kDa), Y-axis is the number of proteins identified. (**D**) Peptide length distribution indicates the percentage of peptides with different lengths in all peptides, X-axis is the amino acid residue of peptide segment, Y-axis is the number of the length of peptide segment. (**E**) Peptide number distribution, X-axis is the number of peptides in identified proteins, Y-axis is the number of proteins. The trend shown in the figure shows that the majority of the identified proteins contains less than 10 peptides, and the number of proteins decreases with the number of matching peptides. (**F**) Distribution of protein's sequences coverage, different colors represent different sequence coverage range, and the pie chart shows the percentage of protein with different coverage range in the total protein number. (**G**) Protein ratio distribution, X-axis is 2 fold differences after logarithmic transformation after the base value. An expression of more than o is up-regulated and less than 0 is down-regulated. The points with multiple differences

greater than 1 are marked in red and green (red for up-regulated, green for down-regulated). (H) Coefficient of variation (C.V) distribution histogram, C.V=50%, cumulative percentage was up to 96%.

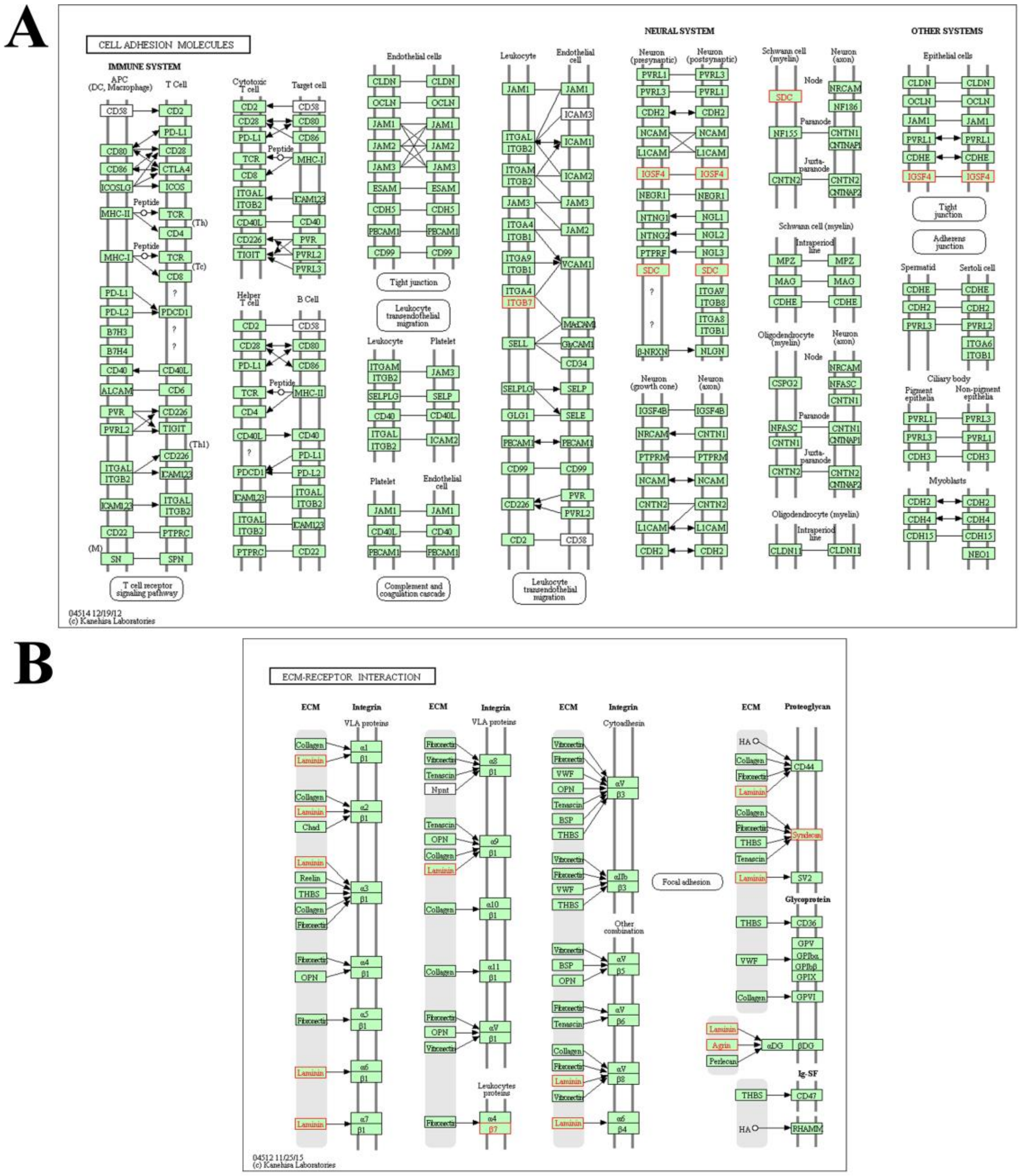

Supplementary Figure 3. Pathway analysis using the KEGG database. (**A**) Cell adhesion molecules (CAMs, rno04514). (**B**) ECM-receptor interaction (rno04512). Proteins with red shading were differentially expressed.

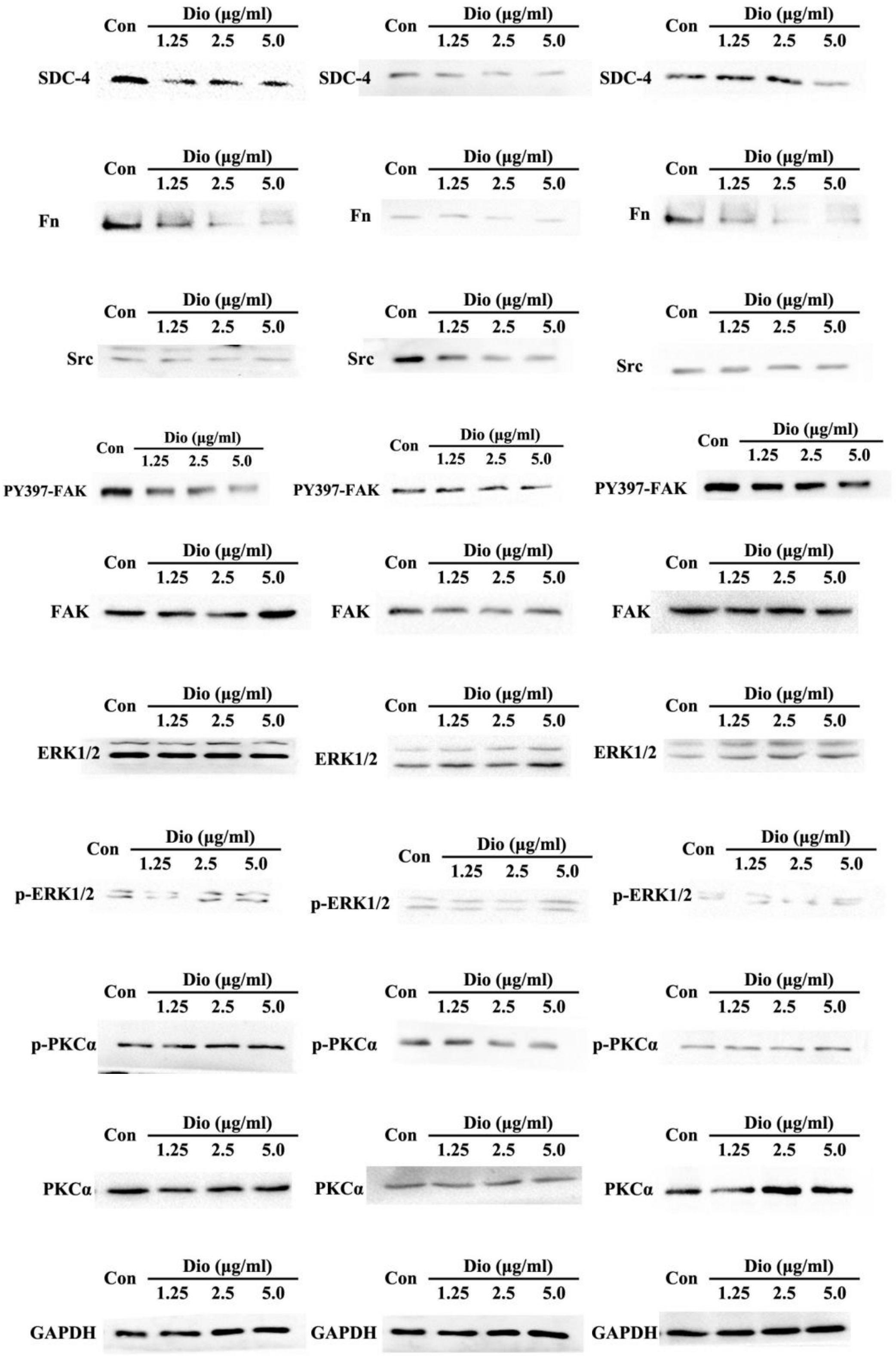

Supplementary Figure 4. Dioscin inhibited HSC-T6 adhesion and migration via regulating SDC-4 signal pathway, including SDC-4, Fn, Src, FAK, PY397-FAK, ERK1/2, p-ERK1/2, p-PKCα, PKCα. Each protein has three repeats.

Supplementary Table 1 The deferentially expressed proteins (fold change ≥ 2.0 and *p-value* < 0.05)

No.	Name	Dioscin vs. Control		
		Mean	SD	*p-value*
1	Calcyclin-binding protein	25.74863551	10.81835591	2.31504E-06
2	E3 ubiquitin-protein ligase UHRF1	19.93821409	6.942157195	2.3118E-06
3	Protein Anlnl1 (Fragment)	15.06795327	5.787662772	7.74662E-06
4	Coiled-coil domain-containing protein 117	10.57224278	0.34143153	9.85948E-09
5	Protein Ube2c	10.31581001	2.939218851	9.52001E-06
6	Stathmin	9.932960188	2.052779311	3.84468E-06
7	DNA topoisomerase 2 (Fragment)	9.49145483	1.541703437	2.09236E-06
8	Peptidyl-prolyl cis-trans isomerase A	9.24649312	1.046073018	5.71601E-07
9	Nuclear autoantigenic sperm protein	11.17864256	6.283589383	0.000112759
10	Protein S100-A6	8.19668461	0.821782372	7.64651E-07
11	Hepatoma-derived growth factor-related protein 2	8.406748865	2.070145826	1.03038E-05
12	m7GpppX diphosphatase	8.047236618	0.506033109	2.01236E-07
13	Protein phosphatase 1 regulatory subunit 7	8.111468353	1.681673638	7.87304E-06
14	Retinoic acid receptor RXR-beta	13.04770886	9.75114214	0.000429
15	DnaJ (Hsp40) homolog, subfamily C, member 9 (Predicted)	7.663488092	1.833370087	1.50557E-05
16	Polymerase I and transcript release factor	7.192592837	1.103301597	4.73572E-06
17	DNA primase large subunit	7.020577297	0.335801421	1.39053E-07
18	Peptidyl-prolyl cis-trans isomerase	6.964767215	0.519113509	5.45707E-07
19	Phosphoserine aminotransferase	6.690734352	0.361286988	2.34149E-07
20	Transcription elongation factor A protein 1	6.700484475	0.551007975	8.54063E-07
21	Transgelin-2	6.766411997	1.522533447	1.87364E-05
22	Protein Spag7	7.073060931	2.514020733	7.81697E-05
23	Aspartate aminotransferase, cytoplasmic	6.871877577	2.494148042	9.42945E-05
24	Poly [ADP-ribose] polymerase 1	6.821881036	2.51742974	9.609E-05
25	Heterogeneous nuclear ribonucleoprotein A1	6.602173013	2.196581698	7.68596E-05
26	Proliferating cell nuclear antigen	6.212171882	1.210563514	1.62035E-05
27	Elongation factor 2	5.973517768	0.224536686	1.19453E-07
28	Importin subunit alpha	6.272228763	1.657221812	4.17935E-05
29	Kif20a protein	5.922410971	0.858488167	8.17211E-06

30	Protein Wdhd1	5.84354848	0.598284681	2.44226E-06
31	DNA polymerase	6.177815276	1.83348342	6.61728E-05
32	Cyclin-dependent kinase 1	5.770012532	0.188301644	9.63162E-08
33	Protein Spc24	6.415437508	2.376072906	0.000128774
34	Thioredoxin reductase 1, cytoplasmic	5.755618647	0.136866216	3.45169E-08
35	Protein RGD1310335	5.772229263	0.523833811	1.93812E-06
36	Hematological and neurological expressed 1-like protein	5.977716155	1.496347311	4.46883E-05
37	Isoform 2 of Tropomyosin beta chain	5.659079804	0.382962735	8.55198E-07
38	Histone-binding protein RBBP7	5.926647978	1.503842932	4.98692E-05
39	Protein RTF2 homolog	5.940169011	1.595736606	4.98256E-05
40	Phosphatidylethanolamine-binding protein 1	5.686830482	0.751341747	6.6212E-06
41	PCNA-associated factor	5.616009338	0.288349451	3.75584E-07
42	Protein Ipo11	5.738465463	1.0897627	1.83594E-05
43	Multifunctional protein ADE2	6.1129378	2.159811797	0.000132856
44	Bleomycin hydrolase	5.547878118	0.634834632	4.41298E-06
45	Histone acetyltransferase type B catalytic subunit	5.760649458	1.551889135	5.23457E-05
46	Protein Zfp598	5.89854242	1.858598781	9.67214E-05
47	Basic transcription factor 3	5.578765852	1.116159579	2.56362E-05
48	Protein Rprd1b	5.724980961	1.582366331	6.7836E-05
49	Protein Cnn2	5.519168611	0.952508704	1.69931E-05
50	Protein LOC100911431	5.406351116	0.407794362	1.25409E-06
51	Sorbin and SH3 domain-containing protein 1 (Fragment)	8.4022961	5.89064162	0.001162087
52	Protein Erh (Fragment)	5.392728053	0.703315869	8.04256E-06
53	Zinc finger CCCH domain-containing protein 15	5.761035426	1.850395651	0.000115105
54	Uncharacterized protein (Fragment)	5.304790588	0.676471817	7.54499E-06
55	Mitogen-activated protein kinase kinase kinase kinase 3	10.75648653	9.218569724	0.002057263
56	Deoxyuridine 5'-triphosphate nucleotidohydrolase	5.423459932	1.349012888	5.75901E-05
57	Protein Rybp (Fragment)	6.287339472	3.1407893	0.000467682
58	Protein Tpx2	5.397887599	1.338735216	5.81467E-05
59	Dlgap5 protein	5.163657677	0.395860016	1.74865E-06
60	Protein Pus7	5.632651557	2.012447663	0.000171701
61	Uncharacterized protein	5.143033066	0.444465103	2.55385E-06

62	Protein Ppm1g	5.239997995	0.947503361	2.25113E-05
63	Phosphoserine phosphatase	5.451731903	1.757097915	0.000139519
64	D-tyrosyl-tRNA(Tyr) deacylase	5.553642102	1.965793188	0.00019464
65	Cbx5 protein	5.187514859	1.138050017	4.48533E-05
66	Protein LOC100911374 (Fragment)	5.231606691	1.328989712	7.41092E-05
67	Spectrin alpha chain, non-erythrocytic 1	5.045177632	0.668302924	1.01622E-05
68	Protein LOC100359539	5.12032166	0.999667341	3.28529E-05
69	Small glutamine-rich tetratricopeptide repeat-containing protein alpha	4.954696661	0.391280489	2.2333E-06
70	Protein Arhgdib	5.106076283	1.130351434	5.1272E-05
71	Nuclear ubiquitous casein and cyclin-dependent kinase substrate 1	6.416670331	3.581396807	0.000859073
72	Protein Mcm4	4.949643082	0.485445577	4.42337E-06
73	Uncharacterized protein (Fragment)	5.596020287	2.283283251	0.000314387
74	Stress-induced-phosphoprotein 1	4.94989631	0.622146349	9.47232E-06
75	Delta-aminolevulinic acid dehydratase	6.372488461	3.56982841	0.000824665
76	Hypoxanthine-guanine phosphoribosyltransferase	4.885530659	0.365424316	1.98552E-06
77	Lamina-associated polypeptide 2, isoform beta	4.967278649	0.879758282	2.63458E-05
78	Inosine-5'-monophosphate dehydrogenase 2	4.902851924	0.598483843	9.00613E-06
79	PC4 and SFRS1 interacting protein 1	4.834261835	0.216714633	4.59683E-07
80	Isochorismatase domain-containing protein 1	5.557553525	2.340385976	0.00036966
81	SUMO-conjugating enzyme UBC9	4.955769571	0.959397601	3.52522E-05
82	Protein Zfp593	6.747720232	4.190394943	0.001183311
83	Protein Ranbp1	5.010193181	1.220973981	7.23503E-05
84	Protein Kif11	4.950354636	1.193949261	7.80512E-05
85	Api5 protein	4.781079694	0.594929522	1.01462E-05
86	Protein LOC100911856	4.781302599	0.598061564	1.02638E-05
87	Protein Pin4	4.856521853	0.954763857	4.06808E-05
88	Protein Ppidl1	4.74662205	0.434798681	4.36536E-06
89	Protein Ppidl1	4.74662205	0.434798681	4.36536E-06
90	RNA-binding protein 8A	5.040562154	1.485424226	0.000134397
91	Chromobox homolog 3 (HP1 gamma homolog, Drosophila)	4.872140844	1.179437025	7.95239E-05
92	Adenylosuccinate synthetase isozyme 2	4.696064171	0.483758678	6.14851E-06
93	Phosphoribosylglycinamide formyltransferase, isoform CRA_a	4.698537619	0.636485429	1.35534E-05

94	Lupus La protein homolog	4.647304188	0.398812664	3.66554E-06
95	Translationally-controlled tumor protein	4.608259056	0.264188478	1.12638E-06
96	Protein Rsu1	4.674038268	0.787769669	2.94654E-05
97	Plasminogen activator inhibitor 1 RNA-binding protein	4.576579699	0.262634218	1.1938E-06
98	Protein LTV1 homolog	4.721409185	1.132693622	7.05016E-05
99	Ddx27 protein	5.20107923	2.174165386	0.000462527
100	Glucose-6-phosphate isomerase	4.986331299	1.828695694	0.000309309
101	Protein LOC100909901	4.65286873	1.001847228	6.77952E-05
102	Periplakin (Predicted)	4.655236908	1.02166873	7.0926E-05
103	EBNA1 binding protein 2	4.738036271	1.298802171	0.000135302
104	Eukaryotic translation initiation factor 3 subunit J	4.486776896	0.340097052	2.84282E-06
105	Protein Cks1b	5.702266382	3.064910727	0.001009018
106	1-phosphatidylinositol 4,5-bisphosphate phosphodiesterase beta-3	4.507225141	0.697748558	2.48171E-05
107	DNA replication licensing factor MCM6	4.436451446	0.353759569	3.50657E-06
108	Heat shock protein 105 kDa	4.448487805	0.51790283	1.10229E-05
109	Dead end homolog 1 (Zebrafish)	4.463804929	0.676306095	2.31868E-05
110	Glyoxalase domain-containing protein 4	4.527601142	0.925156028	5.97556E-05
111	DNA ligase (Fragment)	4.405864391	0.349151039	3.63619E-06
112	Protein RGD1564148	4.388329861	0.18805307	5.60571E-07
113	Protein G3bp1	4.356996017	0.147385934	2.88866E-07
114	Thimet oligopeptidase	4.383328131	0.461328621	8.5629E-06
115	Eukaryotic translation initiation factor 4H	4.412891477	0.659678011	2.73003E-05
116	Alpha-actinin-1	4.340742978	0.424756732	7.11943E-06
117	Protein kinase C delta-binding protein	4.442935433	0.888890836	6.72713E-05
118	Inositol-3-phosphate synthase 1	4.504542962	1.140581692	0.000124213
119	Protein Rbm6	4.526418711	1.207633749	0.000146419
120	Glutathione S-transferase omega-1	4.287214169	0.140808937	2.72213E-07
121	28 kDa heat- and acid-stable phosphoprotein	4.276158092	0.343578886	4.14214E-06
122	Peptidyl-prolyl cis-trans isomerase FKBP4	4.248446709	0.159693549	4.28824E-07
123	14-3-3 protein beta/alpha	4.316496406	0.686949043	3.17494E-05
124	Transforming protein RhoA	4.254399098	0.561576318	1.9334E-05
125	H2-K region expressed gene 2, rat orthologue	4.234533134	0.555424854	1.93118E-05

126	Protein Hells	4.196511406	0.309871535	3.45162E-06
127	Eukaryotic translation elongation factor 1 beta 2	4.192499698	0.51526016	1.64276E-05
128	Pleiotropic regulator 1	4.434006788	1.335386973	0.000240791
129	Eukaryotic translation initiation factor 4B	4.140818346	0.095035677	1.09828E-07
130	A disintegrin and metalloprotease domain 8 (Predicted), isoform CRA_b	5.489777014	3.186077371	0.001669344
131	Naca protein	4.702591345	1.906923758	0.000592026
132	Protein Smarcc1	4.15161785	0.484116785	1.45205E-05
133	Nuclear migration protein nudC	4.103029646	0.103841805	1.51695E-07
134	Protein Ipo5	4.127430741	0.419175167	9.72438E-06
135	Glycylpeptide N-tetradecanoyltransferase 1	4.189011952	0.742119846	5.08454E-05
136	Isoform 2 of Elongation factor 1-delta	4.088783478	0.247518206	2.05456E-06
137	Phosphatidylinositol transfer protein alpha isoform	4.115628543	0.501968657	1.7266E-05
138	NEFA-interacting nuclear protein NIP30	4.368060697	1.380221034	0.000302878
139	Protein Myo18a	4.062397579	0.281326412	3.23229E-06
140	Protein Smtn	4.263115538	1.139914991	0.000186471
141	Eukaryotic translation initiation factor 5	4.151077213	0.801097449	7.17315E-05
142	EF-hand domain-containing protein D2	4.035011824	0.288662006	3.89158E-06
143	Protein Sap30bp	4.074745785	0.563127865	2.59442E-05
144	Craniofacial development protein 1	4.751876295	2.197990514	0.001002053
145	NSFL1 cofactor p47	3.995017782	0.225192159	1.86559E-06
146	Condensin complex subunit 2 (Fragment)	4.088730176	0.755771727	6.52416E-05
147	Polymerase (DNA-directed), delta interacting protein 3 (Predicted), isoform CRA_a	3.982374699	0.332398725	6.28809E-06
148	Acylphosphatase	3.97095344	0.293268411	4.4233E-06
149	Glia maturation factor beta	3.99262874	0.565188205	3.01156E-05
150	Peroxiredoxin-6	4.261284529	1.371164505	0.000360964
151	Ubiquitin-conjugating enzyme E2 variant 2	3.93165479	0.233562424	2.31737E-06
152	Importin 9 (Predicted)	3.973077949	0.550184015	2.94549E-05
153	Coactosin-like protein	3.944900257	0.43987805	1.55807E-05
154	Parathymosin	4.064384746	0.952183417	0.000144563
155	Protein Sephs1	4.146196468	1.181923851	0.000264881
156	Protein Dda1	4.368390576	1.732396723	0.000660883

157	Isoform 4 of Protein kinase C and casein kinase substrate in neurons 2 protein	3.999583885	0.763596808	7.91387E-05
158	Nucleoside diphosphate kinase B	4.346762541	1.629427859	0.000593224
159	GTPase activating protein (SH3 domain) binding protein 2	3.888190826	0.262824836	3.65603E-06
160	DEAD (Asp-Glu-Ala-Asp) box polypeptide 42 (Predicted)	3.894489329	0.367786879	9.6887E-06
161	Protein DEK	4.667956505	2.238940112	0.001264754
162	Transaldolase	3.866735661	0.177898637	1.13846E-06
163	Yorkie homolog	4.302605453	1.612452996	0.000600168
164	LSM3 homolog, U6 small nuclear RNA associated (S. cerevisiae) (Predicted)	3.869917796	0.253469839	3.38035E-06
165	Protein Smek2	4.525766488	2.028435372	0.001080667
166	N-acetyltransferase 5 (ARD1 homolog, S. cerevisiae)	4.030260094	1.018246908	0.000172467
167	Proteasome subunit beta type	3.96437004	0.828834667	0.000108596
168	SAP domain-containing ribonucleoprotein	3.894789757	0.567271528	3.74633E-05
169	Protein Rdbp	4.389568189	1.834014077	0.000883097
170	Protein Wibg	3.845073795	0.414712073	1.57887E-05
171	LOC681996 protein	3.812140447	0.122555625	4.19586E-07
172	Protein Bclaf1	3.834406295	0.391503708	1.32859E-05
173	Isoform 2 of Microtubule-associated protein 4	3.805971769	0.201663572	2.02695E-06
174	Adaptor protein complex AP-1, sigma 1 (Predicted), isoform CRA_b	3.843813139	0.499265822	2.76055E-05
175	Protein Mcm2	3.971135589	0.969118611	0.000182047
176	Phosducin-like protein	3.922406487	0.838233188	0.000123451
177	Mammary tumor virus receptor 2, isoform CRA_a	3.785108648	0.08298036	1.34635E-07
178	Hsp90 co-chaperone Cdc37	3.808531637	0.394678506	1.45652E-05
179	Transitional endoplasmic reticulum ATPase	3.791854555	0.274179354	4.86331E-06
180	Ribonucleoside-diphosphate reductase	3.80675306	0.558652004	4.35634E-05
181	Nucleolin	4.454803958	2.078370503	0.001337118
182	Protein Mylk	3.814675984	0.630688126	5.96349E-05
183	Lamin A, isoform CRA_b	3.815535187	0.634768132	6.17877E-05
184	Far upstream element-binding protein 2	3.83434074	0.712318037	8.60166E-05
185	Protein IWS1 homolog	3.749141947	0.283011297	5.87671E-06
186	Vinculin	3.736064472	0.188391672	1.73863E-06

187	Cyclin-dependent kinase 4	3.726871744	0.171464064	1.32286E-06
188	Heterogeneous nuclear ribonucleoproteins A2/B1	3.821106378	0.743462149	0.00010113
189	Protein Heatr3	3.738384836	0.37926402	1.47508E-05
190	Spindle and kinetochore-associated protein 2	3.742877039	0.524970983	3.90665E-05
191	Protein Kif23	3.726093021	0.439214949	2.27568E-05
192	Malate dehydrogenase, cytoplasmic	4.303330409	1.893774436	0.001174761
193	Acidic leucine-rich nuclear phosphoprotein 32 family member A	3.756763436	0.642649591	7.18765E-05
194	14-3-3 protein zeta/delta	3.954852363	1.221805761	0.000421886
195	Phosphoglycerate mutase 1	3.790274766	0.819975304	0.000150516
196	Glutathione peroxidase 1	3.812976052	0.899810441	0.000192483
197	Protein Rfc1	3.983577239	1.389875189	0.000525976
198	Protein Ppp2r4	4.10800063	1.584377836	0.000833292
199	Glutaredoxin-3	3.749380745	0.712399017	0.000101384
200	Protein LOC100912917	3.666197308	0.316045489	9.72549E-06
201	Protein AATF	4.770292249	2.68987609	0.002605133
202	Protein Numa1 (Fragment)	3.654699649	0.264262105	5.65759E-06
203	Protein Ube2e3	3.922297568	1.262087445	0.00050499
204	Leukotriene A-4 hydrolase	3.618736681	0.211491736	3.09556E-06
205	Four and a half LIM domains 3 (Predicted)	3.740212397	0.816317104	0.000160557
206	Large proline-rich protein BAG6	3.708012179	0.720255278	0.000111733
207	Myosin, heavy polypeptide 9, non-muscle	3.607421744	0.12545388	6.54342E-07
208	Clustered mitochondria protein homolog	3.867093901	1.16821072	0.000425922
209	Cellular tumor antigen p53	3.601680175	0.200002613	2.76726E-06
210	Eukaryotic translation initiation factor 5A-1	3.660898706	0.610646387	7.45723E-05
211	Amidophosphoribosyltransferase	3.630780374	0.491239286	3.95168E-05
212	Heat shock 70 kDa protein 4	3.721430714	0.853096109	0.000198345
213	Menage a trois 1	3.677526765	0.743459963	0.000120651
214	Protein Sptbn1	3.577377836	0.350644536	1.56037E-05
215	Protein Diaph3 (Fragment)	3.71751701	0.921485666	0.0002549
216	DNA polymerase	3.697214619	0.909573927	0.000250476
217	DNA replication complex GINS protein SLD5	4.922291449	3.030002797	0.00380147
218	Eukaryotic translation initiation factor 3 subunit C	3.568519527	0.440573223	3.20463E-05

219	Activator of basal transcription 1	5.150044193	3.445519121	0.005374605
220	Protein LOC100912106	3.551328652	0.429412619	3.06648E-05
221	Protein Hnrpll	4.178244389	1.938482848	0.001685218
222	Protein Hypk (Fragment)	3.600108605	0.703446283	0.000132268
223	General transcription factor IIF subunit 1	3.584887859	0.653428213	0.000107955
224	Chondroitin sulfate proteoglycan 6, isoform CRA_b	3.496546262	0.219145614	4.37559E-06
225	Kinesin-like protein KIF15	3.748748649	1.202566667	0.000569298
226	Kinesin-like protein KIF15	3.748748649	1.202566667	0.000569298
227	Serine/threonine-protein kinase Chk1	3.503985837	0.439398637	3.63016E-05
228	Protein Ncapg (Fragment)	3.656718415	0.997574859	0.000365116
229	Protein Tsr1	3.546849312	0.667974675	0.000124138
230	Dual specificity protein phosphatase	5.830835371	4.323115051	0.007098636
231	SUMO-activating enzyme subunit 1	3.492400924	0.482009447	4.95926E-05
232	Cullin-associated NEDD8-dissociated protein 1	3.49454006	0.502309361	5.61542E-05
233	UV excision repair protein RAD23 homolog B	3.525368124	0.68388419	0.000139415
234	Protein Uba2 (Fragment)	3.429671948	0.135312694	1.16561E-06
235	Elongation factor 1-gamma	3.763632072	1.335212801	0.000866287
236	Glycolipid transfer protein	3.483600883	0.548372802	7.2349E-05
237	Protein Ccdc137	3.425925655	0.236672249	6.45852E-06
238	NudC domain containing 1	3.43740555	0.403634413	3.0995E-05
239	Caspase-3	3.528942214	0.818210538	0.000234441
240	Protein Ube3a	3.416918219	0.343261184	1.97667E-05
241	Protein LOC685520	3.688493493	1.249906388	0.000734473
242	Transcription intermediary factor 1-beta	3.365996881	0.088739706	3.90105E-07
243	Histocompatibility (Minor) HA-1 (Predicted), isoform CRA_b	3.908247788	1.721726341	0.001768873
244	Protein Cit	3.46869566	0.7751991	0.000237985
245	Inosine-5'-monophosphate dehydrogenase 1	3.425657358	0.692920905	0.000179297
246	Cytosine-specific methyltransferase	3.31988796	0.09156472	4.56277E-07
247	Chromosome segregation 1-like (S. cerevisiae) (Predicted)	3.368440263	0.487883552	6.72182E-05
248	Adenylosuccinate lyase (Predicted)	3.317634923	0.23655755	8.15302E-06
249	Protein Prrc2c	3.318099935	0.24417579	9.29504E-06
250	Protein Cobll1 (Fragment)	3.339843433	0.504092464	7.82073E-05

251	Calcium binding protein 39 (Predicted), isoform CRA_a	3.330425981	0.487086604	7.42698E-05
252	Polymerase (RNA) II (DNA directed) polypeptide D (Predicted)	3.305351285	0.384525245	3.72394E-05
253	COP9 signalosome complex subunit 4	3.359817262	0.656975744	0.000176534
254	Rho GDP-dissociation inhibitor 1	3.370546691	0.726470477	0.000239341
255	Caprin-1	3.264189876	0.340292281	2.78234E-05
256	Ubiquitin-fold modifier-conjugating enzyme 1	3.404420574	0.883018145	0.000409692
257	Eukaryotic translation initiation factor 3 subunit A	3.240089051	0.189362049	4.94722E-06
258	Protein Gins3	3.240913153	0.207315621	6.49342E-06
259	Microtubule-associated protein RP/EB family member 1	3.258076775	0.356585938	3.2735E-05
260	Protein Red	3.289928326	0.525470883	0.000102805
261	Microtubule-associated protein 1A	3.225358248	0.191391791	5.46072E-06
262	Protein Trim16	3.396122705	0.912618505	0.00051321
263	Glucose-6-phosphatase	3.306442571	0.648848138	0.00019296
264	U4/U6.U5 tri-snRNP-associated protein 1	3.216645393	0.29819202	2.07067E-05
265	Protein LOC100911774	3.218694744	0.32688489	2.72925E-05
266	Protein Srsf1	3.204214688	0.213149439	7.6688E-06
267	Nardilysin	3.210149073	0.311178331	2.35022E-05
268	L-lactate dehydrogenase	3.237338538	0.51660297	0.000112505
269	Proteasome activator complex subunit 2	3.313186467	0.781266911	0.000349661
270	N-alpha-acetyltransferase 25, NatB auxiliary subunit	3.195675106	0.313737232	2.48661E-05
271	Protein DJ-1	3.199897465	0.36547345	4.0289E-05
272	40S ribosomal protein S10	3.216231878	0.461625029	7.75344E-05
273	Protein LOC100909464	3.247338502	0.595061427	0.000165915
274	Prefoldin subunit 2	3.193226328	0.372683914	4.33359E-05
275	Exportin-1	3.202685804	0.468908517	8.65385E-05
276	AH receptor-interacting protein	3.159037994	0.183987614	5.56769E-06
277	Protein Kif4a	3.46716677	1.218509421	0.001218554
278	Vimentin	3.209748876	0.52934555	0.000129896
279	Ubiquitin carboxyl-terminal hydrolase	3.154194427	0.232906848	1.12916E-05
280	S-phase kinase-associated protein 1	3.189801813	0.482450978	0.000101065
281	Protein LOC100912618	3.177037693	0.432331547	7.04827E-05
282	Protein Rdx	3.436001398	1.17996518	0.001124282

283	Hepatoma-derived growth factor	3.2256762	0.645469169	0.000227139
284	Protein Iqgap3	3.127235044	0.098495189	8.71379E-07
285	Structural maintenance of chromosomes protein	3.146921947	0.33821995	3.58499E-05
286	Protein Zfp828	3.352714879	1.046796413	0.000885371
287	Eukaryotic peptide chain release factor subunit 1	3.188138975	0.587794382	0.000183203
288	Heterogeneous nuclear ribonucleoprotein K	3.59650242	1.535743249	0.002217349
289	Coiled-coil domain-containing protein 50	3.26753265	0.847000596	0.000508199
290	Isoform 2 of Poly(U)-binding-splicing factor PUF60	3.152138569	0.469342693	9.60898E-05
291	Importin 7 (Predicted), isoform CRA_c	3.10884113	0.18845747	6.62316E-06
292	Deoxythymidylate kinase (Predicted), isoform CRA_b	3.12571774	0.342764208	3.84843E-05
293	Protein Chaf1a	3.971693186	2.271014128	0.005332684
294	Hematological and neurological expressed 1 protein	3.113587009	0.27380586	2.03394E-05
295	CArG-binding factor A	3.162950397	0.557196395	0.000166932
296	Isoform 2 of General transcription factor II-I	3.130156365	0.462137066	9.82554E-05
297	Protein Snrpa1	3.103995701	0.337092142	3.87064E-05
298	Protein Map7d1	3.162217112	0.599408664	0.000221073
299	CGG triplet repeat binding protein 1 (Predicted)	3.076682898	0.06919264	3.57184E-07
300	Protein Pgp	3.269994848	0.928518743	0.000698257
301	Isoform 2 of Polypyrimidine tract-binding protein 2	3.070276916	0.10087149	1.15534E-06
302	ATP-binding cassette, sub-family E (OABP), member 1	3.18721139	0.718231463	0.00035199
303	Kinesin-like protein KIF2C	3.14745532	0.598030785	0.000213964
304	Structural maintenance of chromosomes protein	3.121492134	0.510606397	0.000137361
305	G-protein-signaling modulator 3	3.068233712	0.226559307	1.27607E-05
306	GATA zinc finger domain containing 2B	3.190468605	0.777510762	0.000453096
307	Ubiquitin-like modifier-activating enzyme 1	3.050545273	0.146811026	3.58981E-06
308	Protein Sf1	3.067813226	0.325525384	3.79174E-05
309	Nuclear pore complex protein Nup153	3.082284996	0.408412507	7.50571E-05
310	Protein Zfp259	3.303062661	1.075413152	0.001081063
311	Gelsolin	3.098295363	0.493910145	0.000128222
312	Septin-11	3.113880887	0.558428011	0.000186318
313	Ubiquilin-1	3.035467617	0.112950619	1.69147E-06
314	2'-deoxynucleoside 5'-phosphate N-hydrolase 1	3.064319808	0.367418334	5.49611E-05

315	Protein Thoc4	3.064789579	0.371608388	5.84594E-05
316	Cysteinyl-tRNA synthetase (Predicted), isoform CRA_b	3.075148162	0.471708087	0.000119931
317	Protein Abracl	3.093237198	0.561634924	0.000197843
318	Uncharacterized protein (Fragment)	3.39709333	1.351607533	0.001910516
319	Structural maintenance of chromosomes protein	3.068128786	0.471773201	0.000121122
320	Isoform 6 of Tropomyosin alpha-1 chain	3.31948356	1.177646173	0.001434662
321	Galectin-1	3.018220856	0.20541864	1.05079E-05
322	Glutathione S-transferase Yb-3	3.011196361	0.202182258	1.06652E-05
323	Nucleoside diphosphate kinase A	3.212054066	0.957345489	0.000893531
324	pre-rRNA processing protein FTSJ3	3.01963868	0.330479869	4.55543E-05
325	Protein Trmt6	3.022664022	0.364564546	6.25103E-05
326	Condensin complex subunit 1	3.057838321	0.553655322	0.000210937
327	Heat shock protein HSP 90-alpha	2.990020323	0.191930472	9.56361E-06
328	Survival of motor neuron-related-splicing factor 30	2.993037175	0.246033575	2.05866E-05
329	Ubiquitin-conjugating enzyme E2 N	2.995158338	0.279102348	2.89703E-05
330	Proteasome subunit beta type	2.983224849	0.193593582	9.78709E-06
331	Protein Rad21	3.000242097	0.344602684	5.41601E-05
332	Protein Sf3b2	2.989676356	0.298336517	3.58878E-05
333	RWD domain-containing protein 1	3.192386018	0.989124899	0.001037798
334	Enthoprotin	2.971784333	0.233796473	1.86063E-05
335	Ubiquitin-conjugating enzyme E2 Z	2.985859589	0.335800019	5.32041E-05
336	Peptidylprolyl isomerase (Cyclophilin)-like 4 (Predicted)	3.194041335	1.024084817	0.001179874
337	Protein Txlna	2.971084813	0.320068282	4.93255E-05
338	Transketolase	2.947635411	0.159166103	5.85012E-06
339	Protein Atad2	3.005201264	0.521966562	0.000186896
340	Flap endonuclease 1	2.938459851	0.07966225	7.55825E-07
341	Peptidyl-prolyl cis-trans isomerase	3.149940685	0.949329086	0.001023831
342	Protein Ranbp3	2.981384751	0.430126581	0.000114815
343	Proteasome subunit alpha type-6	2.973177039	0.416781507	0.000105224
344	Protein RGD1311703	3.262407802	1.20848096	0.001885179
345	UBX domain-containing protein 1	2.936766304	0.214561521	1.49393E-05
346	Atxn2l protein	2.977717672	0.459080168	0.000140814

347	Ubiquitin-like domain-containing CTD phosphatase 1	3.076213747	0.791574829	0.000655662
348	Isoform M2 of Pyruvate kinase PKM	2.955443521	0.37747174	7.55459E-05
349	COP9 signalosome complex subunit 1	2.941598144	0.289326491	3.75864E-05
350	Protein Taf7	3.228213153	1.158859692	0.001765946
351	Protein Srrt	2.989849	0.558263029	0.000245044
352	Protein Fam207a	2.905592545	0.110777803	2.1313E-06
353	PTPRF interacting protein, binding protein 1 (Liprin beta 1) (Predicted)	2.910579544	0.225584895	1.86787E-05
354	Catenin (Cadherin associated protein), alpha 1	2.899586645	0.131696391	3.7929E-06
355	Protein Sapcd2	2.911026387	0.325552443	5.75012E-05
356	Ras GTPase-activating protein 3	2.983805335	0.649451769	0.00043146
357	Lysine--tRNA ligase	2.871541859	0.076345726	7.92085E-07
358	Tyrosine--tRNA ligase, cytoplasmic	2.872028987	0.09778588	1.66461E-06
359	Huntingtin interacting protein 2 (Predicted), isoform CRA_a	2.916265227	0.445583305	0.000149633
360	RNA polymerase II-associated protein 3	2.959177737	0.612381587	0.000418485
361	Nudt16l1 protein	2.884833143	0.328937363	6.362E-05
362	Moesin (Fragment)	2.872823026	0.25698197	3.10803E-05
363	Nucleoredoxin (Predicted), isoform CRA_a	2.88617308	0.344140418	7.28865E-05
364	LUC7-like (S. cerevisiae)	2.996922655	0.765940426	0.000710924
365	Protein Drg1	2.861416015	0.282524106	4.15931E-05
366	Tropomyosin 1, alpha	2.849680992	0.199088404	1.50884E-05
367	Metastasis-associated gene family, member 2	2.866508384	0.344780953	7.60456E-05
368	Rab GDP dissociation inhibitor beta	2.856691164	0.30532339	5.45462E-05
369	BAG family molecular chaperone regulator 1 (Fragment)	2.849896141	0.304057121	5.15487E-05
370	Prefoldin 1 (Predicted)	2.837278737	0.210932812	1.85909E-05
371	Phosphorylated adapter RNA export protein	2.82544266	0.065265716	5.488E-07
372	Protein Soga1 (Fragment)	2.826762534	0.119282424	3.37516E-06
373	Bifunctional purine biosynthesis protein PURH	2.835181972	0.279933541	4.34064E-05
374	Eukaryotic translation initiation factor 3 subunit G	2.864930232	0.458339542	0.000177491
375	Protein Brd4	2.806504753	0.089520773	1.52251E-06
376	Protein Tpd52	2.835644481	0.360743939	9.66877E-05
377	GA repeat binding protein, alpha (Predicted)	2.81138549	0.211144412	2.00091E-05
378	DnaJ homolog subfamily C member 8	3.228903109	1.3614998	0.003383531

379	Biliverdin reductase B (Flavin reductase (NADPH))	2.973937437	0.837727486	0.001043915
380	Polyglutamine-binding protein 1	2.841651362	0.418520523	0.000154359
381	Protein NDRG2	2.803104457	0.175684146	1.16241E-05
382	Protein Larp1 (Fragment)	2.808764161	0.271194991	4.25373E-05
383	Filamin alpha	2.823663847	0.370163926	0.000108247
384	dCTP pyrophosphatase 1	2.786557567	0.057045638	4.09234E-07
385	FAS-associated factor 1	2.839687429	0.460846499	0.000201671
386	Protein Skiv2l2 (Fragment)	2.856151163	0.536947371	0.000313803
387	Valine--tRNA ligase	2.845677863	0.506069405	0.000270372
388	RNA-binding protein NOB1	2.846302953	0.508834722	0.00029382
389	14-3-3 protein epsilon	2.943910608	0.817567056	0.001035054
390	Phospholipase A-2-activating protein	2.86392957	0.590845335	0.00042866
391	Protein Rprd1a	2.864696847	0.592028171	0.000435979
392	Fam49b protein	3.084752324	1.139487204	0.002403197
393	Protein Utp14a	2.893174	0.687068213	0.000654137
394	Protein Mcm3	2.841820279	0.524103627	0.000309066
395	Na(+)/H(+) exchange regulatory cofactor NHE-RF1	2.934805851	0.807423613	0.001027083
396	Protein Thumpd1	3.248863415	1.446547754	0.004107892
397	Protein Rcc2 (Fragment)	2.885037527	0.681184899	0.000639214
398	Protein Tnks1bp1	2.775895969	0.257333436	4.03186E-05
399	Bq135360	2.806402522	0.445891424	0.000203641
400	PEST proteolytic signal-containing nuclear protein	2.793745705	0.390134123	0.000137181
401	Protein Ppp2r1a	2.861899237	0.656263979	0.000598337
402	Coiled-coil domain containing 25 (Predicted)	2.775002717	0.323781002	8.91953E-05
403	D-3-phosphoglycerate dehydrogenase	2.765511573	0.288304825	5.87932E-05
404	Nicotinamide phosphoribosyltransferase	2.79565949	0.461668479	0.00023435
405	ADP-ribosylation factor GTPase-activating protein 2	3.095061338	1.24606945	0.003271409
406	Macrophage migration inhibitory factor	2.945336702	0.917294524	0.001531911
407	Serine/threonine-protein phosphatase 1 regulatory subunit 10	2.749982977	0.248394616	3.8924E-05
408	DNA-directed RNA polymerase II subunit RPB7	2.959480307	0.937761657	0.001790754
409	Protein Tcerg1	2.803080213	0.518106331	0.000332047
410	STE20-like serine/threonine-protein kinase	2.858255862	0.708865611	0.000799631

411	Protein Ppp4r2	2.742767911	0.235733396	3.2028E-05
412	Epithelial protein lost in neoplasm	2.730764193	0.113566435	4.00352E-06
413	Gene model 672, (NCBI) (Predicted)	3.535596828	2.04057648	0.009354333
414	Protein Ppil1 (Fragment)	2.744420585	0.257575086	4.60929E-05
415	DEAH (Asp-Glu-Ala-His) box polypeptide 36 (Predicted), isoform CRA_a	2.746719815	0.286615215	6.74477E-05
416	Protein LOC100362458	2.716467874	0.014444861	8.19672E-09
417	tRNA (guanine(37)-N1)-methyltransferase	2.964666972	1.007377571	0.002104971
418	Protein Flnc	2.7171596	0.07223118	1.02512E-06
419	Clathrin light chain B	2.731616723	0.254544219	4.43504E-05
420	Proteasome subunit alpha type-4	2.74655659	0.356520947	0.000125652
421	Endophilin-B2	2.760708872	0.434396741	0.000209095
422	RNA-binding protein 10	2.762913355	0.450668965	0.000236756
423	Protein LOC100911617	2.856710866	0.764971166	0.001081351
424	Uncharacterized protein	2.704015531	0.020334698	2.36988E-08
425	Cyclin dependent kinase 2	2.705392177	0.10169246	2.96541E-06
426	Protein HEXIM1	2.706759538	0.14171505	8.27745E-06
427	Protein LOC679794	3.040954086	1.181718659	0.003143378
428	Protein Zfp428	2.801169582	0.635064317	0.000635489
429	ELAV (Embryonic lethal, abnormal vision, Drosophila)-like 1 (Hu antigen R)	2.801630425	0.631341429	0.000634035
430	Protein Tns3	2.743738652	0.44393609	0.000259627
431	RNA demethylase ALKBH5	2.798474618	0.653847258	0.000721351
432	CAP-Gly domain-containing linker protein 2	2.698189568	0.218901659	3.07104E-05
433	Phosphoglycerate kinase 1	2.789927197	0.635672295	0.000682224
434	Protein Arhgap18	2.739006362	0.456534286	0.000277454
435	Protein Nck1 (Fragment)	2.706364591	0.326088574	0.000105959
436	Proteasome subunit beta type	2.696279261	0.281161664	6.51161E-05
437	Methyl-CpG binding domain protein 3 (Predicted), isoform CRA_a	2.698284463	0.304789698	8.63091E-05
438	Heat shock protein HSP 90-beta	2.688649218	0.260154608	5.42073E-05
439	Protein Ubap2	2.688839048	0.263552251	5.56104E-05
440	Actin-like 6A	2.692261884	0.305572325	8.97863E-05
441	Acidic leucine-rich nuclear phosphoprotein 32 family member E	2.887233297	0.933195506	0.001979435

442	Calmodulin	2.672658541	0.203235432	2.63976E-05
443	Proteasome subunit alpha type-1	2.66487695	0.172208439	1.60158E-05
444	Protein Tcof1	2.690371673	0.35272746	0.000138927
445	Heterogeneous nuclear ribonucleoprotein A3	2.703124752	0.41464229	0.000217635
446	Mitogen-activated protein kinase 3	2.691366625	0.361127838	0.000155385
447	Polyadenylate-binding protein-interacting protein 2	2.83152158	0.819096608	0.001522514
448	Smu-1 suppressor of mec-8 and unc-52 homolog (C. elegans)	2.685063586	0.361866331	0.000145976
449	Protein Sin3a	2.650884724	0.130041188	7.18461E-06
450	Protein Rap1gds1	2.665872833	0.282721187	7.38064E-05
451	Polyadenylate binding protein-interacting protein 1 (Predicted)	3.23829199	1.611421435	0.007100654
452	Protein Fam98b	2.695155528	0.449399642	0.000294895
453	Proliferation-associated 2G4	2.671065253	0.341305535	0.0001348
454	Heat shock cognate 71 kDa protein	2.63685445	0.060518217	7.76078E-07
455	Isoform 4 of SWI/SNF-related matrix-associated actin-dependent regulator of chromatin subfamily E member 1	2.651300443	0.250919227	5.34442E-05
456	Ubiquitin thioesterase OTUB1	2.655003635	0.298535695	9.0127E-05
457	Protein Ppa1	2.643123579	0.218766822	3.58948E-05
458	Protein Zbtb8os-ps1	2.644504969	0.239715655	4.83967E-05
459	Filamin, beta (Predicted)	2.632077481	0.234486871	4.6866E-05
460	Minichromosome maintenance deficient 7 (S. cerevisiae)	2.64573738	0.330047604	0.000126391
461	Ddx17 protein	2.621837635	0.160602483	1.44484E-05
462	Protein Tln1	2.612514379	0.049554389	4.5178E-07
463	Dynein light chain roadblock-type 1	2.649841767	0.373295494	0.000177479
464	Golgi associated, gamma adaptin ear containing, ARF binding protein 1	2.613732833	0.10461455	4.24001E-06
465	Protein Swap70	2.703817539	0.598008909	0.000686076
466	Protein Ngdn	2.643777049	0.371078301	0.00018679
467	Protein phosphatase 1 regulatory subunit 12A	2.682243096	0.533040528	0.000517914
468	Caldesmon 1, isoform CRA_b	2.622713507	0.271361018	7.41139E-05
469	Zinc finger Ran-binding domain-containing protein 2	2.624644692	0.295071463	9.62462E-05
470	Activity-dependent neuroprotector homeobox protein	2.650031685	0.427153764	0.000285794
471	Protein Rif1	2.639730836	0.3881883	0.000219687
472	Glutamate--cysteine ligase catalytic subunit	2.640223002	0.392725024	0.000228904

473	Eukaryotic translation initiation factor 2 subunit 1	2.598829413	0.179439357	2.25554E-05
474	AMP deaminase 2	3.003383486	1.298782578	0.005221305
475	Uncharacterized protein (Fragment)	2.738751003	0.750534148	0.001442779
476	DNA polymerase alpha catalytic subunit (Fragment)	2.618318267	0.356301151	0.000172234
477	Leucine-rich repeat flightless-interacting protein 1	2.643384529	0.467875375	0.000381556
478	Importin subunit beta-1	2.68058129	0.59909089	0.000765072
479	Uncharacterized protein (Fragment)	2.608561409	0.316826305	0.000121994
480	Eukaryotic translation initiation factor 4E-binding protein 1	2.623520689	0.403487321	0.000256782
481	Hsc70-interacting protein	2.577924887	0.104896033	4.79099E-06
482	Fructose-bisphosphate aldolase	2.627581453	0.437231518	0.000322847
483	Cell division cycle 5-like protein	2.616032944	0.390121534	0.000233221
484	Testin	2.606940085	0.360733089	0.000185959
485	Minichromosome maintenance deficient 5, cell division cycle 46 (S. cerevisiae) (Predicted)	2.644329965	0.517069745	0.000526699
486	Fructose-bisphosphate aldolase A	2.584609274	0.239284531	5.58593E-05
487	Protein Rbm26	2.57612004	0.199218392	3.19684E-05
488	Protein Tbcc	2.587888893	0.281292986	9.54711E-05
489	COP9 (Constitutive photomorphogenic) homolog, subunit 6 (Arabidopsis thaliana) (Predicted), isoform CRA_a	2.570993802	0.211731032	3.99043E-05
490	Echinoderm microtubule associated protein like 4 (Predicted), isoform CRA_a	2.600567787	0.411936467	0.000284288
491	Calponin-3	2.590730455	0.37420054	0.00022124
492	MARCKS-related protein	2.591279866	0.379391988	0.000230621
493	Far upstream element-binding protein 1	2.678498749	0.68898699	0.001218324
494	Protein RGD1562502	2.559221717	0.210847887	4.15414E-05
495	Protein Hcfc1	2.547829536	0.082391435	2.52266E-06
496	Protein Rbm17	2.549069963	0.123239638	8.5225E-06
497	Cdc42-interacting protein 4	2.573197197	0.316247591	0.000138223
498	Protein Snw1	2.549774684	0.141476457	1.27813E-05
499	Glutathione S-transferase Yb-3	2.550155506	0.150457477	1.52617E-05
500	Mitogen-activated protein kinase 12	2.800407758	0.996291187	0.003060907
501	Cell division cycle-associated protein 7	2.612813843	0.504584704	0.000546249

502	Anaphase-promoting complex subunit 5	2.542661286	0.106851884	5.67534E-06
503	T-complex protein 1 subunit beta	2.6020319	0.468705654	0.000436493
504	Serine-threonine kinase receptor-associated protein	2.638935412	0.598532449	0.000883409
505	Spliceosome RNA helicase Ddx39b	2.547422897	0.209790008	4.17588E-05
506	Homeobox protein Rhox5	2.573299599	0.376077285	0.000234679
507	Proteasome (Prosome, macropain) activator subunit 1	2.601383611	0.504641015	0.000553087
508	Protein Epb4.1l2	2.532274699	0.14203369	1.34465E-05
509	COP9 signalosome complex subunit 2	2.545681068	0.267923545	9.02936E-05
510	SHC-transforming protein 1	2.605362478	0.532371917	0.000647114
511	Exosome component 7	2.847069236	1.128519586	0.004478361
512	N(G),N(G)-dimethylarginine dimethylaminohydrolase 2	2.525113298	0.10486542	5.59795E-06
513	NudC domain-containing protein 2	2.52746973	0.164017299	2.14377E-05
514	Breast carcinoma amplified sequence 2	2.540941065	0.281501667	0.00010493
515	MAD2 (Mitotic arrest deficient, homolog)-like 1 (Yeast) (Predicted), isoform CRA_a	2.530442768	0.21596041	5.07629E-05
516	Phosphatidylinositol transfer protein beta isoform (Fragment)	2.556945528	0.385463122	0.000269994
517	DCN1-like protein	2.677908286	0.767345321	0.001810856
518	Eukaryotic translation initiation factor 3 subunit L	2.535512683	0.284251568	0.00011663
519	Suppressor of G2 allele of SKP1 homolog	2.692470134	0.807751192	0.002076536
520	Histone H1.5	2.584128363	0.505955616	0.000591905
521	General transcription factor IIF subunit 2	2.657670564	0.730421487	0.001673578
522	Crk-like protein	2.621874645	0.631618603	0.001111529
523	Protein Sf3b3	2.54119288	0.346094986	0.000202435
524	Lamin-B1	2.554172492	0.413131386	0.000332593
525	Peptidyl-prolyl cis-trans isomerase FKBP1A	2.613750134	0.616809516	0.001040539
526	Centrin 2, isoform CRA_a	2.578857025	0.508798972	0.000651924
527	Glutamate--cysteine ligase regulatory subunit	2.509209228	0.143850923	1.54205E-05
528	Ubiquitin carboxyl-terminal hydrolase 15	2.652121222	0.729016668	0.001656022
529	Protein Ubqln2	2.681402385	0.81127254	0.002210097
530	Protein Ubqln4	2.865803563	1.23867282	0.005677
531	WW domain-binding protein 11	2.522184305	0.328042059	0.000183434
532	Proteasome subunit alpha type	2.53499208	0.394515013	0.000311682

533	Bromodomain-containing protein 2	2.512473061	0.284800304	0.000121167
534	Chloride channel, nucleotide-sensitive, 1A	2.501112763	0.20815713	4.90558E-05
535	Dis3 protein	2.621635111	0.701184413	0.00147933
536	Protein Ube2l3	2.504752827	0.26118966	9.33639E-05
537	Eukaryotic translation initiation factor 2 subunit 1	2.505193737	0.265654372	0.000101023
538	Ubiquitin carboxyl-terminal hydrolase	2.540179988	0.436885616	0.000424076
539	Thyroid hormone receptor-associated protein 3	2.494031828	0.185414579	3.46014E-05
540	Adapter molecule crk	2.518957298	0.353109077	0.000231487
541	Serine/threonine-protein kinase PAK 1	2.577818032	0.568557348	0.001054426
542	Protein Ubap2l	2.510329391	0.323925551	0.000183787
543	Methyltransferase like 2 (Predicted)	2.557622503	0.522193318	0.000704874
544	D4, zinc and double PHD fingers family 2 (Predicted)	2.642805218	0.764257772	0.002023544
545	Protein Cct7	2.680019386	0.85376468	0.00267502
546	Basic leucine zipper and W2 domain-containing protein 2	2.479655249	0.121505414	1.0093E-05
547	Uncharacterized protein (Fragment)	2.479655249	0.121505414	1.0093E-05
548	Beta-adducin	2.527617541	0.429790169	0.000418698
549	Beta-enolase	2.508585311	0.363342508	0.000261782
550	14-3-3 protein gamma	2.602741621	0.680631833	0.001511027
551	Focal adhesion kinase 1	2.487963719	0.264188134	0.000104069
552	LSM1 homolog, U6 small nuclear RNA associated (S. cerevisiae) (Predicted)	2.546134286	0.519195341	0.000731603
553	Formin-binding protein 1-like	2.617259493	0.729998696	0.001949272
554	Transcription elongation factor B polypeptide 2	2.491821448	0.30833713	0.000170147
555	Protein CDV3 homolog	2.49387558	0.33125039	0.000205582
556	S-adenosylmethionine synthase	2.472161005	0.200805131	4.73443E-05
557	Spectrin beta 3	2.613944986	0.738370372	0.001907031
558	Protein Smarcb1	2.462015747	0.104734987	6.43451E-06
559	Protein phosphatase methylesterase 1	2.462698715	0.124539333	1.08444E-05
560	Serine/threonine-protein phosphatase 5	2.463113209	0.134873994	1.40309E-05
561	Protein Sfpq (Fragment)	2.465867201	0.190030881	4.09248E-05
562	Serine/arginine-rich splicing factor 2	2.536055187	0.527470217	0.000791306
563	Protein Spg20	2.490934703	0.356717922	0.000266007

564	Chaperonin subunit 8 (Theta) (Predicted), isoform CRA_a	2.46026922	0.191413894	4.16306E-05
565	Scaffold attachment factor B1	2.529743768	0.524094969	0.000791718
566	Endophilin-B1	2.485789091	0.368837831	0.000251088
567	Double-strand break repair protein MRE11A	2.476716308	0.328982766	0.000210091
568	Thioredoxin-like protein 1	2.477719528	0.339241781	0.00022995
569	Pyridoxal kinase	2.536465528	0.566881855	0.001004242
570	PDZ domain-containing protein GIPC1	2.583880569	0.705768485	0.00188882
571	Protein Tjp1	2.439435473	0.102938008	6.90067E-06
572	Thioredoxin	2.591755462	0.749561201	0.002100531
573	Protein Rangap1	2.441401882	0.153014191	2.27683E-05
574	Protein Chd3 (Fragment)	2.43615112	0.16009812	2.68025E-05
575	Fusion, derived from t(1216) malignant liposarcoma (Human)	2.427401107	0.071519074	2.44046E-06
576	Cullin 4B (Predicted)	2.589416469	0.753876107	0.002235482
577	Cul1 protein	2.665110733	0.928749952	0.003834868
578	Protein Tnrc6b	2.430893895	0.166335754	3.12336E-05
579	Low molecular weight phosphotyrosine protein phosphatase	2.431464589	0.177205775	3.73182E-05
580	DNA-directed RNA polymerases I, II, and III subunit RPABC2	2.433303956	0.209346379	5.85164E-05
581	Protein LOC100910272	2.434736166	0.230397323	7.9867E-05
582	DNA damage-binding protein 1	2.426988623	0.196400137	5.14019E-05
583	Diphosphoinositol polyphosphate phosphohydrolase 1	2.508000856	0.591583451	0.001423111
584	Triosephosphate isomerase	2.398883798	0.018040033	4.28355E-08
585	Importin subunit alpha	2.517502636	0.645330809	0.001692994
586	Glucose-6-phosphate 1-dehydrogenase	2.528958268	0.674245517	0.001989461
587	Eukaryotic translation initiation factor 3 subunit B	2.405449503	0.205893673	6.38423E-05
588	Rho-associated protein kinase 2	2.475149932	0.533253688	0.00100466
589	Phosphoglucomutase-1	2.420221534	0.319795914	0.000233866
590	NEDD8	2.558158497	0.7647952	0.002674598
591	Protein PRRC2A	2.400667964	0.217608407	7.52537E-05
592	Protein timeless homolog	2.39026786	0.125139217	1.48586E-05
593	Acylamino-acid-releasing enzyme	2.41497878	0.321979171	0.000245036
594	LSM8 homolog, U6 small nuclear RNA associated (S. cerevisiae)	2.399499696	0.273597984	0.000155831
595	GMP synthase [glutamine-hydrolyzing]	2.377848649	0.079954972	4.02193E-06

596	Protein Bag4 (Fragment)	2.422173341	0.387469107	0.000428182
597	Leucine-rich repeat flightless-interacting protein 2	2.411967494	0.346007319	0.000315715
598	Septin-7	2.401274272	0.293331818	0.000191483
599	Cysteine and histidine-rich domain-containing protein 1	2.390711825	0.229366515	0.000100878
600	Histone H1.4	2.540338139	0.769893301	0.002658594
601	Protein Fnbp4	2.406557116	0.345703657	0.000322631
602	Biliverdin reductase A	2.452673199	0.528210006	0.001035605
603	Tyrosine-protein phosphatase non-receptor type	2.52277643	0.727961934	0.002454359
604	Cleavage and polyadenylation specific factor 6, 68kDa (Predicted), isoform CRA_b	2.409136803	0.369320213	0.000384693
605	Protein Sf3a1	2.366848048	0.076538942	3.6636E-06
606	Protein Sf3a3	2.368549976	0.128640079	1.76821E-05
607	Protein Rpia	2.492148162	0.660323799	0.001992475
608	Sorting nexin-5	2.458350958	0.564762547	0.001288593
609	Bromodomain containing 3 (Predicted), isoform CRA_b	2.369412545	0.146624819	2.89976E-05
610	Serine hydroxymethyltransferase	2.393663385	0.328148226	0.000279704
611	Transcription initiation factor IIB	2.438272544	0.50460892	0.000951426
612	Protein Rbbp4	2.406965139	0.397427574	0.000484949
613	Polyadenylate-binding protein 1	2.418957548	0.449445397	0.000695737
614	Myc box-dependent-interacting protein 1	2.432097599	0.49957345	0.000946947
615	Core-binding factor, beta subunit	2.400232955	0.384434667	0.000478602
616	Protein Ank3 (Fragment)	2.371848668	0.268854837	0.000159598
617	40S ribosomal protein S30	2.438501741	0.54476657	0.001199453
618	Protein Trp53bp1	2.383635818	0.334478358	0.000315933
619	Protein Cbx1	2.373886343	0.292352376	0.00020096
620	Proteasome subunit beta type-2	2.357149178	0.217935312	8.80503E-05
621	DnaJ (Hsp40) homolog, subfamily B, member 1 (Predicted), isoform CRA_a	2.380896629	0.360570991	0.000387679
622	Nucleosome assembly protein 1-like 1	2.348671063	0.166420285	4.14162E-05
623	Protein Birc6 (Fragment)	2.349725293	0.185561815	5.64083E-05
624	Coatomer subunit delta	2.518635339	0.779384265	0.003245143
625	Glutathione synthetase	2.350620093	0.200026125	7.06786E-05

626	UPF0160 protein MYG1, mitochondrial	2.340244334	0.093668298	7.40288E-06
627	Keratin, type I cytoskeletal 18	2.375086045	0.35743067	0.000378867
628	Protein Atxn2 (Fragment)	2.354254419	0.252201748	0.000136353
629	DEAD (Asp-Glu-Ala-Asp) box polypeptide 23 (Predicted), isoform CRA_b	2.337914146	0.166700475	4.24616E-05
630	Protein Dido1	2.415932464	0.534159717	0.00123426
631	Prolyl endopeptidase	2.350807898	0.274910947	0.000183562
632	T-complex protein 1 subunit delta	2.362279544	0.336889083	0.000339161
633	Cofilin-1	2.355224649	0.320585109	0.000303631
634	Pre-mRNA-processing factor 19	2.323008528	0.041122281	6.46278E-07
635	RRP15-like protein	2.355883775	0.327988213	0.000313679
636	Protein Hnrnpa0	2.694818188	1.161035945	0.008740701
637	Zinc finger CCCH domain-containing protein 14	2.32733728	0.170550001	4.35804E-05
638	Protein LOC100909833	2.374256122	0.435072506	0.0007338
639	Protein Pum1	2.321907105	0.166107676	4.72296E-05
640	AN1-type zinc finger protein 6	2.315694328	0.149663334	3.31488E-05
641	Protein Tsn	2.370315001	0.444488792	0.000800257
642	Non-structural maintenance of chromosomes element 1 homolog	2.403423293	0.559579625	0.001512683
643	DNA-damage inducible protein 2	2.342053513	0.344898775	0.000424281
644	Bone marrow stromal cell-derived ubiquitin-like protein	2.345334291	0.374709732	0.000509932
645	Protein Zmat2	2.302688045	0.087583299	6.81769E-06
646	Protein LOC684828	2.347874721	0.397745242	0.000603754
647	Protein Ckap5	2.305079814	0.149585727	3.43862E-05
648	Proteasome subunit beta type	2.30681023	0.182460123	6.0333E-05
649	Protein LOC100911093	2.300650795	0.166206005	4.78778E-05
650	Protein Sec16a	2.315283053	0.290810222	0.00026407
651	CD2 antigen (Cytoplasmic tail) binding protein 2 (Predicted), isoform CRA_a	2.34853127	0.441276775	0.000856625
652	Translation initiation factor eIF-2B subunit delta	2.620884676	1.07295783	0.008823497
653	Protein Umps	2.297089329	0.195086996	7.93339E-05
654	Nuclear transport factor 2	2.280946597	0.060635053	2.46988E-06
655	26S proteasome non-ATPase regulatory subunit 9	2.31356443	0.327061511	0.000370283
656	Protein Zcchc8	2.303327668	0.277017616	0.000227673

657	Methionine adenosyltransferase 2 subunit beta	2.335622342	0.422832024	0.000776072
658	Protein Rbm27 (Fragment)	2.285620745	0.178724207	6.55814E-05
659	14-3-3 protein theta	2.338832827	0.447797792	0.000933314
660	N-acetylneuraminic acid synthase	2.339012676	0.447152527	0.000918577
661	Brain-specific angiogenesis inhibitor 1-associated protein 2	2.277139117	0.11116653	1.57519E-05
662	Protein LOC100910833	2.292261375	0.270572346	0.000215002
663	Integrin-linked kinase-associated serine/threonine phosphatase 2C	2.2835215	0.226450962	0.000130823
664	Interleukin enhancer-binding factor 3	2.274150725	0.161172201	4.82718E-05
665	Heterogeneous nuclear ribonucleoprotein F	2.360615642	0.55284847	0.00169866
666	Protein LOC100911073	2.267021817	0.120190934	1.99805E-05
667	Ubiquitin carboxyl-terminal hydrolase	2.332039389	0.471648229	0.00112817
668	Uroporphyrinogen decarboxylase	2.290478401	0.302510325	0.000352936
669	Protein Xpo5	2.259747166	0.04332491	9.73011E-07
670	Protein Cd2ap (Fragment)	2.260609828	0.08411793	7.12384E-06
671	Rho guanine nucleotide exchange factor 2	2.293431432	0.334286596	0.000423796
672	Cell growth-regulating nucleolar protein	2.446920259	0.790876227	0.004587409
673	Nucleolar and coiled-body phosphoprotein 1	2.306121176	0.397418871	0.000730982
674	Alcohol dehydrogenase [NADP(+)]	2.254879872	0.062191307	2.88381E-06
675	Protein LOC100363408	2.328849489	0.486720502	0.001246398
676	Cullin-3	2.266459529	0.205892833	0.000104699
677	Zinc finger RNA-binding protein	2.266828058	0.212335945	0.000110748
678	Serine/threonine-protein kinase PAK 2	2.343030515	0.542031989	0.001669989
679	GTP-binding nuclear protein Ran	2.249865479	0.069755445	4.22188E-06
680	Replication protein A 32 kDa subunit	2.355925062	0.583859878	0.002083307
681	UPF0587 protein C1orf123 homolog	2.271152309	0.26584641	0.000227475
682	ESF1 homolog	2.303648304	0.420334844	0.000832318
683	Protein Ugp2	2.27194208	0.274367442	0.000255808
684	Peptidylprolyl isomerase domain and WD repeat containing 1 (Predicted)	2.2512015	0.113517257	1.82045E-05
685	Histidine triad nucleotide-binding protein 1	2.293116787	0.376594486	0.000620217
686	Alcohol dehydrogenase class-3	2.263642153	0.23824712	0.000164342
687	Protein Aar2	2.263642153	0.23824712	0.000164342

688	Lactoylglutathione lyase	2.263816926	0.2400583	0.000170892
689	Amyloid beta (A4) protein-binding, family B, member 1 interacting protein	2.24660719	0.127528496	2.68521E-05
690	Proteasome (Prosome, macropain) 26S subunit, non-ATPase, 5 (Predicted), isoform CRA_a	2.239697094	0.07655394	5.66224E-06
691	Origin recognition complex subunit 2	2.252093041	0.221955098	0.000138595
692	Protein Chd4	2.252981157	0.233476667	0.000164969
693	LOC64038 protein	2.295143191	0.430809009	0.000946352
694	Heterogeneous nuclear ribonucleoprotein Q	2.256701986	0.27859262	0.000270332
695	Non-SMC condensin II complex, subunit D3	2.246631426	0.217541631	0.000133473
696	NMDA receptor-regulated gene 1 (Predicted), isoform CRA_b	2.341837742	0.590905256	0.002317736
697	Protein max	2.257601731	0.287066051	0.00030705
698	Protein Rsf1	2.544855544	1.033861607	0.009063621
699	Protein Wasf2	2.238319256	0.168031642	6.39404E-05
700	Arf-GAP domain and FG repeat-containing protein 1	2.228576366	0.028893873	3.33246E-07
701	OTU domain containing 6B (Predicted), isoform CRA_b	2.250817136	0.269221942	0.000253386
702	Fructose-bisphosphate aldolase C	2.251108736	0.272853723	0.000263802
703	Pre-mRNA 3'-end-processing factor FIP1	2.230759315	0.117984389	2.14269E-05
704	Mitogen activated protein kinase 14, isoform CRA_a	2.541027978	1.067478601	0.00974245
705	ADP-ribosylation factor-like protein 6-interacting protein 4	2.243010998	0.237220058	0.000179125
706	PHD finger protein 3 (Predicted)	2.219443174	0.085896282	8.85845E-06
707	Protein Strn4	2.315896145	0.561853361	0.002060224
708	DNA-directed RNA polymerases I, II, and III subunit RPABC1	2.3828963	0.735637745	0.004298606
709	COP9 (Constitutive photomorphogenic) homolog, subunit 7b (Arabidopsis thaliana) (Predicted), isoform CRA_b	2.254781467	0.357062578	0.000616919
710	DEAD (Asp-Glu-Ala-Asp) box polypeptide 56	2.439113592	0.859808609	0.006361457
711	ADP-sugar pyrophosphatase	2.216497147	0.141972011	4.03514E-05
712	Tyrosine-protein phosphatase non-receptor type 11	2.279389006	0.462681852	0.001275929
713	Vacuolar-sorting protein SNF8	2.371937111	0.74063331	0.004482802
714	Protein Ube2a	2.203972919	0.078498004	7.10378E-06
715	Protein Wtap	2.204394469	0.093019941	1.1783E-05
716	Protein Ubxn6	2.256555099	0.41165029	0.000947135

717	Proteasome subunit alpha type-2	2.26273985	0.453275886	0.001267534
718	40S ribosomal protein S29	2.27324371	0.488258661	0.001565456
719	T-complex protein 1 subunit epsilon	2.263826687	0.462073936	0.001343071
720	Serine/arginine-rich splicing factor 9	2.20350196	0.181361093	9.4104E-05
721	Protein Brpf1	2.275883791	0.505378797	0.001715351
722	Heterogeneous nuclear ribonucleoprotein C	2.286578378	0.54044286	0.002076008
723	Hydroxymethylbilane synthase	2.204321196	0.194982248	0.000112253
724	DNA mismatch repair protein Msh2	2.194221045	0.09125034	1.14722E-05
725	Protein Txndc17	2.189107365	0.08863393	1.09449E-05
726	6-phosphogluconate dehydrogenase, decarboxylating	2.204814808	0.265467691	0.000295423
727	Calcium homeostasis endoplasmic reticulum protein (Predicted)	2.260033136	0.499900873	0.001772468
728	Dihydrofolate reductase	2.180412575	0.125387735	3.22455E-05
729	Huntingtin interacting protein 1, isoform CRA_a	2.192538156	0.239726043	0.000218804
730	DEAD (Asp-Glu-Ala-Asp) box polypeptide 47, isoform CRA_a	2.243814134	0.460867237	0.001447085
731	Transcriptional regulator ATRX (Fragment)	2.21353806	0.348919313	0.000663553
732	Calpain-1 catalytic subunit	2.245323994	0.472040999	0.001548893
733	Alpha-actinin-4	2.187532888	0.239229259	0.000224539
734	Protein Smek1	2.229252631	0.433158441	0.001241649
735	DNA fragmentation factor subunit beta	2.200529872	0.323067002	0.000554855
736	ADP-ribosylhydrolase like 2	2.297201161	0.647701227	0.003741432
737	Septin-9	2.173959979	0.190361469	0.000117524
738	40S ribosomal protein S28	2.1850684	0.267941323	0.000325143
739	Protein LOC100910660	2.240570135	0.504158496	0.001965886
740	Protein Zfp638	2.169911564	0.204480326	0.000146045
741	Paraspeckle component 1	2.161322763	0.142976855	5.3756E-05
742	E3 ubiquitin-protein ligase NEDD41	2.191806774	0.333471545	0.000629664
743	PRP3 pre-mRNA processing factor 3 homolog (Yeast) (Predicted), isoform CRA_a	2.162253538	0.161161502	7.41396E-05
744	DNA polymerase alpha subunit B	2.163810912	0.186998487	0.000115885
745	Thioredoxin-interacting protein	2.256009963	0.561993062	0.002674639
746	Adenylate kinase 2, mitochondrial	2.266855878	0.595762904	0.003136468
747	Protein U2surp	2.153346831	0.178091639	0.000104124

748	Protein U2surp	2.153346831	0.178091639	0.000104124
749	Protein Pnn	2.173334243	0.299865149	0.000482561
750	Deoxyhypusine hydroxylase	2.174049771	0.306485656	0.000521484
751	Echinoderm microtubule-associated protein-like 2	2.184411589	0.35553105	0.000776625
752	LOC682908 protein	2.319558053	0.746011656	0.005684777
753	Protein LOC100360750	2.154621456	0.19741236	0.000141889
754	Nucleolar protein 12	2.265794552	0.609464123	0.003652003
755	Protein Rfc4	2.146586683	0.145283108	5.80841E-05
756	Uncharacterized protein	2.14773786	0.167008959	8.59864E-05
757	Proteasome subunit beta type-6	2.168101691	0.296976903	0.000478425
758	CTP synthase	2.140161674	0.112045156	2.69479E-05
759	Exportin 4 (Predicted)	2.190565661	0.403123683	0.001123615
760	Nuclear cap-binding protein subunit 2	2.182801581	0.380841761	0.00098564
761	Protein Fam50a	2.163612026	0.301299231	0.000506088
762	Isoform 2 of Serine/threonine-protein phosphatase 2B catalytic subunit alpha isoform	2.143972925	0.185284091	0.000122028
763	60S acidic ribosomal protein P2	2.154862077	0.261793204	0.000340872
764	Protein Msh6	2.195065366	0.435734478	0.001480468
765	DnaJ (Hsp40) homolog, subfamily C, member 7, isoform CRA_a	2.145219351	0.203055169	0.000166081
766	Exosome complex component RRP45	2.158497354	0.299568117	0.000517799
767	Protein Npepps	2.139986005	0.198837366	0.000154078
768	Menin	2.141730886	0.223334598	0.000214156
769	Protein Ube2o (Fragment)	2.202703702	0.483171463	0.002099318
770	Ribosome biogenesis protein BOP1	2.206809441	0.507397744	0.002322598
771	Proteasome subunit alpha type	2.119282214	0.072156268	7.79113E-06
772	Cellular nucleic acid-binding protein	2.27182468	0.686415768	0.005153393
773	Asparagine synthetase [glutamine-hydrolyzing]	2.211815898	0.536765094	0.002763181
774	Dihydropyrimidinase-related protein 2	2.152206971	0.333098189	0.000711824
775	Tumor protein D54	2.122968641	0.161519679	8.75037E-05
776	Coatomer subunit gamma-1	2.145573787	0.314999368	0.000651884
777	Protein Aif1l	2.119820091	0.188871641	0.000144947
778	Mothers against decapentaplegic homolog 4	2.271844667	0.707003229	0.005725885

779	Eukaryotic translation initiation factor 3 subunit H	2.130532006	0.263037871	0.000378925
780	Inositol monophosphatase 1	2.122998982	0.232806705	0.000262349
781	Queuine tRNA-ribosyltransferase	2.113818854	0.171057365	0.000108205
782	Translation initiation factor eIF-2B subunit epsilon	2.224635605	0.60717768	0.004033179
783	Protein Yy1	2.117584066	0.225085304	0.000246846
784	Protein Epb4.1	2.109855358	0.184608631	0.000141535
785	tRNA-splicing ligase RtcB homolog	2.100832211	0.102985943	2.45517E-05
786	Uncharacterized protein (Fragment)	2.113300843	0.231293755	0.000274691
787	Protein Clip1	2.133315754	0.335330788	0.00079919
788	DOT1-like, histone H3 methyltransferase (S. cerevisiae) (Predicted), isoform CRA_a	2.09479505	0.061719347	5.4097E-06
789	Lsm14a protein	2.196970858	0.564116297	0.003378991
790	Proteasome subunit alpha type	2.092531987	0.134380954	5.68126E-05
791	40S ribosomal protein S19	2.133088528	0.373836116	0.001136728
792	COP9 signalosome complex subunit 3	2.084994454	0.05298498	3.50669E-06
793	Isoform 2 of Coiled-coil and C2 domain-containing protein 1A	2.095724207	0.18939546	0.000159229
794	T-complex protein 1 subunit gamma	2.086767474	0.112811947	3.36454E-05
795	Protein Tab1	2.096392112	0.199219017	0.000185352
796	Anapc2 protein	2.106960543	0.268470767	0.0004547
797	SH3 domain-containing kinase-binding protein 1	2.291072275	0.814863027	0.009467534
798	COP9 (Constitutive photomorphogenic) homolog, subunit 7a (Arabidopsis thaliana) (Predicted)	2.081130291	0.089182917	1.72797E-05
799	Cleavage and polyadenylation specificity factor subunit 7	2.130661516	0.391153689	0.001356877
800	Hexamthylene bis-acetamide inducible 2 (Predicted)	2.111902866	0.316470468	0.000745966
801	Protein phosphatase 1A	2.076958604	0.106639918	2.96418E-05
802	TIP41-like protein	2.128277476	0.408575314	0.001547078
803	Bcl2-associated athanogene 3	2.100476782	0.298561928	0.000642902
804	GRIP1-associated protein 1	2.210308603	0.651936555	0.005864002
805	Inositol monophosphatase 2	2.093252718	0.276471847	0.000498167
806	Protein Uchl5	2.27399808	0.793973429	0.008923229
807	Uncharacterized protein	2.112829646	0.363385564	0.001131115
808	Uncharacterized protein	2.112829646	0.363385564	0.001131115

809	Protein Vill	2.094126421	0.284129287	0.000561203
810	DnaJ (Hsp40) homolog, subfamily C, member 6 (Predicted)	2.202722083	0.643218329	0.005417636
811	Protein Ubfd1	2.144210374	0.478523895	0.002446877
812	Protein Chd1l	2.089374259	0.283269196	0.000569807
813	Protein Crlf3 (Fragment)	2.092013548	0.309074019	0.000728944
814	Copper transport protein ATOX1	2.064601358	0.148430643	8.36618E-05
815	Y-box-binding protein 3	2.095427227	0.337764655	0.000963363
816	Protein RGD1311021	2.106559016	0.389593808	0.001452342
817	Protein Slfn5 (Fragment)	2.126889543	0.461190105	0.002322382
818	Protein Cmc1	2.070104835	0.228904313	0.00031769
819	Catenin (Cadherin associated protein), delta 1 (Predicted), isoform CRA_a	2.052032246	0.06899939	9.03718E-06
820	Protein Kcmf1 (Fragment)	2.121780326	0.457378623	0.002326518
821	Protein Ahnak	2.056604201	0.172844779	0.00014227
822	Protein Eny2	2.113380911	0.431422321	0.002207938
823	Protein Iqsec1	2.125965206	0.486503741	0.002738324
824	Neurabin-2	2.049326581	0.125479046	5.54085E-05
825	Transcription initiation factor TFIID subunit 6	2.097806012	0.393344297	0.001521386
826	Isoform 2 of Calcineurin subunit B type 1	2.050073065	0.14119419	7.75723E-05
827	Eukaryotic translation initiation factor 4E	2.051333527	0.163850698	0.000121688
828	Protein Mxra7	2.051988737	0.174608962	0.000146933
829	Phosphoglucomutase 1	2.043665338	0.102503689	3.08742E-05
830	Epidermal growth factor receptor pathway substrate 15-like 1	2.091637956	0.381420103	0.001452487
831	Protein Sh3bp1	2.06393339	0.265489947	0.000516763
832	Protein Cnot3	2.083930973	0.356165428	0.001328458
833	Glutathione S-transferase Mu 2	2.123923471	0.497817891	0.003266415
834	Hsp70-binding protein 1	2.038920541	0.101301844	2.98232E-05
835	Protein Tmod3	2.165300173	0.614876393	0.005335408
836	Protein Hnrnpl	2.078298503	0.351855096	0.001199939
837	Protein Frg1	2.097907272	0.422667315	0.00210076
838	Protein Vbp1	2.032701871	0.043231345	2.41177E-06
839	Protein Shcbp1	2.032744992	0.045859315	2.87891E-06

840	Isoform 5 of Gephyrin	2.072422636	0.342591759	0.001095922
841	Protein Ttc3	2.053560021	0.254802841	0.000475704
842	Aminopeptidase B	2.036113222	0.142956808	8.62276E-05
843	NADP-dependent malic enzyme	2.036102988	0.142367054	8.73013E-05
844	Protein Scrib	2.037359861	0.164870004	0.00013397
845	Septin-2	2.116177277	0.51088295	0.003428602
846	Bifunctional lysine-specific demethylase and histidyl-hydroxylase MINA	2.12759439	0.546334253	0.004371477
847	DNA-(apurinic or apyrimidinic site) lyase	2.05235445	0.289141868	0.000722482
848	RNA polymerase II-associated protein 1	2.0819827	0.419371017	0.001838865
849	Glutamate-rich WD repeat-containing protein 1	2.053833127	0.302538969	0.000819421
850	Protein Dera (Fragment)	2.027161783	0.149686381	0.000104433
851	Calpastatin	2.064819602	0.355834602	0.001353461
852	PDZ and LIM domain protein 1	2.020355666	0.103733809	3.48561E-05
853	ADP-ribosylation factor-like protein 3 (Fragment)	2.155335746	0.632670409	0.006320739
854	Protein Rbm15	2.021202657	0.123409277	6.08777E-05
855	Matrin-3	2.150806906	0.639324565	0.006735019
856	Protein Hectd1	2.017142031	0.135573626	8.08357E-05
857	Protein LOC100909510	2.027448353	0.222328757	0.000342749
858	UPF0586 protein C9orf41 homolog	2.0187232	0.163974049	0.000143091
859	Pls1 protein	2.020091222	0.185086247	0.000205868
860	Eukaryotic translation initiation factor 3 subunit D	2.01243254	0.133796555	7.94479E-05
861	Cysteine sulfinic acid decarboxylase	2.050418167	0.351140808	0.001411923
862	Catenin, beta-interacting protein 1	2.032552186	0.276869386	0.000687119
863	Rab GTPase-binding effector protein 2	2.033535859	0.286947713	0.000745076
864	Protein Tnpo1 (Fragment)	2.016844898	0.204689836	0.000279333
865	LysM and putative peptidoglycan-binding domain-containing protein 3	0.499355449	0.025046115	0.000273175
866	Atlastin-1	0.502136639	0.052975193	0.002501357
867	Protein Edil3 (Fragment)	0.504878151	0.080679227	0.008147844
868	Protein Tmem201	0.505081522	0.082221711	0.008486099
869	Transmembrane 9 superfamily member 1	0.50569999	0.087388399	0.009855
870	Protein Atl2	0.501745928	0.072880935	0.006422313
871	Coiled-coil domain-containing protein 51	0.492322006	0.019301644	0.000123193

872	Isocitrate dehydrogenase [NAD] subunit alpha, mitochondrial	0.49482534	0.046824959	0.001689014
873	Protein Cnep1r1	0.491193487	0.019379286	0.000125236
874	Plexin B2	0.494389402	0.055604102	0.002827164
875	Antigen peptide transporter 1	0.489929455	0.014027951	4.85372E-05
876	Unconventional myosin-IXb	0.488033529	0.025551502	0.000299266
877	Alpha-soluble NSF attachment protein	0.488531916	0.036153292	0.000810089
878	40S ribosomal protein S23	0.495601233	0.078554668	0.007504178
879	Protein Poglut1	0.493344962	0.068618158	0.005128982
880	Protein Trappc5	0.48661696	0.016460941	7.99867E-05
881	60S ribosomal protein L36a	0.487073082	0.029485408	0.000435181
882	Protein Ssfa2	0.490520721	0.062493861	0.00393149
883	Vesicle transport through interaction with t-SNAREs homolog 1B	0.484678385	0.025521786	0.000295616
884	Protein Nat10	0.48474116	0.027114634	0.000346019
885	ADP-dependent glucokinase, isoform CRA_a	0.49163569	0.07446286	0.005764666
886	Enoyl-CoA hydratase domain-containing protein 3, mitochondrial	0.49154808	0.072572321	0.005995639
887	Protein Mrpl12	0.489567169	0.064185774	0.004339288
888	Protein Ndufb10	0.482023072	0.009819431	1.69794E-05
889	Mitochondrial ribosomal protein S18A	0.484739929	0.046579844	0.001700791
890	Protein Nus1	0.489276812	0.072264865	0.005929407
891	Glioblastoma amplified sequence	0.490986443	0.08615103	0.009579263
892	Signal recognition particle subunit SRP72	0.48053114	0.031965697	0.000564357
893	Protein Mrps17	0.477745336	0.016557359	8.1546E-05
894	B-cell receptor-associated protein 29	0.483716916	0.071607356	0.005885659
895	Uncharacterized protein	0.481532127	0.07208099	0.005650936
896	A disintegrin and metalloproteinase domain 17 (Tumor necrosis factor, alpha, converting enzyme), isoform CRA_a	0.479289583	0.060443656	0.003603091
897	DnaJ homolog subfamily C member 25	0.473390233	0.040910838	0.00117194
898	ATP-dependent Clp protease proteolytic subunit	0.472042122	0.036669259	0.000839055
899	F-box and leucine-rich repeat protein 4 (Predicted), isoform CRA_c	0.472398915	0.042257365	0.001299654
900	3-hydroxyisobutyrate dehydrogenase, mitochondrial	0.472793793	0.047937042	0.001832667
901	Vezatin	0.470901338	0.035581254	0.000779551
902	39S ribosomal protein L22, mitochondrial	0.47111094	0.039121645	0.001029744

903	Phosphatidylinositol 3-kinase, C2 domain containing, alpha polypeptide (Predicted), isoform CRA_a	0.479977	0.08519021	0.009618724
904	F-box protein 28	0.470370053	0.044185763	0.001461772
905	Protein Glce	0.46825511	0.02543177	0.00029388
906	Protein Lamb1	0.466871408	0.016281383	7.52636E-05
907	Syntaxin-12	0.468156504	0.043163303	0.001392149
908	Peptidyl-prolyl cis-trans isomerase	0.464525994	0.036298098	0.000883714
909	Protein LOC100910864	0.464946718	0.04310183	0.001373131
910	Coiled-coil-helix-coiled-coil-helix domain containing 3 (Predicted), isoform CRA_a	0.464123604	0.046765654	0.001647739
911	Phosphatidylinositol 5-phosphate 4-kinase type-2 gamma	0.468552537	0.070852209	0.005686235
912	NAD kinase 2, mitochondrial	0.460289508	0.006350853	4.6368E-06
913	Protein Lmf1	0.460699655	0.023331404	0.000224884
914	Protein Clptm1l	0.461488813	0.038878264	0.001034398
915	Disks large-associated protein 3	0.464081529	0.058295594	0.003375549
916	Procollagen-lysine, 2-oxoglutarate 5-dioxygenase 1	0.458222925	0.037219914	0.000899353
917	Interferon-induced transmembrane protein 3	0.46175265	0.066760569	0.004764994
918	Phospholipase A2, group VII (Platelet-activating factor acetylhydrolase, plasma)	0.455823019	0.031845317	0.000569579
919	Ribosomal protein S5, isoform CRA_b	0.45836021	0.0532355	0.002596687
920	Protein Reep3	0.458972885	0.059829517	0.003565058
921	Chromatin modifying protein 2A	0.459723505	0.067149205	0.004882562
922	Protein Tomm6	0.458358813	0.064288743	0.004354902
923	Type 2 phosphatidylinositol 4,5-bisphosphate 4-phosphatase	0.453362239	0.042611039	0.001370019
924	Protein Sucla2	0.462178636	0.086753092	0.009879749
925	Golgi resident protein GCP60	0.460397975	0.0817159	0.008447638
926	Fibronectin type III domain containing 3a (Predicted), isoform CRA_a	0.44973577	0.034477989	0.000724873
927	Prolactin regulatory element-binding protein	0.452281503	0.054871074	0.002847284
928	Dehydrogenase/reductase SDR family member 7B (Fragment)	0.449595455	0.047432142	0.001873172
929	Phosphatidylglycerophosphatase and protein-tyrosine phosphatase 1	0.45021446	0.054696365	0.002796185
930	Fos-related antigen 2	0.44716488	0.042426291	0.001325417
931	Hydroxymethylglutaryl-CoA lyase, mitochondrial	0.449287418	0.056264421	0.002833133

932	Copine 3 protein	0.449600716	0.068695499	0.005260412
933	NADH dehydrogenase [ubiquinone] 1 subunit C2	0.441573923	0.00203819	1.5352E-07
934	Protein Nhlrc3	0.450225633	0.074106788	0.006396505
935	Protein Rabl3	0.442708077	0.036642216	0.000871441
936	Protein Gramd4 (Fragment)	0.451501057	0.083759215	0.009043365
937	Protein LOC100362069	0.443407758	0.046550841	0.001793715
938	Transcription factor A, mitochondrial	0.44553723	0.068956032	0.005273698
939	Protein Rragc	0.448183833	0.081679359	0.008539823
940	RCG24191	0.442163677	0.055546129	0.002984676
941	Kin of IRRE-like protein 1	0.446837579	0.078983976	0.00780983
942	Protein Cln5 (Fragment)	0.437266096	0.029590747	0.000469505
943	Mitochondrial tRNA-specific 2-thiouridylase 1	0.44797278	0.088040044	0.00984626
944	Neuropathy target esterase like 1	0.438611332	0.049564213	0.002122265
945	Protein Lmbrd2	0.437806776	0.051896685	0.002357015
946	Protein Fndc3b	0.44436945	0.08305784	0.008880193
947	Protein Tbl2	0.432550522	0.006505654	5.12765E-06
948	Delta(3,5)-Delta(2,4)-dienoyl-CoA isomerase, mitochondrial	0.432116575	0.026196016	0.000334813
949	Protein Sec61b	0.436254954	0.056642425	0.003073146
950	Hydroxymethylglutaryl-CoA synthase, cytoplasmic	0.436281219	0.056714431	0.00319295
951	Mitochondrial ribosomal protein L45 (Predicted)	0.436370247	0.057659818	0.00326284
952	Ras-related protein Rab-7a	0.431536064	0.034079166	0.000718843
953	Unconventional myosin-Ie	0.433754257	0.050790605	0.002338662
954	PQ loop repeat containing 3	0.43623596	0.065763119	0.004767317
955	Protein Abcc4	0.430343039	0.0304659	0.000510764
956	Transforming growth factor beta-1	0.433988897	0.063270399	0.004290854
957	UPF0562 protein C7orf55 homolog	0.430477739	0.047083514	0.001852603
958	Cleft lip and palate associated transmembrane protein 1	0.429127455	0.042292667	0.001354768
959	NEDD4 family-interacting protein 1	0.432281141	0.065501305	0.004742417
960	G protein-coupled receptor 107 (Predicted)	0.428992821	0.052473397	0.002538784
961	Peroxisomal targeting signal 1 receptor	0.425925953	0.038491939	0.001037911
962	40S ribosomal protein S6 (Fragment)	0.430031044	0.063038438	0.004274048
963	Putative phospholipase B-like 2	0.430302503	0.065425112	0.004742731

964	FK506 binding protein 10	0.426359046	0.044447989	0.001578407
965	Pdk3 protein	0.428739284	0.060157825	0.003681124
966	Mitochondrial inner membrane protein (Fragment)	0.432715928	0.076864504	0.007416254
967	Ornithine aminotransferase, mitochondrial	0.426929092	0.051346094	0.002393825
968	Trifunctional enzyme subunit alpha, mitochondrial	0.423031714	0.020233949	0.000155502
969	Acetyl-coenzyme A transporter 1	0.430237163	0.072838941	0.006485789
970	Ras-related protein Rab-4B	0.424400657	0.044151509	0.001584506
971	Golgi integral membrane protein 4	0.423585102	0.046343902	0.001755368
972	Protein Slc35b4	0.429755473	0.076565892	0.007342891
973	Protein GPR108	0.426015115	0.061707335	0.004051031
974	Protein Vwa8 (Fragment)	0.423129279	0.052002788	0.002499473
975	Unconventional myosin-Va	0.428410019	0.080769262	0.008778547
976	Ab2-292	0.420986504	0.049669083	0.002198885
977	Transcriptional activator protein Pur-alpha	0.420971198	0.059307689	0.003713605
978	Natural resistance-associated macrophage protein 2	0.41974792	0.056783937	0.003235736
979	Type-1 angiotensin II receptor-associated protein	0.418345127	0.061533637	0.00407544
980	NADH dehydrogenase (Ubiquinone) Fe-S protein 3 (Predicted), isoform CRA_c	0.414529685	0.040513429	0.001232392
981	Dehydrogenase/reductase (SDR family) member 1	0.411311102	0.013300956	4.54956E-05
982	Leucine-rich PPR motif-containing protein, mitochondrial	0.413777946	0.042989321	0.00148606
983	Protein Emc6	0.411939308	0.029465456	0.00048567
984	Protein B4galt5	0.415803269	0.055315725	0.003025852
985	Protein Tapbpl	0.412410806	0.037251328	0.000968145
986	28S ribosomal protein S15, mitochondrial	0.410582386	0.020399357	0.000160256
987	Nuclear pore complex protein Nup85	0.42021362	0.077100678	0.007385408
988	Mitochondrial ribosomal protein S18C (Predicted), isoform CRA_a	0.418371089	0.069748077	0.005878328
989	Kinase D-interacting substrate of 220 kDa	0.412792506	0.042935907	0.001355499
990	Protein Slc30a5	0.409512415	0.01658058	8.82683E-05
991	Golgin subfamily A member 7	0.421423696	0.085208462	0.009956237
992	Core histone macro-H2A.1	0.407739248	0.019772102	0.000148519
993	Heme oxygenase 1	0.412215309	0.056892576	0.003302191
994	H/ACA ribonucleoprotein complex subunit 1	0.40676774	0.01888436	0.000123442

995	DEAD/H (Asp-Glu-Ala-Asp/His) box polypeptide 3, X-linked	0.414440068	0.068434949	0.005642411
996	Phospholipid scramblase 3	0.407402816	0.032240888	0.00064597
997	Mitochondrial ribonuclease P protein 1	0.411650735	0.060331126	0.00392504
998	Achalasia, adrenocortical insufficiency, alacrimia (Allgrove, triple-A) (Predicted), isoform CRA_a	0.410900612	0.062505978	0.004162232
999	Protein LOC100360065	0.404112046	0.022422524	0.000219801
1000	cGMP-dependent 3',5'-cyclic phosphodiesterase	0.411839869	0.070715459	0.005592961
1001	NADH dehydrogenase [ubiquinone] 1 alpha subcomplex subunit 6	0.408655443	0.058663209	0.003631104
1002	39S ribosomal protein L41, mitochondrial	0.409454072	0.065499344	0.00494599
1003	Protein Tmem173	0.404111784	0.038708445	0.001114474
1004	Electron transfer flavoprotein subunit alpha, mitochondrial	0.400254384	0.018179146	0.000118745
1005	Epidermal growth factor receptor kinase substrate 8	0.411740758	0.082390837	0.009277706
1006	Protein Wdtc1	0.406910649	0.066938001	0.005618359
1007	Mitochondrial-processing peptidase subunit beta	0.41016847	0.084620126	0.009987562
1008	ATP-dependent Clp protease ATP-binding subunit clpX-like, mitochondrial	0.400008416	0.044989027	0.001727616
1009	Hexaprenyldihydroxybenzoate methyltransferase, mitochondrial	0.396492068	0.015023562	6.86143E-05
1010	Coiled-coil domain containing 115	0.401994377	0.056135851	0.00343909
1011	Carnitine O-palmitoyltransferase 2, mitochondrial	0.408008258	0.082843067	0.009147322
1012	Cationic amino acid transporter-1	0.406193762	0.075974954	0.007650705
1013	60S ribosomal protein L3	0.399965408	0.054427324	0.002958219
1014	60S ribosomal protein L11	0.395972436	0.039960931	0.001235711
1015	Coiled-coil domain-containing protein 127	0.402578099	0.075647	0.007626667
1016	Acetoacetyl-CoA synthetase	0.397142879	0.053617719	0.002782312
1017	Trafficking protein particle complex subunit 1	0.391877613	0.011885285	3.47594E-05
1018	SPARC	0.395885337	0.049609669	0.002305946
1019	Glutamine--fructose-6-phosphate aminotransferase [isomerizing] 1	0.400060462	0.071117161	0.006235793
1020	Calumenin	0.395642057	0.056056319	0.003394568
1021	Carnitine O-palmitoyltransferase 1, liver isoform	0.394091907	0.049668296	0.002320665
1022	Protein Ptcd3	0.396303257	0.062471519	0.00432075
1023	H/ACA ribonucleoprotein complex subunit 4	0.397773181	0.073522267	0.00699976
1024	Poly (ADP-ribose) polymerase family, member 3	0.397976636	0.075179521	0.007329513

1025	Methylcrotonoyl-CoA carboxylase beta chain, mitochondrial	0.390708946	0.041855054	0.001379937
1026	Protein Coa3	0.391163834	0.046858619	0.00206875
1027	Probable phospholipid-transporting ATPase IIB	0.388172451	0.030857811	0.000575298
1028	Golgi SNAP receptor complex member 2	0.390059572	0.044604261	0.001708758
1029	NADH dehydrogenase [ubiquinone] 1 alpha subcomplex subunit 10, mitochondrial	0.388547984	0.03655179	0.000970751
1030	Pyrroline-5-carboxylate reductase 2	0.394940661	0.072718375	0.006741137
1031	40S ribosomal protein S11	0.386208852	0.027365687	0.000423632
1032	Antigen peptide transporter 2	0.388004969	0.041232526	0.001363756
1033	Saccharopine dehydrogenase-like oxidoreductase	0.38986419	0.051978977	0.002689951
1034	ER membrane protein complex subunit 3	0.384701133	0.010610369	2.43298E-05
1035	Nucleoporin 205kDa (Predicted)	0.391077946	0.063127299	0.004658269
1036	Acad9 protein	0.385865991	0.036151423	0.000963321
1037	Extended synaptotagmin-1	0.383103371	0.016843022	0.000100635
1038	F-box/LRR-repeat protein 20	0.387044569	0.050240097	0.002456204
1039	2,4-dienoyl CoA reductase 1, mitochondrial, isoform CRA_a	0.387461469	0.054357048	0.003075106
1040	NADH-ubiquinone oxidoreductase 75 kDa subunit, mitochondrial	0.38143252	0.019303116	0.000149844
1041	Protein RGD1310352	0.390470726	0.072175606	0.00677665
1042	Hexose-6-phosphate dehydrogenase (Glucose 1-dehydrogenase) (Predicted), isoform CRA_b	0.385289394	0.050224543	0.002478069
1043	Isoform 2 of Long-chain-fatty-acid--CoA ligase 6	0.384595237	0.052346583	0.002700413
1044	RAB10, member RAS oncogene family	0.38994863	0.074646895	0.007574723
1045	Tyrosine-protein phosphatase non-receptor type 9	0.388803338	0.07317401	0.006821907
1046	Collagen alpha-1(III) chain	0.382592278	0.049146706	0.002517128
1047	Protein YIF1B	0.386463598	0.067989229	0.005869454
1048	Protein Man1a2	0.379603316	0.034288047	0.000825636
1049	Hexokinase-2	0.380230838	0.042611122	0.001556492
1050	Protein Rab5a	0.381004127	0.051075898	0.002622573
1051	Mitochondrial import receptor subunit TOM22 homolog	0.387430841	0.081091133	0.00954087
1052	Glycerophosphodiester phosphodiesterase 1	0.379040345	0.048607881	0.002287349
1053	Superoxide dismutase [Mn], mitochondrial	0.383469891	0.071606246	0.007018292
1054	Membrane-associated progesterone receptor component 1	0.375041941	0.042380692	0.001556571

1055	Transmembrane emp24 domain-containing protein 1	0.371610445	0.008669672	1.3538E-05
1056	Ras-related protein Rab-2A	0.371633784	0.009880659	2.0828E-05
1057	Glucocorticoid receptor	0.373359853	0.031070975	0.000622463
1058	Lysophosphatidylcholine acyltransferase 1	0.379623038	0.069942679	0.005535384
1059	GrpE protein homolog 1, mitochondrial	0.376101895	0.060928023	0.004465392
1060	Protein Tnfrsf12a	0.374838063	0.064601297	0.005126558
1061	ATP synthase subunit s, mitochondrial	0.374867494	0.064544792	0.005172658
1062	Trophoblast glycoprotein	0.365814857	0.014655075	6.89076E-05
1063	Nuclear pore complex protein Nup155	0.374985794	0.071591922	0.006915971
1064	Protein Rab22a	0.365206664	0.021001752	0.000202577
1065	Protein Abcd4	0.37265466	0.066860126	0.005763158
1066	Lamin-B receptor	0.364310862	0.019571619	0.000167824
1067	DNA dC->dU-editing enzyme APOBEC3	0.364852604	0.030198217	0.000593265
1068	Transcription factor AP-1	0.36397938	0.029564816	0.000561147
1069	Flotillin-2	0.364079881	0.031197443	0.000657414
1070	Lipid phosphate phosphohydrolase 3	0.361781717	0.018940635	0.000150724
1071	ADP-ribosylation factor-like 6 interacting protein 1	0.362257485	0.028631132	0.000513469
1072	Protein Tmem101	0.360902231	0.017618728	0.000123633
1073	Mitochondrial ribosomal protein L4 (Predicted), isoform CRA_b	0.360231956	0.021611507	0.000217296
1074	Phosphatidylinositol 4-kinase type 2-alpha	0.367223402	0.063938011	0.005031615
1075	Protein Wdr37	0.369185065	0.071994805	0.007267649
1076	Endoplasmic reticulum aminopeptidase 1	0.366291551	0.062801707	0.005011096
1077	Flotillin-1	0.359838754	0.029646309	0.000573331
1078	Protein Fitm2	0.360301574	0.03634961	0.001059733
1079	Mitochondrial 2-oxoglutarate/malate carrier protein	0.358943783	0.028237838	0.000540261
1080	Vesicle-trafficking protein SEC22b	0.355996645	0.01864184	0.000145344
1081	Calumenin	0.364660887	0.069297091	0.006532297
1082	KDEL (Lys-Asp-Glu-Leu) containing 1, isoform CRA_b	0.356365245	0.026372397	0.000415401
1083	Alpha-mannosidase 2	0.363304403	0.064910259	0.005795453
1084	Electron transfer flavoprotein subunit beta	0.365559817	0.07501992	0.008320595
1085	Fatty acid desaturase 2	0.364606494	0.074757903	0.00790374
1086	Protein Mga	0.35479585	0.027459672	0.000468249

1087	Protein Nbas	0.358165221	0.048944129	0.002640179
1088	Guanine nucleotide-binding protein subunit gamma (Fragment)	0.365421068	0.079727722	0.009270224
1089	Protein Pign	0.363743031	0.073471716	0.007969395
1090	Protein Pi4ka	0.353232434	0.028444024	0.00052663
1091	40S ribosomal protein S3a	0.361158848	0.072701735	0.007594716
1092	Protein LOC100360884	0.359775898	0.069220151	0.006620037
1093	Annexin	0.359357768	0.077142114	0.008921612
1094	AMP deaminase 3	0.350650746	0.046575384	0.002226775
1095	NADH dehydrogenase (Ubiquinone) flavoprotein 1	0.354267048	0.064081935	0.005446582
1096	Protein Tmem2	0.351073494	0.050575164	0.00279296
1097	Protein disulfide-isomerase A6	0.345381674	0.014804741	7.69314E-05
1098	28S ribosomal protein S26, mitochondrial	0.347126767	0.042856678	0.001774094
1099	6-phosphofructokinase, liver type	0.342171656	0.013242629	5.58628E-05
1100	Asparagine-linked glycosylation 1 homolog (Yeast, beta-1,4-mannosyltransferase) (Predicted)	0.35061699	0.065898615	0.005987139
1101	Syntaxin-8	0.345165394	0.054066892	0.003488394
1102	Syntaxin-8	0.345165394	0.054066892	0.003488394
1103	Tripartite motif-containing protein 26	0.338991483	0.011541834	3.7532E-05
1104	Transcription elongation factor, mitochondrial (Fragment)	0.346089199	0.061738218	0.005059094
1105	Proto-oncogene tyrosine-protein kinase Src	0.340892359	0.043193428	0.001861138
1106	Protein LOC685778	0.347538904	0.072047829	0.007723735
1107	Peptidyl-prolyl cis-trans isomerase B	0.34123565	0.046511632	0.002390476
1108	Transmembrane protein 165	0.338691286	0.035646007	0.001072875
1109	Asparagine-linked glycosylation 2 homolog (Yeast, alpha-1,3-mannosyltransferase), isoform CRA_a	0.338097471	0.03789443	0.001254175
1110	Protein Uap1l1	0.346440975	0.074765162	0.008249472
1111	Membrane-associated ring finger (C3HC4) 5	0.336137854	0.032510782	0.000803833
1112	Succinate dehydrogenase [ubiquinone] iron-sulfur subunit, mitochondrial	0.336225336	0.033558409	0.000915036
1113	Aspartate aminotransferase, mitochondrial	0.335471903	0.033788339	0.000929706
1114	4F2 cell-surface antigen heavy chain	0.340811654	0.062567324	0.005466431
1115	Malate dehydrogenase, mitochondrial	0.336275841	0.043256183	0.001905656

1116	60S ribosomal protein L10a	0.341061701	0.064102701	0.005751534
1117	Protein RGD1562310	0.333383078	0.025364432	0.000402465
1118	Autophagy-related protein 9A	0.334984664	0.037244995	0.001250012
1119	Alpha glucosidase 2 alpha neutral subunit (Predicted)	0.332762294	0.038043316	0.001331632
1120	Single Ig IL-1-related receptor	0.332888509	0.03945791	0.00148042
1121	Transmembrane emp24 domain-containing protein 3	0.329920861	0.01654782	0.000114711
1122	Acyl carrier protein	0.341016804	0.073655331	0.009212163
1123	Polypeptide N-acetylgalactosaminyltransferase 1	0.330676599	0.030674298	0.000714555
1124	Mitochondrial ribosomal protein S21 (Predicted), isoform CRA_a	0.332291896	0.041196745	0.001701024
1125	Mitochondrial-processing peptidase subunit alpha	0.332436535	0.042579929	0.001960294
1126	Protein LOC100911440	0.327020791	0.019426881	0.000187566
1127	Carbohydrate sulfotransferase 11	0.330172952	0.042821568	0.001900797
1128	60S ribosomal protein L13a	0.330451405	0.045565915	0.002272041
1129	Protein Ppm1l (Fragment)	0.3274369	0.027232995	0.000510374
1130	Protein Acad8 (Fragment)	0.325619502	0.02153836	0.000251782
1131	Protein Tmem87a	0.330252541	0.050061664	0.00329355
1132	Stomatin-like protein 2, mitochondrial	0.325949793	0.027394087	0.000522978
1133	PRA1 family protein 3	0.329884785	0.053961867	0.003698538
1134	Anoctamin	0.328369907	0.046992805	0.002526401
1135	Serine protease HTRA1	0.324668808	0.030535262	0.000724931
1136	Microfibril-associated glycoprotein 3	0.329228578	0.054462711	0.003855134
1137	Protein Naglu	0.325755635	0.043341844	0.002008385
1138	Ras-related protein Rab-12	0.321614564	0.014635342	8.10277E-05
1139	Cytochrome b-c1 complex subunit 6, mitochondrial	0.323646326	0.036493704	0.001229518
1140	Pleckstrin homology domain containing, family B (Evectins) member 2 (Predicted), isoform CRA_b	0.322542011	0.031788108	0.000821633
1141	Dihydropyrimidinase-related protein 1	0.322001395	0.033936287	0.001122616
1142	Leucine-rich repeat-containing protein 8A	0.32272377	0.04216133	0.002033678
1143	Protein Nfxl1	0.32013571	0.028931414	0.000631894
1144	Mitochondrial carnitine/acylcarnitine carrier protein	0.319397901	0.028951815	0.000622758
1145	Polyribonucleotide nucleotidyltransferase 1	0.317973835	0.015587548	0.000101632
1146	Protein Rictor (Fragment)	0.325465782	0.059702721	0.005118366

1147	Protein Srpr	0.320319302	0.040239651	0.001692685
1148	Protein Tmx4	0.325579929	0.065338961	0.006451167
1149	Protein Sec61a2	0.319802137	0.042332522	0.001995433
1150	Protein Afg3l1	0.323271208	0.0600278	0.004954823
1151	Protein LOC100359642	0.317362154	0.030984318	0.000784096
1152	40S ribosomal protein S17	0.317435961	0.031971767	0.000859607
1153	Sec11-like 3 (S. cerevisiae), isoform CRA_c	0.315165494	0.018124068	0.000161639
1154	Protein Myof	0.315513024	0.024936634	0.000416657
1155	Protein Col4a2	0.314301496	0.014591572	8.01273E-05
1156	Nucleolar protein 5A	0.321885178	0.060172882	0.005216623
1157	NADH dehydrogenase [ubiquinone] iron-sulfur protein 2, mitochondrial	0.319105911	0.04933297	0.003041367
1158	60S ribosomal protein L10	0.325152614	0.071980354	0.008711082
1159	Disintegrin and metalloproteinase domain-containing protein 10 (Fragment)	0.31528679	0.040765926	0.00170826
1160	Protein Lclat1	0.315279896	0.04049555	0.001717256
1161	Protein B4galt7	0.324400003	0.075884299	0.009768395
1162	Protein OS-9	0.320169026	0.062978037	0.006039178
1163	Cytochrome c oxidase subunit 6B1	0.316367917	0.051177065	0.003141082
1164	Protein Aifm2	0.313875827	0.041003956	0.001744402
1165	ATPase type 13A1 (Predicted), isoform CRA_a	0.3167676	0.053716338	0.003907447
1166	DnaJ (Hsp40) homolog, subfamily C, member 11	0.31117407	0.024416621	0.000400277
1167	Isoform Short of Insulin receptor	0.315642237	0.050507834	0.003245785
1168	Mitochondrial ribosomal protein L21 (Predicted), isoform CRA_a	0.318656377	0.062052351	0.006045061
1169	LRRGT00066	0.317525516	0.059368821	0.005193125
1170	Protein Tmem181	0.311060584	0.033085117	0.000973164
1171	Protein disulfide-isomerase A5	0.311103353	0.033554236	0.001038941
1172	ATP-binding cassette sub-family G member 2	0.31736759	0.067688146	0.007867223
1173	Protein Hars2	0.307571102	0.023279908	0.000375396
1174	Soluble calcium-activated nucleotidase 1	0.309118609	0.042652597	0.00209776
1175	Protein LOC100360426	0.30512566	0.016536087	0.000129687
1176	NADH dehydrogenase (Ubiquinone) 1 alpha subcomplex, 12 (Predicted), isoform CRA_b	0.311408752	0.056329529	0.004557441

1177	Mannosyl-oligosaccharide glucosidase	0.305747086	0.027959251	0.00061522
1178	Endoplasmic reticulum resident protein 29	0.307606638	0.041620968	0.001992518
1179	40S ribosomal protein S4, X isoform	0.309037612	0.048400591	0.002995718
1180	Prolyl 4-hydroxylase subunit alpha-1	0.307737087	0.04283252	0.002141722
1181	Protein Trappc6b	0.315468058	0.073289946	0.009476149
1182	ATPase, H+ transporting, V1 subunit D, isoform CRA_c	0.309017299	0.053736357	0.004062393
1183	Protein Mfsd10	0.304079478	0.033500029	0.001074569
1184	ATPase family AAA domain-containing protein 1	0.305737382	0.043671442	0.002267345
1185	Protein Nomo1	0.308575574	0.055793244	0.004499307
1186	NADH dehydrogenase [ubiquinone] 1 alpha subcomplex subunit 9, mitochondrial	0.303046165	0.029120396	0.000704013
1187	Inhibitor of nuclear factor kappa-B kinase-interacting protein	0.302507304	0.031428736	0.000834208
1188	Inhibitor of nuclear factor kappa-B kinase-interacting protein	0.302507304	0.031428736	0.000834208
1189	Protein RGD1305350	0.299975758	0.006884741	9.89002E-06
1190	Endothelial lipase	0.305704266	0.049462818	0.003232799
1191	Ab2-417	0.300688945	0.024887481	0.000446637
1192	Solute carrier family 12 member 4	0.300300224	0.029349364	0.00073158
1193	DnaJ homolog subfamily B member 11	0.299337538	0.025217799	0.000477055
1194	Transmembrane emp24 domain-containing protein 7	0.300763453	0.03506552	0.001230567
1195	DnaJ homolog subfamily C member 3	0.309179098	0.068823929	0.008239703
1196	RAB14, member RAS oncogene family	0.305568412	0.059011159	0.005450805
1197	Tyrosine-protein phosphatase non-receptor type 1	0.297338812	0.011663944	4.85882E-05
1198	Protein Tmtc3	0.3028869	0.048854775	0.003184194
1199	Protein Sdf2l1	0.302404877	0.050619222	0.003474243
1200	Casein kinase II subunit beta	0.299981458	0.041301801	0.001990945
1201	Probable lysosomal cobalamin transporter	0.300017859	0.041562967	0.002027067
1202	Protein Tex264	0.307257774	0.073595217	0.009635012
1203	Sodium- and chloride-dependent taurine transporter	0.294787287	0.028326817	0.000680069
1204	Transferrin receptor protein 1	0.296605923	0.041283222	0.002045882
1205	Procollagen-lysine,2-oxoglutarate 5-dioxygenase 2	0.2981002	0.048762589	0.003245483
1206	Redox-regulatory protein FAM213A	0.29847595	0.051685489	0.003823037
1207	Aa2-277	0.291837801	0.008598518	1.98798E-05

1208	Phosphatidylinositol glycan, class K	0.296396059	0.045493159	0.002792556
1209	CD82 antigen	0.296569414	0.047119546	0.002967257
1210	Uncharacterized protein	0.296874655	0.049438706	0.003448893
1211	Glycerol-3-phosphate dehydrogenase, mitochondrial	0.294355942	0.039003096	0.001740653
1212	Coagulation factor II, isoform CRA_a	0.291179783	0.024530523	0.000461028
1213	Procollagen C-endopeptidase enhancer 1	0.301535615	0.071724699	0.009208064
1214	Mtch1 protein	0.290780193	0.028568709	0.000693938
1215	40S ribosomal protein S15a	0.295097167	0.051386512	0.003904941
1216	WD repeat-containing protein 81	0.288433703	0.004849983	3.63485E-06
1217	Phosphatidylinositol glycan, class T (Predicted), isoform CRA_b	0.300714834	0.070615625	0.008975333
1218	Protein Slc27a4	0.299441087	0.067658065	0.007833426
1219	Peroxisomal biogenesis factor 3	0.291277136	0.034949875	0.00119291
1220	Isoform 2 of Reticulon-4	0.294696922	0.05322871	0.004243813
1221	Phosphatidylinositol glycan anchor biosynthesis class U protein	0.292963716	0.050115759	0.003611839
1222	Protein LOC100910481	0.298906387	0.072026322	0.009603865
1223	Uncharacterized protein (Fragment)	0.28877682	0.036212491	0.001449285
1224	V-type proton ATPase subunit C 1	0.286035385	0.026654035	0.000601052
1225	Succinyl-CoA:3-ketoacid coenzyme A transferase 1, mitochondrial	0.295672479	0.068329584	0.008748734
1226	Uncharacterized protein (Fragment)	0.287919743	0.040683126	0.00204347
1227	60S ribosomal protein L18a	0.284228706	0.018244309	0.000192663
1228	CDGSH iron-sulfur domain-containing protein 1	0.291745019	0.060481295	0.006180718
1229	Hexokinase-1	0.285305803	0.033389142	0.001350928
1230	Synaptojanin-2-binding protei	0.285543794	0.042591223	0.002427309
1231	Protein NDRG1	0.285819563	0.045297109	0.002821953
1232	Hypoxia up-regulated protein 1	0.28012017	0.012887619	7.10605E-05
1233	ATP-binding cassette sub-family D member 3 (Fragment)	0.286712692	0.052075065	0.004270356
1234	1-acylglycerol-3-phosphate O-acyltransferase ABHD5	0.281631858	0.028539402	0.000759812
1235	Procollagen-lysine,2-oxoglutarate 5-dioxygenase 3	0.287123297	0.055138441	0.004845745
1236	Protein Apoo	0.285514509	0.053048937	0.004435885
1237	Aconitate hydratase, mitochondrial	0.284602678	0.050729527	0.0039489
1238	Protein Pric285	0.278344224	0.0166988	0.000151865
1239	Protein YIPF4	0.287057317	0.063532696	0.007055007

1240	Ras-related protein Rab-9A	0.279825886	0.037226147	0.001660471
1241	Integrin beta	0.276160144	0.008737455	2.17057E-05
1242	Protein Afg3l2	0.278838782	0.033538457	0.001213493
1243	Protein Antxr2	0.286305885	0.066659499	0.008344059
1244	Protein Atg2a	0.287715694	0.070440396	0.009691921
1245	Protein Trappc11	0.276317399	0.02568529	0.000571443
1246	Nicotinamide nucleotide transhydrogenase	0.276592665	0.029399988	0.000849634
1247	Dihydrolipoyl dehydrogenase, mitochondrial	0.280526338	0.048856967	0.003682381
1248	Peptidyl-tRNA hydrolase 2	0.281299943	0.054442961	0.004957447
1249	Phosphatidylinositol 4-kinase type 2-beta	0.276260243	0.033156777	0.001145989
1250	DnaJ homolog subfamily C member 10	0.282391932	0.062108003	0.006899591
1251	Protein Slc25a12 (Fragment)	0.27855733	0.048135397	0.003637712
1252	Long-chain-fatty-acid--CoA ligase 1	0.285120544	0.070611164	0.00967707
1253	Protein Nup160	0.281489388	0.060249835	0.006495903
1254	Glycosyltransferase 25 domain containing 1	0.276492585	0.041474438	0.00229486
1255	Protein Pxdn	0.272886818	0.021062605	0.000342719
1256	Cell adhesion molecule 1	0.274502745	0.034243367	0.001347379
1257	Protein O-fucosyltransferase 2 (Predicted), isoform CRA_a	0.270401551	0.002033477	3.01826E-07
1258	Alpha-1,3-mannosyl-glycoprotein 2-beta-N-acetylglucosaminyltransferase	0.272325141	0.030760989	0.001018617
1259	Dolichyl-diphosphooligosaccharide--protein glycosyltransferase subunit DAD1	0.281181607	0.065416436	0.008771415
1260	Protein Stx16	0.273100751	0.038907085	0.001959381
1261	Protein RGD1566265	0.270913007	0.028777752	0.000812552
1262	Sodium-coupled neutral amino acid transporter 1	0.270367827	0.029590011	0.000902924
1263	Golgi apparatus protein 1	0.271171977	0.043720851	0.002762557
1264	Similar to oxysterol-binding protein-like protein 8 isoform a	0.268948157	0.034504975	0.001402121
1265	Pcyox1l protein	0.268982107	0.034850071	0.001444283
1266	Ufm1-specific protease 2	0.270440182	0.042899455	0.002568945
1267	Amine oxidase [flavin-containing] A	0.270481512	0.043095838	0.002697694
1268	Nucleobindin 2, isoform CRA_b	0.266838021	0.023369471	0.00045513
1269	Glutaminase kidney isoform, mitochondrial	0.269798858	0.042586609	0.002493858

1270	Protein Galnt2	0.266154465	0.030522536	0.000999268
1271	NADH dehydrogenase (Ubiquinone) 1 beta subcomplex, 6 (Predicted)	0.274660524	0.067316211	0.008617565
1272	NADH dehydrogenase (Ubiquinone) Fe-S protein 7	0.268566171	0.047744514	0.003272862
1273	Protein Rmnd1	0.264893993	0.030231901	0.000893091
1274	Dephospho-CoA kinase domain-containing protein	0.266481735	0.039909114	0.002183753
1275	Unconventional myosin-X	0.26236543	0.019555173	0.000280562
1276	Transmembrane 9 superfamily member 4	0.262448405	0.02097493	0.000347182
1277	Rab3 GTPase-activating protein non-catalytic subunit	0.263055481	0.029479717	0.000940245
1278	Protein Serinc3	0.260836496	0.012402831	7.29834E-05
1279	Glutamate dehydrogenase 1, mitochondrial	0.265737029	0.043720472	0.0029039
1280	Lactamase, beta (Predicted)	0.271991278	0.065161501	0.008582022
1281	Ras-related protein Rap-1b	0.26116309	0.019534305	0.000280516
1282	Protein Sppl2a	0.269500922	0.061777031	0.007510527
1283	Mitochondrial ribosomal protein S24	0.266346787	0.052871537	0.005064813
1284	UDP-glucose:glycoprotein glucosyltransferase 1	0.25926722	0.017525314	0.000211452
1285	Putative sodium-coupled neutral amino acid transporter 7	0.25926722	0.017525314	0.000211452
1286	Protein Agk	0.258573092	0.025432427	0.000639308
1287	Pituitary tumor-transforming gene 1 protein-interacting protein	0.263557727	0.049774319	0.004247653
1288	Mitochondrial ribosomal protein L44	0.261746081	0.045016359	0.003277117
1289	Protein Edem3	0.257227917	0.022938493	0.000502683
1290	Galectin-3-binding protein	0.258538808	0.032193392	0.00125825
1291	Delta(24)-sterol reductase	0.25889929	0.035797005	0.001699809
1292	Protein Sptlc2	0.255467543	0.011625271	6.13717E-05
1293	Protein sel-1 homolog 1	0.26602886	0.065002024	0.008775312
1294	Tubulin beta-2B chain	0.266089722	0.065350414	0.008916146
1295	Mitochondrial import inner membrane translocase subunit Tim9	0.254185632	0.007747304	1.85779E-05
1296	Nicastrin	0.261581975	0.052387824	0.005067466
1297	Neutral cholesterol ester hydrolase 1	0.258494946	0.042528965	0.002824208
1298	Protein Pgs1	0.262213679	0.056423256	0.006098895
1299	ERO1-like protein alpha	0.254142207	0.020643112	0.00035052
1300	Glycerol-3-phosphate acyltransferase 3	0.252757611	0.016610648	0.000185613
1301	BAK protein	0.253931624	0.026068446	0.000692775

1302	Guanine nucleotide-binding protein subunit alpha-13	0.254154111	0.028902322	0.000929147
1303	Synaptogyrin-1	0.254212433	0.029523184	0.000989122
1304	LETM1 and EF-hand domain-containing protein 1, mitochondrial	0.258625345	0.05210492	0.005230167
1305	Protein LOC100362504	0.259108291	0.056843306	0.005167337
1306	Isoform 2 of NADH-cytochrome b5 reductase 3	0.251252079	0.02075615	0.000359442
1307	Guanine nucleotide-binding protein G(s) subunit alpha isoforms short	0.262231493	0.067136586	0.009900715
1308	60S ribosomal protein L32	0.261632784	0.066210789	0.009860933
1309	2-oxoglutarate dehydrogenase, mitochondrial	0.256434524	0.052864578	0.005323691
1310	Protein Rab6b	0.252438829	0.040185232	0.002509745
1311	Cytochrome c oxidase subunit 5B, mitochondrial	0.248087252	0.015141125	0.000143138
1312	Protein Prkcsh	0.253023938	0.044811707	0.003416725
1313	Ac1164	0.247572539	0.02529664	0.000673906
1314	Cytochrome b-c1 complex subunit 8	0.254590477	0.055770894	0.006268925
1315	Atlastin-3	0.248385975	0.034395951	0.001649704
1316	LEM domain containing 2	0.246127571	0.020859761	0.000370822
1317	ORM1-like protein 3	0.245180129	0.013464151	9.9754E-05
1318	Protein Dpy19l1	0.254833805	0.060146544	0.008036765
1319	Mannose-P-dolichol utilization defect 1	0.251012717	0.050431175	0.004963269
1320	Lysosome-associated membrane glycoprotein 1	0.243284893	0.006467293	1.19317E-05
1321	Amyloid-like protein 2 (Fragment)	0.255008011	0.06409604	0.009227305
1322	Anthrax toxin receptor 1	0.245481599	0.033326087	0.001533614
1323	Nicalin	0.248985287	0.048345624	0.004373834
1324	Immunity-related GTPase family M protein	0.245278218	0.037215786	0.001880905
1325	Protein Sun2	0.247781261	0.047583288	0.004174073
1326	Cystatin-C	0.249217433	0.053384281	0.005702795
1327	60S ribosomal protein L9	0.242159444	0.027449877	0.000896061
1328	Ndufa7 protein	0.24227174	0.034550151	0.001723889
1329	Monoacylglycerol lipase ABHD12	0.240223939	0.02393468	0.000598158
1330	Protein Stt3b	0.240337711	0.025355527	0.0007325
1331	Isoform 2 of Inhibitor of nuclear factor kappa-B kinase-interacting protein	0.245170571	0.048530365	0.004531906
1332	Protein Aldh18a1	0.239848674	0.026159241	0.000792782

1333	Calcium-binding mitochondrial carrier protein SCaMC-2	0.244203615	0.049662239	0.004735782
1334	Aldehyde dehydrogenase, mitochondrial	0.240009556	0.033670988	0.001658087
1335	Sigma non-opioid intracellular receptor 1	0.244617825	0.051446373	0.006165944
1336	Protein disulfide-isomerase	0.237156667	0.018886515	0.00030922
1337	Protein Esyt2	0.237903615	0.028894896	0.001057822
1338	Isoform 3 of Agrin	0.23580966	0.01383863	0.00012389
1339	NADH dehydrogenase (Ubiquinone) Fe-S protein 8 (Predicted), isoform CRA_a	0.245092831	0.058127895	0.007470897
1340	Isoform 2 of Trimethyllysine dioxygenase, mitochondrial	0.234445357	0.003762942	2.44719E-06
1341	Protein Atp6v1h	0.244320461	0.060142497	0.008665055
1342	Ras-related protein Ral-A	0.239285437	0.045471758	0.004043369
1343	Sulfated glycoprotein 1	0.234446409	0.026142281	0.000859279
1344	Protein Slc25a24	0.234619918	0.028270979	0.001041879
1345	Glycosylphosphatidylinositol anchor attachment protein 1 homolog (Yeast)	0.237780139	0.046222857	0.004052439
1346	Stress-70 protein, mitochondrial	0.238400063	0.050324148	0.005165067
1347	Magnesium transporter protein 1	0.231396518	0.020964429	0.000468924
1348	Protein Slc25a17	0.233789623	0.040096944	0.002865382
1349	Collagenase 3	0.231281141	0.031897997	0.001602151
1350	Protein Emc4	0.231351591	0.032790563	0.001630833
1351	Receptor expression-enhancing protein 5	0.23251032	0.038165825	0.002570285
1352	Ferritin light chain 1	0.23922545	0.06081263	0.009380655
1353	Isocitrate dehydrogenase [NADP], mitochondrial	0.234014871	0.045602379	0.004245419
1354	Isoform SERCA2A of Sarcoplasmic/endoplasmic reticulum calcium ATPase 2	0.228022304	0.017623709	0.00027808
1355	Plasminogen receptor (KT)	0.229282805	0.032907894	0.001736533
1356	LRRGT00013	0.232595865	0.04744111	0.004221355
1357	3-keto-steroid reductase	0.229763227	0.037060161	0.002398802
1358	Protein RGD1310313	0.22689557	0.024132315	0.000672969
1359	Short/branched chain specific acyl-CoA dehydrogenase, mitochondrial	0.226536125	0.025898423	0.000863134
1360	Coiled-coil domain-containing protein 47	0.224656707	0.012687971	0.000105207
1361	Ras-related protein Rab-31	0.226177186	0.027609135	0.001073053

1362	ATP synthase-coupling factor 6, mitochondrial	0.226109017	0.032139174	0.001647098
1363	40S ribosomal protein S3	0.234801684	0.062135422	0.009805961
1364	Protein Aldh1l2	0.226609811	0.036713087	0.002329142
1365	7-dehydrocholesterol reductase	0.223508572	0.019852551	0.000415291
1366	40S ribosomal protein S9	0.222827963	0.017172149	0.000265782
1367	D-serine modulator-1	0.221916092	0.007555708	2.30404E-05
1368	Perilipin	0.221939763	0.008441535	3.17418E-05
1369	ATPase, H+ transporting, V1 subunit E isoform 1, isoform CRA_a	0.22433977	0.030022294	0.001323442
1370	Vesicle-associated membrane protein-associated protein A	0.224420761	0.030765931	0.001463088
1371	Protein Acot13	0.232005703	0.058715491	0.008783813
1372	Protein LOC100910109 (Fragment)	0.228478012	0.049646375	0.005767056
1373	Transmembrane 9 superfamily member 2	0.221515868	0.026987415	0.000986203
1374	Annexin A2 GN=Anxa2 PE=1 SV=2	0.225841245	0.045810301	0.004542335
1375	Calcineurin B homologous protein 1	0.219919405	0.019335766	0.000389769
1376	Apolipoprotein O-like	0.228626263	0.057362341	0.007876869
1377	Alanyl (Membrane) aminopeptidase	0.220292248	0.042372034	0.003761724
1378	Protein ERGIC-53	0.214863119	0.018110511	0.000339085
1379	Phosphatidylinositide phosphatase SAC1	0.218266568	0.037943282	0.002902887
1380	Cytoskeleton-associated protein 4 (Predicted)	0.217568809	0.04012524	0.003367533
1381	Acyl-CoA thioesterase 9	0.214539239	0.031335899	0.001765654
1382	Lanosterol synthase	0.212783194	0.023207327	0.000715517
1383	Protein Slc25a13 (Fragment)	0.217080495	0.043438579	0.004242586
1384	H(+)/Cl(-) exchange transporter 5	0.214974269	0.039083698	0.003151884
1385	Protein Myo6	0.213116477	0.031676275	0.001762599
1386	Synaptic vesicle membrane protein VAT-1 homolog	0.216144364	0.043720392	0.004315902
1387	Signal peptidase complex subunit 3	0.210412951	0.017070618	0.000295054
1388	Protein Stt3a	0.209945348	0.017264636	0.000310822
1389	Monocarboxylate transporter 4	0.212379442	0.033834651	0.002076844
1390	Protein Fat1	0.211414485	0.033464913	0.002166211
1391	Extracellular matrix protein 1	0.2102465	0.0316909	0.001856491
1392	ATP synthase subunit e, mitochondrial	0.208334336	0.021628003	0.000606377
1393	Heat shock 27kDa protein 1 GN=Hspb1 PE=3 SV=1	0.20857869	0.024632447	0.000877926

1394	Protein Pnpla6	0.216496042	0.054405284	0.008125487
1395	Sodium/potassium-transporting ATPase subunit alpha-1	0.207158731	0.018548334	0.000385029
1396	Cytochrome b-c1 complex subunit Rieske, mitochondrial	0.208209982	0.025866742	0.001000437
1397	Procollagen-proline, 2-oxoglutarate 4-dioxygenase (Proline 4-hydroxylase), alpha II polypeptide (Predicted), isoform CRA_a	0.210147192	0.035085325	0.002385201
1398	Ras-related protein Rab-6A	0.211486836	0.041620003	0.003892621
1399	Transmembrane emp24 domain-containing protein 9	0.212316	0.046895928	0.005625852
1400	Peroxiredoxin-4	0.207524193	0.028526594	0.001335745
1401	2-oxoglutarate and iron-dependent oxygenase domain-containing protein 3	0.209885617	0.039929632	0.003604543
1402	Heat shock 70 kDa protein 13	0.204544385	0.014221757	0.000181185
1403	Membrane-associated progesterone receptor component 2	0.213711386	0.054599417	0.008246291
1404	Isoform 2 of Reticulon-3	0.204451017	0.020222164	0.00051102
1405	40S ribosomal protein S26	0.21411795	0.056526008	0.009096634
1406	Protein Dnajc13	0.204633744	0.022593187	0.000705807
1407	Ras-related protein R-Ras	0.211102263	0.050919685	0.006916331
1408	Nucleobindin-1	0.208290254	0.042291809	0.004133511
1409	A disintegrin and metalloproteinase with thrombospondin motifs 1	0.202776382	0.016026466	0.000265298
1410	1-acylglycerol-3-phosphate O-acyltransferase 3 (Predicted), isoform CRA_b	0.202896653	0.017947139	0.00037092
1411	Low-density lipoprotein receptor-related protein 10	0.204889002	0.03459905	0.002196495
1412	SPRY domain-containing protein 7	0.200585369	0.008595724	4.231E-05
1413	Cytochrome c oxidase subunit 5A, mitochondrial	0.200832178	0.014365222	0.000195674
1414	Protein Nt5dc3	0.208388903	0.048137232	0.006192747
1415	Histocompatibility 13 (Predicted), isoform CRA_a	0.205767061	0.04038254	0.004041856
1416	Succinate dehydrogenase [ubiquinone] cytochrome b small subunit, mitochondrial	0.200280216	0.012516658	0.000131673
1417	ADP/ATP translocase 1	0.207073572	0.045861636	0.005428091
1418	Solute carrier family 35 member F6	0.200726692	0.019905553	0.000513652
1419	Prostaglandin G/H synthase 1	0.200882335	0.021873293	0.000689762
1420	40S ribosomal protein S16	0.210481968	0.057838128	0.009552979
1421	1-acyl-sn-glycerol-3-phosphate acyltransferase delta	0.199136626	0.006079857	1.49449E-05

1422	Long-chain specific acyl-CoA dehydrogenase, mitochondrial	0.204858813	0.040501244	0.00416097
1423	NADPH--cytochrome P450 reductase	0.20610509	0.045671702	0.005360373
1424	Sorting and assembly machinery component 50 homolog	0.198868366	0.011704155	0.000109359
1425	Uncharacterized protein C17orf62 homolog	0.198885015	0.019675568	0.00051617
1426	Guanine nucleotide-binding protein G(i) subunit alpha-2	0.198080158	0.014279728	0.00019138
1427	Bone morphogenetic protein 1, isoform CRA_b	0.200912025	0.031359734	0.001963496
1428	Protein Lamc1	0.20245676	0.039782865	0.003755862
1429	Protein Tmx1	0.205453829	0.054336377	0.00958564
1430	3-ketoacyl-CoA thiolase A, peroxisomal	0.195656957	0.018728338	0.000456463
1431	ATP synthase subunit f, mitochondrial	0.197126709	0.029952138	0.00175335
1432	Mitochondrial import receptor subunit TOM40 homolog	0.194026501	0.014125642	0.000198089
1433	Sideroflexin 3	0.194545013	0.026440394	0.001278544
1434	Asparagine-linked glycosylation 5 homolog (Yeast, dolichyl-phosphate beta-glucosyltransferase), isoform CRA_a	0.193305189	0.022631658	0.000820983
1435	Dihydrolipoamide S-succinyltransferase (E2 component of 2-oxo-glutarate complex), isoform CRA_a	0.191785987	0.013608607	0.000180348
1436	Ribosomal protein S2	0.190368064	0.01152411	0.000112118
1437	Peroxisomal multifunctional enzyme type 2	0.189388092	0.00879451	5.25578E-05
1438	Transmembrane protein 11, mitochondrial	0.197776709	0.055977766	0.009938107
1439	Prohibitin	0.186325601	0.007646234	3.42294E-05
1440	ADP/ATP translocase 2	0.190682085	0.034507122	0.002843399
1441	Very-long-chain enoyl-CoA reductase	0.188588167	0.027913703	0.001493097
1442	Calreticulin	0.186190678	0.014370942	0.000219409
1443	Fatty acyl-CoA reductase 1	0.188100445	0.027439313	0.001391422
1444	Protein Atp6v1a	0.195222544	0.051378518	0.008427906
1445	Transmembrane emp24 domain-containing protein 5	0.185790998	0.01476947	0.000247069
1446	Mitochondrial ribosomal protein L47	0.192151832	0.044551768	0.005576849
1447	60S ribosomal protein L15	0.190708852	0.040812541	0.004428472
1448	Erlin-2	0.184384757	0.018997026	0.000531423
1449	Cytochrome b-c1 complex subunit 2, mitochondrial	0.182475615	0.006432586	2.32601E-05
1450	Signal sequence receptor, alpha	0.184477148	0.024814083	0.001177003
1451	Insulin-like growth factor 2 receptor, isoform CRA_b	0.182836672	0.014780474	0.000260644

1452	Annexin	0.191452223	0.051978528	0.009133032
1453	DnaJ homolog subfamily C member 5	0.180741836	0.003428349	3.43859E-06
1454	Angiopoietin-related protein 4	0.180500646	0.009782105	7.92973E-05
1455	Voltage-dependent anion-selective channel protein 3	0.183394026	0.033492695	0.002681042
1456	60S ribosomal protein L27a	0.181688822	0.025839504	0.001423414
1457	Protein Tapbp	0.182541741	0.032819252	0.002880154
1458	Glycerophosphodiester phosphodiesterase domain-containing protein 1	0.181348257	0.029917055	0.002271998
1459	Ras-related protein Rab-18	0.183074034	0.036552727	0.003964637
1460	Protein Tm9sf3	0.177366996	0.02350169	0.001116251
1461	Uncharacterized protein	0.179898021	0.034675742	0.003285366
1462	Transmembrane protein 106B	0.18167419	0.040602682	0.005148066
1463	Acyl-coenzyme A oxidase	0.177555278	0.025382156	0.001395592
1464	Peroxisomal 2,4-dienoyl-CoA reductase	0.175117675	0.01581766	0.000356696
1465	Sodium/potassium-transporting ATPase subunit beta-1	0.181749359	0.043419117	0.006350454
1466	Cytochrome P450 20A1	0.174690523	0.015590133	0.000311355
1467	Dolichyl-diphosphooligosaccharide--protein glycosyltransferase 48 kDa subunit	0.175795766	0.023782237	0.001227229
1468	Lman2 protein	0.177071292	0.031400823	0.002659438
1469	Protein Dpm1	0.180977657	0.049662635	0.009108268
1470	ATP synthase F(0) complex subunit B1, mitochondrial	0.170492094	0.011223952	0.000134851
1471	Prohibitin-2	0.178715706	0.046475209	0.007895591
1472	Protein Tcirg1	0.169442324	0.00197016	7.4145E-07
1473	Guanine nucleotide-binding protein G(I)/G(S)/G(T) subunit beta-1	0.169003345	0.01250687	0.000194765
1474	Zinc transporter ZIP6	0.168676175	0.01355272	0.00024608
1475	Neutral amino acid transporter ASCT1	0.175406398	0.043135573	0.006583654
1476	Protein Hspg2 (Fragment)	0.169216127	0.020670896	0.000850782
1477	Microsomal glutathione S-transferase 1	0.176514232	0.047313392	0.008092081
1478	B-cell receptor-associated protein 31	0.17092995	0.032297766	0.003018355
1479	Zinc transporter 7	0.171134548	0.033814651	0.003425612
1480	Microtubule-associated proteins 1A/1B light chain 3A	0.165239972	0.004393241	8.89555E-06
1481	Malectin	0.17065335	0.040405132	0.005725582
1482	Protein disulfide-isomerase A3	0.165447338	0.028014647	0.00212811

1483	Isoform 2 of 45 kDa calcium-binding protein	0.164208915	0.032660853	0.003224378
1484	Interferon-related developmental regulator 1	0.159418486	0.009167581	8.77713E-05
1485	Protein Pcdh1 (Fragment)	0.166657275	0.04279258	0.007120812
1486	Lanosterol 14-alpha demethylase	0.165591977	0.042926913	0.007208427
1487	A disintegrin and metalloproteinase with thrombospondin motifs 4	0.165132167	0.041983861	0.007125821
1488	Proprotein convertase subtilisin/kexin type 9	0.155178456	0.016411631	0.000538458
1489	Protein Tmx3	0.154007205	0.008933933	8.33518E-05
1490	Dolichyl-diphosphooligosaccharide--protein glycosyltransferase subunit 2	0.152415384	0.016995186	0.000609707
1491	Long-chain-fatty-acid--CoA ligase 3	0.15146849	0.017929122	0.000786649
1492	ATPase, H+ transporting, lysosomal accessory protein 1	0.151270016	0.01969099	0.000937605
1493	Protein Lrp1	0.156826597	0.040981898	0.007358785
1494	Cysteine-rich with EGF-like domain protein 1	0.157101212	0.042690077	0.008016827
1495	Cytochrome c oxidase subunit 7C, mitochondrial	0.150743405	0.021184127	0.001206753
1496	Lysyl oxidase homolog 2	0.150631843	0.023494228	0.001514195
1497	CDP-diacylglycerol--inositol 3-phosphatidyltransferase	0.156172259	0.042838428	0.008374023
1498	Protein YIPF3	0.148054246	0.013812564	0.000355675
1499	2'-5'-oligoadenylate synthase 3 (Fragment)	0.148486845	0.018845417	0.000975143
1500	Tax1-binding protein 1 homolog	0.1485137	0.019306076	0.00097242
1501	Transmembrane emp24 domain-containing protein 10	0.149360102	0.026816505	0.002415706
1502	2-oxoglutarate dehydrogenase, mitochondrial	0.146606532	0.016709382	0.000692882
1503	Protein Gla	0.147202636	0.030415365	0.003494225
1504	Phosphate carrier protein, mitochondrial	0.148356346	0.035535824	0.005411935
1505	ATP synthase subunit epsilon, mitochondrial	0.142279663	0.01182906	0.000263461
1506	Calcium-binding and coiled-coil domain-containing protein 1	0.14607829	0.033686806	0.004806978
1507	Translocon-associated protein subunit delta	0.14034186	0.004747414	1.71142E-05
1508	Protein Zmpste24	0.146183355	0.037407186	0.006700262
1509	Dolichyl-diphosphooligosaccharide--protein glycosyltransferase subunit 1	0.137896091	0.020404684	0.001305644
1510	Integral membrane protein 2C	0.137280947	0.017367582	0.000833003
1511	Solute carrier family 2, facilitated glucose transporter member 1	0.135990577	0.013067664	0.000373827
1512	Transmembrane emp24 domain-containing protein 2	0.138652633	0.026360766	0.002755327

1513	Protein Spcs2	0.137746762	0.026910477	0.002742131
1514	2'-5'-oligoadenylate synthase-like protein 1	0.137627805	0.027925503	0.003371689
1515	Carboxypeptidase D	0.134935161	0.018618534	0.001095539
1516	Protective protein for beta-galactosidase	0.134450477	0.016860043	0.000818291
1517	Heparan sulfate 2-O-sulfotransferase 1, isoform CRA_a	0.134152423	0.019845798	0.001380783
1518	Cathepsin D	0.132117796	0.014459856	0.000545506
1519	Estradiol 17-beta-dehydrogenase 12	0.129169215	0.004010769	1.32006E-05
1520	Tricarboxylate transport protein, mitochondrial	0.129639059	0.013468723	0.000435136
1521	Calnexin	0.133840968	0.033896021	0.006262283
1522	Protein Hspg2	0.129187325	0.018076397	0.00112729
1523	Adipocyte enhancer-binding protein 1	0.126802361	0.014455519	0.000602184
1524	Lysophospholipid acyltransferase 5	0.132664988	0.038033892	0.008896
1525	Protein LOC100912469	0.121385905	0.019217029	0.001653541
1526	Sphingosine-1-phosphate lyase 1	0.125826106	0.034837963	0.00861536
1527	Long-chain-fatty-acid--CoA ligase 4	0.118507973	0.008010612	0.000126028
1528	Carboxypeptidase E	0.119686587	0.016192538	0.001061106
1529	Procollagen, type XVIII, alpha 1, isoform CRA_a	0.119376923	0.026326788	0.003970274
1530	Protein Itga5 (Fragment)	0.115021756	0.007930681	0.000133305
1531	Gba protein	0.115963921	0.016520703	0.001113155
1532	Pre-B-cell leukemia transcription factor-interacting protein 1	0.114420017	0.016771444	0.001195224
1533	Isoform 4 of Fibronectin	0.114036383	0.015481766	0.001002391
1534	Lipocalin 7, isoform CRA_a	0.114162962	0.01893238	0.001755954
1535	Cytochrome c oxidase subunit 2	0.117543885	0.03537454	0.009497943
1536	Voltage-dependent anion-selective channel protein 2	0.098391053	0.021597421	0.003957607
1537	LRRGT00111	0.098873586	0.026950693	0.006608596
1538	Integral membrane protein 2B	0.100481112	0.031068321	0.009797014
1539	Voltage-dependent anion-selective channel protein 1	0.093880715	0.009218354	0.000346735
1540	Cysteine rich protein 61	0.091088524	0.013237849	0.001096327
1541	ATPase, H+ transporting, lysosomal 38kDa, V0 subunit d1	0.088824765	0.013012936	0.001131621
1542	Renin receptor	0.086883016	0.0094504	0.000458463
1543	Renin receptor	0.086883016	0.0094504	0.000458463
1544	Tissue-type plasminogen activator	0.079416109	0.005946645	0.000142997

1545	Metalloproteinase inhibitor 2	0.076093183	0.00910026	0.000592243
1546	V-H+ATPase subunit a1-III	0.07655637	0.01456211	0.002350189
1547	Sterol-4-alpha-carboxylate 3-dehydrogenase, decarboxylating	0.074248582	0.009525556	0.000709465
1548	Neuraminidase 1	0.075245289	0.016997451	0.003791982
1549	Rho-related GTP-binding protein RhoB	0.068116382	0.002672037	2.08802E-05
1550	Sodium-coupled neutral amino acid transporter 2	0.065405474	0.004034772	7.55119E-05
1551	CD44 antigen	0.062853971	0.016409706	0.005417073
1552	Beta-2-glycoprotein 1	0.059965924	0.011332803	0.002188258
1553	Plasminogen	0.054481153	0.006377207	0.000518809
1554	Transmembrane glycoprotein NMB	0.05349425	0.006362584	0.000530669
1555	Matrix metalloproteinase-14	0.052998134	0.004678672	0.000212981
1556	Apolipoprotein D	0.054602981	0.016043312	0.007260869
1557	Phospholipase D3	0.046125875	0.003520227	0.000137555
1558	Macrophage metalloelastase	0.044926831	0.008091912	0.002129082
1559	Protein F5	0.044380543	0.010023322	0.003119122
1560	Lactadherin	0.043742877	0.012770405	0.007051582
1561	RCG31835	0.040526973	0.007574959	0.001941922
1562	Sequestosome-1	0.038517105	0.002392047	8.79849E-05
1563	Stromelysin-1	0.039689047	0.008586185	0.003056973
1564	Prostaglandin G/H synthase 2	0.038651058	0.007463515	0.002156615
1565	V-type proton ATPase 16 kDa proteolipid subunit	0.03875885	0.008294571	0.002738324
1566	Syndecan-4	0.034844885	0.003963168	0.000470738

Supplementary Table 2 KEGG pathway of dioscin to inhibit HSCs activition

KEGG pathway	Pathway Name	genes quantity
rno01100		189
rno03010	Ribosome	52
rno04141	Protein processing in endoplasmic reticulum	51
rno05016	Huntington's disease	48
rno05010	Alzheimer's disease	44
rno05012	Parkinson's disease	40
rno00190	Oxidative phosphorylation	39
rno03040	Spliceosome	38
rno03013	RNA transport	37
rno01200	Carbon metabolism	36
rno04932	Non-alcoholic fatty liver disease (NAFLD)	32
rno05203	Viral carcinogenesis	28
rno00230	Purine metabolism	27
rno04110	Cell cycle	26
rno04010	MAPK signaling pathway	26
rno05166	HTLV-I infection	24
rno04510	Focal adhesion	24
rno04810	Regulation of actin cytoskeleton	22
rno05200	Pathways in cancer	22
rno04114	Oocyte meiosis	22
rno01230	Biosynthesis of amino acids	22
rno05169	Epstein-Barr virus infection	21
rno05168	Herpes simplex infection	21
rno04151	PI3K-Akt signaling pathway	20
rno04120	Ubiquitin mediated proteolysis	19
rno04015	Rap1 signaling pathway	19
rno05152	Tuberculosis	18
rno00240	Pyrimidine metabolism	17
rno00010	Glycolysis / Gluconeogenesis	17
rno04144	Endocytosis	17
rno04145	Phagosome	17
rno05161	Hepatitis B	16
rno04146	Peroxisome	16
rno05205	Proteoglycans in cancer	16
rno04722	Neurotrophin signaling pathway	16
rno03018	RNA degradation	15
rno04910	Insulin signaling pathway	15
rno04530	Tight junction	15
rno04261	Adrenergic signaling in cardiomyocytes	14
rno04014	Ras signaling pathway	14
rno04260	Cardiac muscle contraction	14
rno04915	Estrogen signaling pathway	14

rno05206	MicroRNAs in cancer	13
rno00510	N-Glycan biosynthesis	13
rno04612	Antigen processing and presentation	13
rno04020	Calcium signaling pathway	13
rno03015	mRNA surveillance pathway	13
rno05211	Renal cell carcinoma	13
rno04520	Adherens junction	13
rno04062	Chemokine signaling pathway	13
rno05164	Influenza A	13
rno04360	Axon guidance	12
rno00480	Glutathione metabolism	12
rno03050	Proteasome	12
rno03030	DNA replication	12
rno04066	HIF-1 signaling pathway	12
rno04310	Wnt signaling pathway	12
rno04142	Lysosome	12
rno01212	Fatty acid metabolism	12
rno04390	Hippo signaling pathway	11
rno00020	Citrate cycle (TCA cycle)	11
rno05034	Alcoholism	11
rno00030	Pentose phosphate pathway	11
rno04012	ErbB signaling pathway	11
rno05145	Toxoplasmosis	11
rno04728	Dopaminergic synapse	11
rno05100	Bacterial invasion of epithelial cells	11
rno05146	Amoebiasis	10
rno04666	Fc gamma R-mediated phagocytosis	10
rno04914	Progesterone-mediated oocyte maturation	10
rno04912	GnRH signaling pathway	10
rno04660	T cell receptor signaling pathway	10
rno03320	PPAR signaling pathway	10
rno00310	Lysine degradation	10
rno04724	Glutamatergic synapse	10
rno04070	Phosphatidylinositol signaling system	10
rno00562	Inositol phosphate metabolism	10
rno05133	Pertussis	10
rno00564	Glycerophospholipid metabolism	10
rno04670	Leukocyte transendothelial migration	10
rno00071	Fatty acid degradation	10
rno00330	Arginine and proline metabolism	10
rno04270	Vascular smooth muscle contraction	10
rno05142	Chagas disease (American trypanosomiasis)	9
rno05140	Leishmaniasis	9
rno00280	Valine, leucine and isoleucine degradation	9

rno05132	Salmonella infection	9
rno05220	Chronic myeloid leukemia	9
rno00250	Alanine, aspartate and glutamate metabolism	9
rno04971	Gastric acid secretion	9
rno03420	Nucleotide excision repair	9
rno04380	Osteoclast differentiation	9
rno04115	p53 signaling pathway	8
rno04720	Long-term potentiation	8
rno04919	Thyroid hormone signaling pathway	8
rno04713	Circadian entrainment	8
rno04726	Serotonergic synapse	8
rno00520	Amino sugar and nucleotide sugar metabolism	8
rno05031	Amphetamine addiction	8
rno04970	Salivary secretion	8
rno05162	Measles	8
rno04540	Gap junction	8
rno03008	Ribosome biogenesis in eukaryotes	8
rno00051	Fructose and mannose metabolism	7
rno04727	GABAergic synapse	7
rno04650	Natural killer cell mediated cytotoxicity	7
rno05222	Small cell lung cancer	7
rno04916	Melanogenesis	7
rno04972	Pancreatic secretion	7
rno04978	Mineral absorption	7
rno04920	Adipocytokine signaling pathway	7
rno04350	TGF-beta signaling pathway	7
rno04721	Synaptic vesicle cycle	7
rno00860	Porphyrin and chlorophyll metabolism	7
rno05323	Rheumatoid arthritis	7
rno05210	Colorectal cancer	7
rno05202	Transcriptional misregulation in cancers	7
rno04668	TNF signaling pathway	7
rno05134	Legionellosis	6
rno00260	Glycine, serine and threonine metabolism	6
rno04512	ECM-receptor interaction	6
rno03060	Protein export	6
rno03022	Basal transcription factors	6
rno05014	Amyotrophic lateral sclerosis (ALS)	6
rno04974	Protein digestion and absorption	6
rno03410	Base excision repair	6
rno00561	Glycerolipid metabolism	6
rno04918	Thyroid hormone synthesis	6
rno05414	Dilated cardiomyopathy (DCM)	6
rno00620	Pyruvate metabolism	6

rno04068	FoxO signaling pathway	6
rno04730	Long-term depression	6
rno05214	Glioma	6
rno04621	NOD-like receptor signaling pathway	6
rno00970	Aminoacyl-tRNA biosynthesis	6
rno04723	Retrograde endocannabinoid signaling	6
rno04370	VEGF signaling pathway	6
rno04150	mTOR signaling pathway	6
rno04976	Bile secretion	6
rno05410	Hypertrophic cardiomyopathy (HCM)	5
rno04961	Endocrine and other factor-regulated calcium reabsorption	5
rno04966	Collecting duct acid secretion	5
rno05212	Pancreatic cancer	5
rno04064	NF-kappa B signaling pathway	5
rno01040	Biosynthesis of unsaturated fatty acids	5
rno05412	Arrhythmogenic right ventricular cardiomyopathy (ARVC)	5
rno04964	Proximal tubule bicarbonate reclamation	5
rno00650	Butanoate metabolism	5
rno00630	Glyoxylate and dicarboxylate metabolism	5
rno00270	Cysteine and methionine metabolism	5
rno00982	Drug metabolism - cytochrome P450	5
rno00360	Phenylalanine metabolism	5
rno05160	Hepatitis C	5
rno04911	Insulin secretion	5
rno00100	Steroid biosynthesis	5
rno05204	Chemical carcinogenesis	5
rno04210	Apoptosis	5
rno00350	Tyrosine metabolism	5
rno01210	2-Oxocarboxylic acid metabolism	4
rno00563	Glycosylphosphatidylinositol(GPI)-anchor biosynthesis	4
rno03020	RNA polymerase	4
rno04725	Cholinergic synapse	4
rno05030	Cocaine addiction	4
rno02010	ABC transporters	4
rno05215	Prostate cancer	4
rno04130	SNARE interactions in vesicular transport	4
rno00670	One carbon pool by folate	4
rno00500	Starch and sucrose metabolism	4
rno04662	B cell receptor signaling pathway	4
rno00380	Tryptophan metabolism	4
rno00052	Galactose metabolism	4
rno00980	Metabolism of xenobiotics by cytochrome P450	4

rno04620	Toll-like receptor signaling pathway	4
rno05032	Morphine addiction	4
rno04744	Phototransduction	4
rno04917	Prolactin signaling pathway	4
rno00514	Other types of O-glycan biosynthesis	4
rno05322	Systemic lupus erythematosus	4
rno04930	Type II diabetes mellitus	3
rno04622	RIG-I-like receptor signaling pathway	3
rno04962	Vasopressin-regulated water reabsorption	3
rno05416	Viral myocarditis	3
rno04913	Ovarian Steroidogenesis	3
rno04610	Complement and coagulation cascades	3
rno04740	Olfactory transduction	3
rno04080	Neuroactive ligand-receptor interaction	3
rno05223	Non-small cell lung cancer	3
rno04623	Cytosolic DNA-sensing pathway	3
rno05020	Prion diseases	3
rno00072	Synthesis and degradation of ketone bodies	3
rno04973	Carbohydrate digestion and absorption	3
rno00590	Arachidonic acid metabolism	3
rno00600	Sphingolipid metabolism	3
rno03430	Mismatch repair	3
rno00512	Mucin type O-glycan biosynthesis	3
rno04514	Cell adhesion molecules (CAMs)	3
rno00565	Ether lipid metabolism	3
rno00062	Fatty acid elongation	3
rno00534	Glycosaminoglycan biosynthesis - heparan sulfate / heparin	3
rno00983	Drug metabolism - other enzymes	3
rno04960	Aldosterone-regulated sodium reabsorption	3
rno04664	Fc epsilon RI signaling pathway	2
rno00740	Riboflavin metabolism	2
rno05213	Endometrial cancer	2
rno00532	Glycosaminoglycan biosynthesis - chondroitin sulfate / dermatan sulfate	2
rno05218	Melanoma	2
rno00400	Phenylalanine, tyrosine and tryptophan biosynthesis	2
rno05219	Bladder cancer	2
rno04742	Taste transduction	2
rno05321	Inflammatiory bowel disease (IBD)	2
rno00471	D-Glutamine and D-glutamate metabolism	2
rno05340	Primary immunodeficiency	2
rno04710	Circadian rhythm	2
rno04330	Notch signaling pathway	2

rno00640	Propanoate metabolism	2
rno00900	Terpenoid backbone biosynthesis	2
rno01220	Degradation of aromatic compounds	2
rno05143	African trypanosomiasis	2
rno05221	Acute myeloid leukemia	2
rno00140	Steroid hormone biosynthesis	2
rno04975	Fat digestion and absorption	2
rno00511	Other glycan degradation	2
rno00592	alpha-Linolenic acid metabolism	2
rno05144	Malaria	2
rno03450	Non-homologous end-joining	2
rno04614	Renin-angiotensin system	2
rno00760	Nicotinate and nicotinamide metabolism	2
rno04672	Intestinal immune network for IgA production	2
rno03440	Homologous recombination	2
rno00460	Cyanoamino acid metabolism	1
rno00340	Histidine metabolism	1
rno04320	Dorso-ventral axis formation	1
rno00790	Folate biosynthesis	1
rno04630	Jak-STAT signaling pathway	1
rno04977	Vitamin digestion and absorption	1
rno05150	Staphylococcus aureus infection	1
rno00450	Selenocompound metabolism	1
rno00410	beta-Alanine metabolism	1
rno00531	Glycosaminoglycan degradation	1
rno00524	Butirosin and neomycin biosynthesis	1
rno00910	Nitrogen metabolism	1
rno04940	Type I diabetes mellitus	1
rno00830	Retinol metabolism	1
rno03460	Fanconi anemia pathway	1
rno04122	Sulfur relay system	1
rno00130	Ubiquinone and other terpenoid-quinone biosynthesis	1
rno00604	Glycosphingolipid biosynthesis - ganglio series	1
rno04060	Cytokine-cytokine receptor interaction	1
rno04640	Hematopoietic cell lineage	1
rno05216	Thyroid cancer	1
rno00040	Pentose and glucuronate interconversions	1
rno00920	Sulfur metabolism	1
rno00120	Primary bile acid biosynthesis	1
rno00430	Taurine and hypotaurine metabolism	1
